# Effects of Digital Health Interventions to Promote Safer Sex Behaviors Among Youth: Systematic Review and Bayesian Network Meta-Analysis

**DOI:** 10.2196/87071

**Published:** 2026-02-04

**Authors:** Yiran Zhu, Wenwen Peng, Die Hu, Edmond Pui Hang Choi, Maritta Anneli Välimäki, Ci Zhang, Xianhong Li

**Affiliations:** 1 Xiangya School of Nursing, Central South University, 172 Tongzipo Road, Changsha, China, 86 731-82650264; 2 School of Nursing, University of Hong Kong, Hong Kong SAR, China (Hong Kong); 3 School of Public Health, Faculty of Medicine, University of Helsinki, Helsinki, Finland; 4 Helsinki University Hospital, Nursing Research Center (NRC), Helsinki, Finland; 5 JBI Xiangya Research Centre for Evidence-based Healthcare Innovation, Central South University, Changsha, China

**Keywords:** digital health intervention, HIV prevention, safer sex behavior, youth, mobile health, telecommunication-based intervention, web-based intervention, network meta-analysis, mHealth

## Abstract

**Background:**

Youth aged 15‐24 years carry a disproportionate HIV/sexually transmitted infections (STIs) burden. In recent years, different modalities of digital health interventions (DHIs) have been explored to promote safer sex behaviors among youth, but their comparative effectiveness across modalities and relative to nondigital interventions (NDIs) remains unclear.

**Objective:**

This study aimed to compare DHI modalities on safer sex behaviors and HIV/STI incidence, rank modalities using Bayesian network meta-analysis (NMA), and position their effectiveness relative to NDIs.

**Methods:**

A systematic review and Bayesian NMA of randomized controlled trials were conducted by comprehensively searching PubMed, EMBASE, Web of Science, and Cochrane Library (inception to November 2025). Eligible studies were those that enrolled youth aged 15‐24 years and evaluated mobile app-based intervention, telecommunication-based intervention (TCI), static web-based intervention (SWI), or interactive online-based intervention (IOI)—with an NDI or another DHI. Primary outcomes were condom use at last sexual contact, consistent condom use, and proportion of condom use. Secondary outcomes included condom use self-efficacy, number of sexual partners, and STI incidence (including HIV). Risk of bias was assessed with the Cochrane Risk of Bias 2 tool, and certainty of evidence with GRADE/CINeMA (Confidence in NMA). Bayesian random-effects NMAs estimated odds ratios (ORs) with 95% credible intervals (CrIs), and complementary frequentist NMAs provided 95% CIs and 95% prediction intervals.

**Results:**

Twenty-four randomized controlled trials (20,134 participants) were included, forming treatment networks across 5 intervention types. TCI was the only intervention that significantly improved condom use at last sex compared with NDI (OR 1.13, 95% CrI 1.02‐1.26). For consistent condom use, SWI and IOI outperformed TCI (SWI vs TCI: OR 1.77, 95% CrI 1.03‐3.06; IOI vs TCI: OR 1.68, 95% CrI 1.02‐2.76). For the proportion of condom use, IOI outperformed SWI (OR 1.34, 95% CrI 1.01‐1.80), and mobile app-based intervention ranked highest in probability rankings, though estimates lacked precision. For STI incidence, NDI was associated with fewer STIs than SWI (OR 0.61, 95% CrI 0.46‐0.82).

**Conclusions:**

This is the first NMA to compare the effectiveness of DHIs on condom use and HIV/STI outcomes among youth populations. It demonstrates that the impact of DHIs on HIV prevention varies substantially by intervention modality and outcome type. While TCI demonstrates the most consistent improvement in condom use at last sex, SWI and IOI may be more effective for promoting consistent condom use, though estimates remain imprecise. However, wide prediction intervals and low-certainty evidence suggest that self-reported behavioral changes may not translate into reductions in HIV/STI incidents without integration with offline services and broader structural support. Future trials might consider including standardized outcome indicators and longer follow-up to generate more precise estimates of the effectiveness of DHIs and guide generalization of youth-centered digital HIV/STIs prevention.

## Introduction

Adolescents and young adults aged 15‐24 years, defined as “youth” by the United Nations [1-3], are disproportionately affected by HIV around the globe. This age range is widely used in international health research and reporting, which allows comparability across studies and alignment with global HIV surveillance data. Alarmingly, in 2023, youth in this age group comprised nearly one-third of the 3600 daily new HIV infections recorded worldwide. Youth are especially vulnerable to HIV due to high rates of unprotected sex, inconsistent condom use, and co-occurring risk behaviors such as alcohol and drug use [4,5]. Still, a significant proportion of youth around the world lack access to accurate and age-appropriate information on sexual and reproductive health, rendering them susceptible to misinformation, psychological distress, and engagement in high-risk sexual behaviors [6]. To address this public crisis, scalable evidence-based health interventions targeting safer sex practices should be prioritized in this vulnerable population [7].

Digital health interventions (DHIs) have emerged as a promising strategy for health promotion in recent years [8]. Digital health, conceptualized as an umbrella term by the World Health Organization [9], refers to the use of digital and wireless platforms to facilitate health care delivery or health interventions, including but not limited to electronic health, mobile health, telehealth, and artificial intelligence-based applications. On the other hand, with the growing accessibility of smartphones and internet services among youth, digital technologies have become a dominant force to shape their sexual behaviors [10,11]. It is more convenient for young people to meet sexual partners, including casual, one-night, and anonymous partners, through web-based platforms, dating apps, and social networking sites [12,13], which further increases the likelihood of having unprotected sex frequency [14]. While digital technologies have facilitated riskier sexual behaviors among youth, they also create opportunities for DHIs that leverage young people’s existing online engagement patterns and preferences [15-19].

Accumulating evidence suggests that DHIs can improve HIV-related knowledge, risk perception, prevention intentions, and behavioral outcomes among youth [20], with the types of DHIs including mobile apps, text messaging, online videos, social media platforms, and interactive websites [21-23]. A stage-based computer-delivered intervention, for example, targeting heterosexual young men demonstrated significant improvements in condom use intention and subsequent condom use behavior [24]. Similarly, a study evaluating a social media-based intervention via Facebook reported a 23% increase in condom use and a 54% reduction in chlamydia incidence among adolescents [25]. In contrast, a large (randomized controlled trial (RCT) delivering sexual health promotion via SMS and email enhanced sexually transmitted infection (STI) knowledge and testing uptake, particularly among women, but showed no significant impact on condom use [26]. Another study reported that intervention based on a peer-led safer-sex Facebook group for Chinese college students found no significant change in contraceptive use intention or frequency [27]. Similarly, a social media-based crowdsourced HIV testing intervention among youth did not increase facility-based HIV testing, condom use, or syphilis testing [28]. Therefore, different types of DHIs may differentially affect sexual health outcomes, yet existing trials rarely distinguish the relative effectiveness of each DHI modality. Clarifying which intervention types are most effective for specific behavioral and biological outcomes is essential for optimizing digital HIV prevention strategies among youth [7,29].

In addition, several systematic reviews (SRs) have synthesized the evidence on DHIs for HIV prevention, but important limitations remain. Some SRs are purely descriptive, lacking quantitative synthesis [22,30-32]. Other reviews have focused narrowly on specific DHI types (eg, social media or telehealth) [33,34], or have failed to examine key behavioral outcomes like condom use [35,36]. In addition, traditional meta-analyses are constrained to pairwise comparisons [37], leaving uncertainty about which types of DHIs are most effective in head-to-head comparisons [30]. To address these knowledge gaps, we conducted a SR and network meta-analysis (NMA) to evaluate and compare the effectiveness of different DHIs in promoting safer sex behaviors among youth. The study aimed to: (1) identify the most effective types of DHIs in promoting safe sex among youth; (2) construct a network-based ranking of intervention effectiveness; and (3) inform the design of scalable, evidence-based digital health programs for HIV prevention among youth.

## Methods

### Overview

This SR and NMA follow the Preferred Reporting Items for Systematic reviews and Meta-Analyses (PRISMA-NMA) guidelines [38]. The completed PRISMA-NMA checklist is provided in Checklist 1. The protocol for this study has been registered with PROSPERO (CRD42024527317).

### Eligibility Criteria

#### Types of Population

Studies were eligible for inclusion if they involved participants aged 15‐24 years or if at least half of the participants were within this age range. Those that did not report, or for which data could not be extracted, for this specific age group were excluded.

#### Types of Interventions and Comparison

The interventions included in this review were DHIs, which were defined in accordance with the World Health Organization’s broad definition of digital health technologies [9]. For the purpose of this review, the included DHIs were further classified into four mutually exclusive categories based on their delivery modes and characteristics: (1) mobile app-based interventions (MAIs), (2) telecommunication-based interventions (TCI), (3) static web-based interventions (SWIs), and (4) interactive online-based interventions. This operational classification was developed to reflect the interventions identified in the included studies and to avoid overlap across categories. A detailed description of common DHI subcategories was provided in Table 1; the subcategories of the DHIs were based on a previous SR [30]. The control group received nondigital interventions (NDI), referring to traditional approaches without digital technology, such as face-to-face counseling, printed materials, or group sessions.

**Table 1.** Subcategories of digital health interventions (DHIs) and abbreviations used to classify interventions evaluated.

Subcategories of DHIs	Abbreviation	Description
Mobile app–based interventions	MAI	Programs delivered primarily via dedicated software apps installed on smartphones or tablets, leveraging device features (eg, notifications, sensors, data storage) to provide interactive content, personalized feedback, tracking, and behavior change support.
Telecommunication-based intervention	TCI	Interventions using traditional telecommunication methods such as SMS text messages or telephone calls. These interventions typically involve sending reminders, educational messages, or conducting counseling via phone communication without the need for internet-based platforms.
Static web-based interventions	SWI	Interventions provided through websites that offer static, noninteractive content. This may include informational pages, downloadable resources, or educational materials without features for user engagement or real-time feedback.
Interactive online-based interventions	IOI	Interventions delivered via web-based platforms or websites that enable user interaction, such as quizzes, tailored feedback, chatbots, or real-time communication with health professionals. These platforms actively engage users to enhance learning and behavior change.

Any appropriate comparator group was included, such as usual care, placebo, no intervention, waitlist, attention control, or different DHIs. To reduce inconsistency among trials, we excluded studies that combined non-DHIs with DHIs, unless the distinction between the intervention and control groups lay solely in the DHIs. In multi-arm trials, intervention arms representing the same modality without meaningful differences in content, intensity, or delivery were considered a single treatment node for eligibility purposes and later combined analytically to avoid double-counting.

#### Outcomes

The primary outcomes were specific condom use behaviors, defined as follows:

1. Condom Use Rate in the Last Sexual Contact: The percentage of individuals reporting condom use during their most recent penetrative sexual act [39].
2. Consistent Condom Use Rate: The percentage of individuals reporting consistent condom use during all their penetrative sexual acts over the recall period specified in each study [40].
3. Proportion of Condom Use: The overall proportion of sexual acts in which a condom was used, calculated as the total number of times a condom was used divided by the total number of sexual acts. Unlike the consistent condom use rate, which measures whether individuals always use a condom, this indicator captures the frequency of condom use across all reported sexual encounters, allowing for partial or occasional use [41].

The secondary outcomes included (1) self-efficacy for condom use, measured by the overall mean score on a validated condom use self-efficacy scale, such as Brafford and Beck’s [42] condom use self-efficacy scale, Lawrance et al’s [43] self-efficacy for HIV prevention scale and others, (2) number of sexual partners, and (3) the incidence rate of STIs (including HIV). Because follow-up length varied substantially across trials, we included studies reporting at least one postintervention follow-up outcome and extracted the longest follow-up time point for synthesis to enhance comparability [44,45].

#### Types of Studies

Only RCTs were included, including crossover trials and cluster-randomized trials. Studies using nonrandomized, quasi-experimental, observational, or qualitative designs were excluded. Only peer-reviewed articles published in English were eligible, as non-English or non-peer-reviewed sources lack sufficient methodological detail for reliable data extraction and risk-of-bias assessment.

### Search Strategy

The search strategy was developed and reported in accordance with the PRISMA-S guideline [46]. Searches for RCTs were conducted in PubMed (including MEDLINE), EMBASE, Web of Science, and the Cochrane Library. Searches were performed through the native interfaces of each database (PubMed via NCBI, EMBASE via Elsevier, Web of Science via Clarivate, and Cochrane Library via Wiley). In addition, the reference lists of relevant SRs were checked to ensure that no eligible trials were missed.

The search terms were formulated according to the PICOs framework, including participants or populations, interventions, outcomes, and types of research design. Both Medical Subject Headings and free-text terms were included as appropriate. Boolean operators (“AND,” “OR”) were used to combine search terms, and database-specific search techniques such as truncation, phrase marks, and wildcards were applied. The complete search terms and algorithm were provided in Multimedia Appendix 1, and search strategies for the other databases were adapted accordingly. The search was designed and executed by 2 reviewers (YZ and WP), who were trained in SR methodology, and the strategy was cross-checked for completeness and accuracy.

The literature search was initially conducted on June 13, 2024 and was last updated on November 15, 2025. All retrieved records were imported into EndNote X9 for citation management, and duplicates were removed using both automated and manual deduplication. Additional search methods included checking the reference lists of the included studies or relevant SRs. Gray literature was also searched via Google Scholar, OpenGrey, and ProQuest Dissertations. The search was limited to studies published in English due to resource constraints for translation.

### Selection Procedure and Data Extraction

Two reviewers (YZ and DH) independently screened the titles and abstracts against predefined protocol criteria. Full texts were retrieved for all potentially eligible studies. When multiple articles were identified from the same randomized controlled trial, the most recent or most comprehensive publication was retained for data extraction. Earlier reports were used to supplement missing information on study design, intervention details, or outcomes when necessary. Any discrepancies between the 2 reviewers were resolved by discussion. If disagreements persisted, a third reviewer (WP) was invited for adjudication. At the title and abstract screening stage, we excluded 14,788 records that clearly did not meet the eligibility criteria, most commonly because of wrong study design (eg, cross-sectional surveys, qualitative studies, reviews, protocols), wrong population (non-youth samples), ineligible intervention or comparator (ie, the difference between study arms did not lie in the use of a DHI), or an unrelated topic.

Data were extracted using a standardized and piloted form. Extracted variables included: first author, publication year, recruitment region, participant characteristics (mean age, SD, gender distribution), type of intervention and comparator, sample size per arm, intervention duration, and reported endpoints. Detailed characteristics of the DHIs were also extracted to facilitate subcategorization (Table 1). Further, outcomes and corresponding measurement methods were recorded, such as self-reported condom use and validated self-efficacy scales.

When outcome data were incomplete or unclear, study authors were contacted by email for clarification; trials with essential missing data were excluded from the quantitative synthesis and documented in Multimedia Appendix 2. All eligible studies were included in the SR, and only studies with usable and connected outcome data were included in the NMA.

### Risk of Bias Assessment

**Figure 1.**
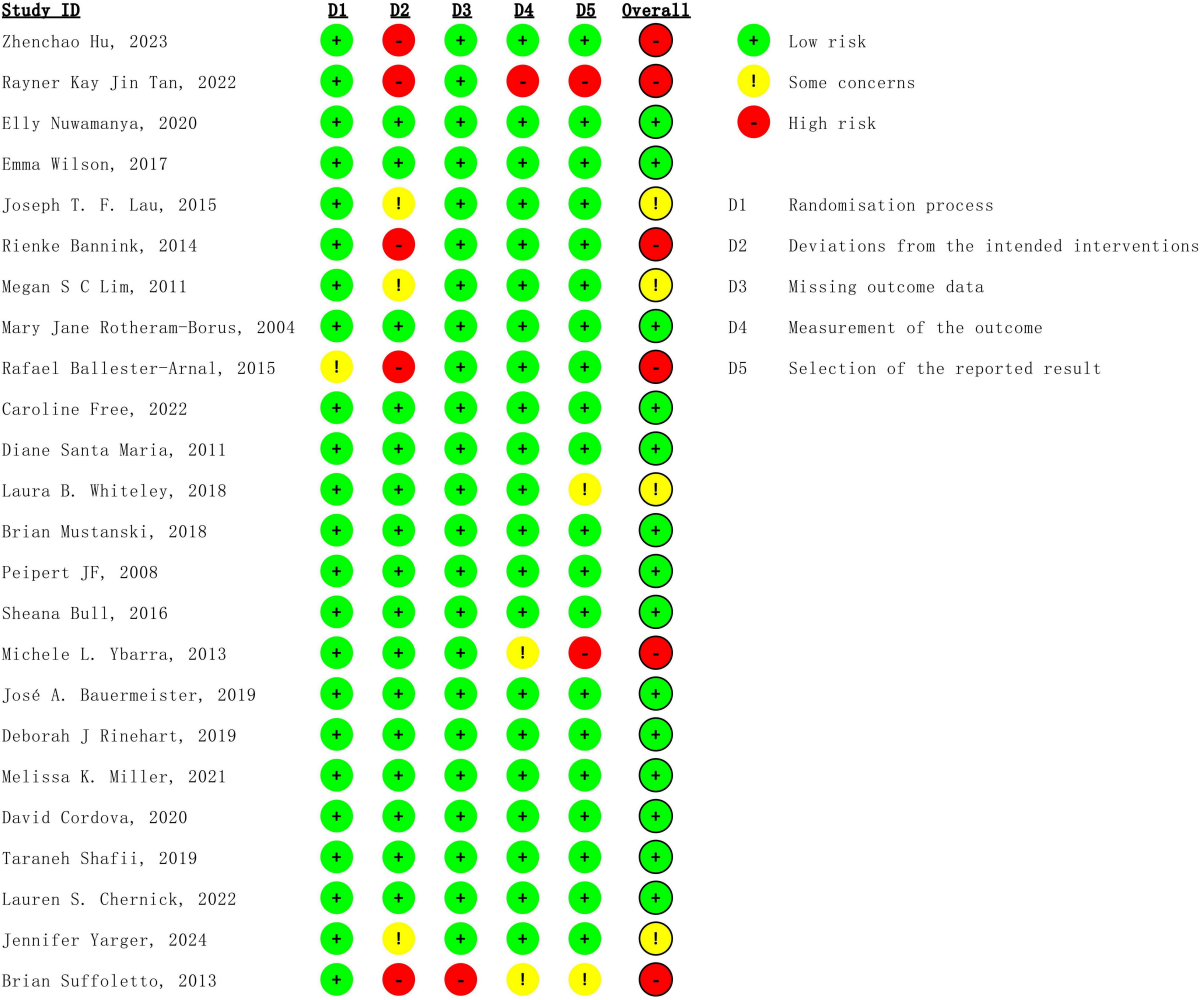
Risk of bias assessment of included randomized controlled trials using Cochrane Risk of Bias 2 tool [26,49-71].

The methodological quality of the included studies was independently assessed by 2 reviewers (YZ and WP), with disagreements resolved by a third reviewer (CZ). Risk of bias was evaluated using version 2 of the Cochrane risk of bias 2 tool (RoB 2) for randomized trials [47]. For each domain, studies were rated as having “low risk,” “some concerns,” or “high risk” of bias according to the Cochrane Handbook (version 6.5) [48]. Domain-level risk of bias judgments for each trial are summarized in Figure 1, with extended graphs and contribution matrices provided in Multimedia Appendix 3. The reference list of included studies is provided in Multimedia Appendix 4 [26,49-71].

Because blinding was generally not feasible for these nonpharmacological interventions, many trials were judged at high risk of bias in the domain of deviations from intended interventions [72,73]. As this limitation was expected and unlikely to influence objectively measured outcomes, we did not consider this domain when grading the certainty of evidence. The overall certainty of evidence was determined using the CINeMA (Confidence in NMA) web application, which is based on the GRADE framework [74,75]. In addition, we constructed GRADE “Summary of Findings” tables using the official template provided by the GRADE Working Group to summarize the key relative and absolute effects and certainty ratings for the primary outcomes (Multimedia Appendix 3).

RoB 2 assessments were incorporated into the interpretation of NMA findings and into the GRADE/CINeMA evaluation of certainty, but they were not used to weight studies in the statistical synthesis.

### Statistical Analysis

#### Geometry of the Evidence Network

We examined the geometry of the treatment network by mapping each trial arm to one of the predefined intervention nodes and summarizing the pattern of direct comparisons. A network plot was generated to visually depict the evidence base, with node size proportional to the number of randomized participants and edge thickness reflecting the number of trials informing each comparison. We further assessed potential network-related biases by identifying sparse nodes, single-study comparisons, and imbalance in the distribution of direct evidence.

#### Model Specification and Synthesis Methods

Model convergence was assessed through Markov Chain Monte Carlo diagnostics, including the Gelman-Rubin potential scale reduction factor and inspection of leverage plots. The number of adaptation iterations, burn-in period, and total iterations were set to ensure adequate mixing and convergence. Effect estimates were expressed as pooled odds ratios (ORs) and 95% credible intervals (CrIs), which served as the primary summary measure for all dichotomous outcomes.

Bayesian NMAs were conducted using the R package *BUGSnet* to compare the effectiveness of 4 subcategories of DHIs and control groups. Binomial likelihood models with a logit link function were specified, and both fixed-effect and random-effects consistency models were fitted. Given anticipated clinical heterogeneity, random-effects models were treated as primary, with fixed-effect models used in sensitivity analyses. Noninformative priors were assigned to treatment effects and heterogeneity parameters to minimize prior influence. Model fit and parsimony were evaluated using the deviance information criterion (DIC), with lower values indicating better fit.

To evaluate the transitivity assumption, we compared mean age, sex distribution, intervention intensity, and follow-up duration across treatment comparisons. We further restricted inclusion to trials in which ≥50% of participants were aged 15‐24 years, excluded trials in which nondigital components were offered only to one arm, and extracted outcomes at the longest reported follow-up to harmonize follow-up time. These design and population characteristics showed broadly overlapping ranges across interventions and no systematic differences between comparisons, so transitivity was judged plausible. Forest plots of posterior ORs with 95% CrIs from the Bayesian consistency model were generated to summarize the magnitude and uncertainty of estimated treatment effects.

To complement these Bayesian estimates and quantify uncertainty in effects that might be observed in new settings, we also performed frequentist random-effects NMAs using the netmeta package in R, specifying NDI as the reference group [76]. For each outcome, we estimated ORs and 95% CIs for each intervention versus NDI and derived 95% prediction intervals (PIs) by combining the average treatment effect with between-study heterogeneity, in line with recent recommendations that NMAs should routinely report PIs when heterogeneity is present [77].

#### Assessment of Inconsistency and Heterogeneity

Consistency between direct and indirect evidence was assessed by comparing the DIC between consistency and inconsistency models. A substantially lower DIC in the consistency model indicated acceptable agreement between sources of evidence. Due to the limited number of included studies, we did not formally investigate small-study effects (eg, using funnel plots or Egger’s regression test), which are typically used to explore potential publication bias as one of several possible explanations for such effects. Selective outcome reporting could not be formally assessed due to insufficient reporting in the included trials; however, the potential for selective reporting was considered when interpreting the cumulative evidence. Because the number of studies informing most comparisons was limited, local inconsistency (eg, node-splitting) could not be reliably assessed; in the presence of any potential inconsistency, we planned to explore differences in study characteristics and reassess the plausibility of the transitivity assumption.

#### Handling of Multi-Arm Trials and Node Merging

For multi-arm trials, if 2 or more arms delivered essentially the same intervention category (eg, different versions of the same SWI content without meaningful variation in delivery or timing), we merged these arms by summing the number of events and participants. This ensured that each intervention was represented by a single node in the network and avoided duplicate contributions from the same trial [78].

#### Ranking of Interventions

Ranking probabilities and surface under the cumulative ranking curve (SUCRA) were computed to summarize the relative effectiveness of each intervention across the posterior distribution. Rankograms and cumulative ranking plots were used to visualize intervention hierarchies, and league tables and heatmaps were generated to present pairwise comparisons and their relative effect estimates. No additional analyses, such as sensitivity analyses, subgroup analyses, or meta-regression, were conducted because the limited number of studies and the sparse network geometry did not permit reliable implementation of these methods. All statistical analyses were conducted using R (version 4.3.2; R Foundation for Statistical Computing) with the gemtc, BUGSnet, and netmeta packages.

## Results

### Description of Included Studies

**Figure 2.**
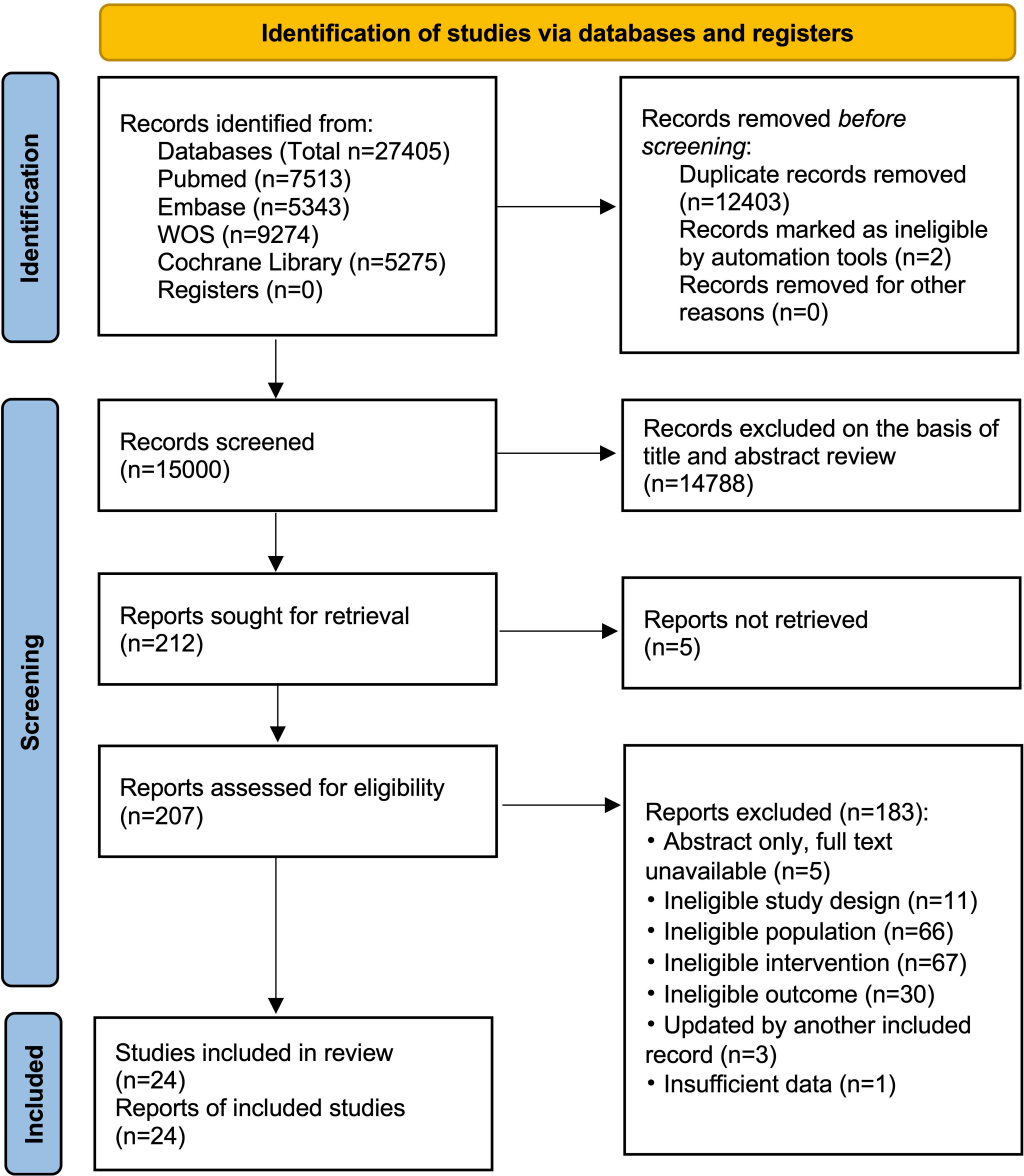
The flow diagram of the literatureease clarify th selection process for randomized controlled trials of digital health interventions included in this review.

From a total of 25,659 records initially retrieved, 24 RCTs published between 2004 and 2024 were included in the final analysis (Figure 2). These studies were conducted across 8 diverse countries, predominantly in the United States (n=14), with the remainder from China (n=2), the United Kingdom (n=2), Uganda (n=2), Singapore (n=1), the Netherlands (n=1), Australia (n=1), and Spain (n=1). The trials collectively enrolled 20,134 participants (range 50‐6248; mean 838.9, SD 1358.2), with a mean age of 19.5 years. Overall, 10,228 participants (53.4%) were male, although sex composition varied substantially—some studies enrolled only males [49-51], only females [52,53], or mixed populations. Of the 24 included studies, 21 studies were two-arm, and 3 studies were multi-arm. Across the included studies, 6 trials evaluated TCI, 8 assessed interactive online-based intervention (IOI), 6 examined MAI, and 8 investigated SWI. The total number of intervention approaches (n=28) exceeded the number of included studies (n=24) because several trials directly compared 2 or more active interventions (eg, IOI vs SWI) without including a conventional control group. Intervention durations ranged from brief sessions lasting 10‐20 minutes up to 12 months. Follow-up periods were heterogeneous, spanning from immediate postintervention assessments to 24 months. Most studies reported outcomes at 3‐6 months, while only a few provided longer-term follow-up beyond 12 months. Five studies performed analyses for more than one time point. To enhance consistency and reduce potential bias associated with short-term variability, we extracted outcomes at the longest follow-up time point, thereby facilitating a more comprehensive evaluation of the intervention’s long-term effectiveness [44,45].

For condom use at last sexual contact, 4 out of 7 studies reported ORs greater than 1, suggesting a possible beneficial effect of the interventions; however, only one trial [54] showed a clear statistical significance (OR 1.13, 95% CI 1.01‐1.25). Regarding consistent condom use, 6 of 11 studies showed ORs above 1, with substantial heterogeneity; one study reported a very large effect (OR 4.55, 95% CI 1.15‐17.95) [55]. For the proportion of condom use, 5 out of 6 trials reported ORs above 1, suggesting a tendency towards higher condom use in the intervention groups; however, CIs were wide and often included the null (overall OR range 0.48‐2.43), indicating that the evidence for this outcome is imprecise. Finally, for STIs incidence, including HIV, effects varied substantially across 7 trials, with ORs ranging from 0.53 to 2.10. Four of the 7 trials had point estimates below 1 [49,50,53,79], and 3 trials had 95% CI that excluded 1 [49,56,57], indicating heterogeneous and partly conflicting evidence for this outcome. These results summarized the observed effects across trials, highlighting that effect estimates varied considerably across outcomes and studies. The characteristics and effect estimates of the included studies were summarized in Table 2.

**Table 2.** Study characteristics and effect estimates of the included trials.

Study (author, year)	Region / country	Study design	Age (years)		Males, n (%)	Intervention name	Theoretical framework	Delivery mode or digital tools	Intervention / comparator	Sample size (intervention / comparator)	Intervention dosage / Intervention duration	Follow-up	Endpoints	Effect estimates (OR, 95% CI)
			Mean (SD)	Median										
Hu et al [57]	China	RCT^a^	16.09 (0.84)	—^b^	1691 (53.67)	“You and Me	—	Online education sessions; Internet-based educational platform; cartoon videos; PowerPoint slides	IOI^c^ / NDI^d^	1760/1391	45 min sessions/8 wk	End of intervention / 12 wk	①, ④	0.72 (0.31‐1.69) ① 1.42 (1.16‐1.74) ④
Tan et al [50]	Singapore	RCT	23.90 (2.98)	—	300 (100.00)	People Like Us (PLU) web drama video series	Predetermined theory of behavior change	Web-based	SWI / NDI	150/150	6 videos, each about 10 min in length/1 wk	6 mo	②, ④	1.22 (0.59‐2.51) ② 0.82 (0.40‐1.70) ④
Nuwamanya et al [58]	Uganda	RCT	21.00 (2.00)	—	407 (36.60)	MPA-SRH (mobile phone apps-sexual reproductive health）	—	Mobile app	MAI / NDI	556/556	Within 6 mo, open for use to the intervention group.	End of intervention	①	1.23 (0.90‐1.68)
Wilson et al [56]	The United Kingdom	RCT	23.00 (3.55)	—	846 (41.01)	SH:24 website	—	Website, online service	SWI^e^ / NDI	1031/1032	All participants were free to use any other sexual health services or interventions during the trial period/Median =28.8 d (participant-dependent duration)	6 wk	④	2.10 (1.11‐4.01)
Lau et al [51]	China	RCT	—	—	396 (100.00)	Online intervention based on STD-related cognitions involving videos (SC)/online intervention based on both STD-related cognitions and emotions (eg, fear) involving fear-arousing imagery and videos (SCFI)	—	Online videos (Sent Intervention package email）	SWI / NDI	261/135	10‐20 min	3 mo	③	1.10 (0.61‐1.97)
Bannink et al [59]	Netherlands	RCT	15.81 (0.68)	—	446 (54.00)	The E-health4Uth Intervention	—	Web-based tailored messages	IOI / NDI	392/434	1 mo	4 mo	②	1.59 (0.94‐2.70)
Lim et al [26]	Australia	RCT	—	19	417 (41.99)	Email and SMS to a group of young people (intervention gro	—	SMS, email	TCI^f^ / NDI	507/486	SMS messages were sent every 3‐4 wk (a total of 14) emails were sent less than monthly (a total of 8)/12 mo	end of intervention (6 mo) / end of intervention (12 mo)	②	0.75 (0.46‐1.24)
Rotheram-Borus et al [60]	The United States	RCT	—	23	136 (77.71)	Telephone intervention	—	Telephone sessions	TCI / NDI	59/116	2 h/6 wk	15 mo	②	0.76 (0.40‐1.42)
Ballester-Arnal et al [61]	Spain	RCT	20.90 (1.90)	—	60 (25.00)	Fear induction group/website group	Information-Motivation-Behavioral Skills (IMB) Model	Video, music/website; computer based	SWI / NDI	Estimated 34‐35 participants per group (total n=239; balanced allocation across 7 groups)	1 h	1 months / 4 mo	③	1.54 (0.43‐5.44)
Free et al [54]	The United Kingdom	Parallel group RCT	20.35 (2.1)	—	2162 (34.60)	text messaging intervention (safetxt)	COM-B (capability, opportunity, motivation, and behavior) model	Delivered by text messages to improve safer sex behaviors	TCI / NDI	3123/3125	Day 1‐3: 4 items per day Day 4‐28: 1‐2 items per day Second month: 2‐3 items per week Month 3‐12: 2‐5 items per month/12 mo	4 weeks / 12 mo	①, ④	1.13 (1.01‐1.25) ① 1.11 (0.98‐1.25) ④
Santa Maria et al [62]	The United States	Pilot RCT	21.20 (2.10)	—	56 (52.34)	MY-RID (Motivating Youth to Reduce Infection and Disconnection)	Information-Motivation-Behavioral Skills (IMB) Model	Smartphone-based Just-in-Time Adaptive Intervention (JITAI) Android smartphones with unlimited data were provided to participants	MAI^g^ / NDI	48/49	Participants will receive a customized message for each EMA assessment completed.• Weeks 1‐2: 3 times per day• Weeks 3‐4: 2 times per day• Weeks 5‐6: 1 time per day• Each EMA took 1‐5 min to complete/6 wk	end of intervention	②	2.00 (0.75‐5.33)
Whiteley et al [55]	The United States	Pilot RCT	18.60 (2.30)	—	37 (61.67)	Online HIV/STI^h^ prevention intervention	Information-Motivation-Behavioral Skills (IMB) Model	Publicly available websites and YouTube videos related to HIV/STI prevention Content accessible via computer, smartphone, or tablet	SWI / NDI	31/29	Twice per week for 4 wk, each contained 2‐3 website or video links Total exposure: 8 emails with up to 19 different resources/4 wk	3 mo	②	4.55 (1.15‐17.95)
Mustanski et al [49]	The United States	RCT	23.82^i^	—	901 (100.00)	“Keep It Up!”	Information-Motivation-Behavioral Skills (IMB) model	Fully online (eHealth intervention), delivered via computers and tablets (not available on mobile phones).	IOI / SWI	445/456	Each session lasted about 1 h./≥3 d for core intervention (3 sessions ≥24 h apart); booster sessions at 3 and 6 mo	12 mo	③, ④	1.34 (1.00‐1.80) ③ 0.56 (0.35‐0.89) ④
Peipert et al [53]	The United States	RCT	—	22	0 (0)	Project PROTECT	Transtheoretical (TTM) model of behavior change	Computer-based multimedia program	IOI / SWI	272/270	Three computer-based sessions /80 d	24 mo	②, ④	0.99 (0.70‐1.38) ② 0.96 (0.61‐1.53) ④
Bull et al [63]	The United States	RCT	14.94 (1.08)	—	415 (48.71)	Teen Outreach Program (TOP)+ text message program: Youth All Engaged (YAE!)	Integrated Theory of mHealth	Text messages based on social media	TCI / NDI	436/416	5 and 7 messages weekly/25 wk	end of intervention	③	1.13 (0.36‐3.53)
Ybarra et al [64]	Uganda	RCT	16.10 (1.40)	—	307 (83.88)	CyberSenga	Information-Motivation-Behavioral Skills (IMB) model	Website based; computer-based	IOI / NDI	183/183	One module per week, a total of 5 modules need to be completed./5 wk	3 mo	②	0.91 (0.50‐1.66)
Bauermeister et al [65]	The United States	Pilot RCT	21.67 (1.81)	—	123 (100.00)	myDEx(My Desires & Expectations)	The dual processing cognitive-emotional decision-making framework	Online-delivered HIV prevention interventions; App	MAI / SWI	95/28	myDEx intervention includes 6 personalized online courses, completed within 3 mo, with participants logging in an average of about 5 times, and a total conversation volume of about 7 times/3 mo	end of intervention	③	2.43 (1.01‐5.80)
Rinehart et al [66]	The United States	Pilot RCT	15.90 (1.60)	—	0 (0)	Texts for Sexual Health Education and Empowerment (t4she)	Health belief model	Text message	TCI / NDI	122/122	58 automated messages sent over 12 wk/12 wk	3 months / 6 mo	①	1.40 (0.66‐2.96)
Miller et al [67]	The United States	Pilot RCT	16.90 (1.00)	—	26 (28.57)	SexHealth	Theory of Planned Behavior to inform intervention content and the Social Ecological Model	A tablet-based, interactive intervention: The educator used a tablet to deliver the intervention, intermittently sharing the screen with the participant.	IOI / NDI	44/47	25 min	6 mo	①	0.50 (0.10‐2.60)
Cordova et al [68]	The United States	Pilot RCT	18.82 (2.1)	—	4 (8.00)	Storytelling 4 Empowerment (S4E)	An ecodevelopment and empowerment framework	Multilevel Mobile Health App	MAI / NDI	25/25	Complete 3 interactive modules at once /30‐45 min	1 mo	③	0.48 (0.04‐5.68)
Shafii et al [69]	The United States	Pilot RCT	21^i^	—	176 (64.71)	e-KISS	Information-Motivation-Behavioral Skills (IMB) model	An interactive computer-based intervention; video	IOI / NDI	130/142	15‐20 min, a single, one-time interactive /15‐20 min	2 mo	④	0.53 (0.26‐1.08)
Chernick et al [52]	The United States	Pilot RCT	17.74 (1.27)	—	0 (0)	Dr. Erica (Emergency Room Interventions to improve the Care of Adolescents)	Intervention mapping, a program-planning framework; the Social Cognitive Theory and Motivational Interviewing	Multimedia text messaging; video; mobile-based	IOI / NDI	72/74	A minimum of 56 and maximum of 121 texts, with additional texts sent based on keywords./3 mo	3 mo	①, ②	0.95 (0.41‐2.21) ① 1.53 (0.55‐4.25) ②
Yarger et al [70]	The United States	A Cluster Randomized Trial	15.7^i^	—	340 (40.72)	In the Know——an in-person, group-based sexual health education program integrating digital technologies,	—	Technology-based; mobile-based; app	MAI / NDI	348/487	1.5 h/4 wk	3 mo	②	0.86 (0.58‐1.27)
Suffoletto et al [71]	The United States	Pilot RCT	21.44 (2.04)	—	0 (0)	SMS program	The Health Belief Model; the Information Motivation Behavior model	Text message	TCI / NDI	23/29	Once a week, send a text message at noon every Sunday/12 wk	3 mo	①, ②	1.86 (0.48‐7.12) ① 1.60 (0.37‐6.96) ②

a RCT: randomized controlled trial.

b Not available.

c IOI: interactive online-based intervention.

d NDI: nondigital intervention.

e SWI: static web-based intervention.

f TCI: telecommunication-based intervention.

g MAI: mobile app-based intervention.

h STI: sexually transmitted infection.

i The included studies did not report SD values for these mean estimates, and the SDs cannot be derived from the available information.

Risk-of-bias assessments using the RoB 2 tool are summarized in Figure 1. Overall, most trials were judged to be at low risk of bias for the randomization process, outcome measurement, and selection of the reported result. However, a substantial minority of studies had some concerns or high risk of bias in at least one domain, most frequently for deviations from the intended interventions and missing outcome data. Consequently, several trials were rated as having some concerns or a high overall risk of bias.

Although condom use self-efficacy and number of sexual partners were prespecified as secondary outcomes, too few studies reported these measures to allow meta-analysis. Only one trial evaluated condom use self-efficacy. In Rinehart et al [66], this construct was assessed using 3 items developed within the Health Belief Model (range 0‐12; Cronbach *α*=0.72). At the 3rd month, the intervention group reported significantly higher self-efficacy scores than the control group (7.38 vs 6.68; *P*=.04), but this difference was no longer significant at the 6th month (7.39 vs 6.99; *P*=.20). Two trials reported on the number of sexual partners. In Shafii et al [79], participants in the intervention arm reported a 29% reduction in the number of sexual partners at follow-up, although the effect did not reach statistical significance (IRR 0.71, 95% CI 0.50‐1.03, *P*=.07). Changes in the control group were not reported. By contrast, Free et al [54] examined the proportion of participants reporting 2 or more sexual partners over 12 months. At one year, this outcome was reported by 56.9% of intervention participants compared with 54.8% of controls (OR 1.11, 95% CI 1.00‐1.24, *P*=.06). Overall, the evidence on the impact of digital interventions on the number of sexual partners remains limited and inconsistent.

### Results of Network Meta-Analysis

#### Overview

**Figure 3.**
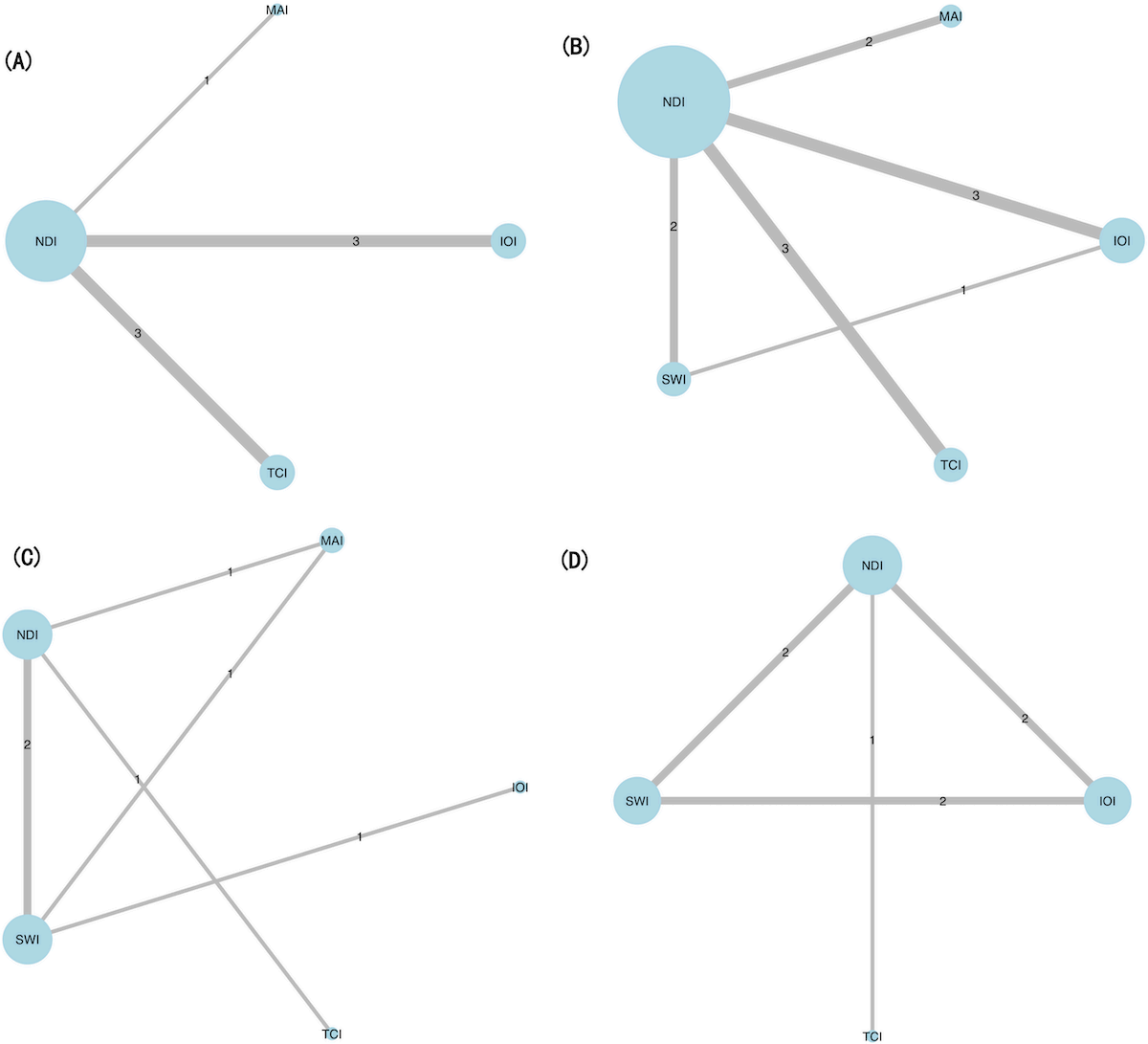
Network structure diagrams for randomized controlled trials of digital health interventions among youth, by outcome: (**A**) Condom use rate in the last sexual contact; (**B**) Consistent condom use rate; (**C**) Proportion of condom use; (**D**) The incidence rate of sexually transmitted infections (including HIV). The thicknesses of the lines were proportional to the number of comparisons; the diameters of the circles were proportional to the number of treatments. IOI: interactive online-based intervention; MAI: mobile app-based intervention; NDI: nondigital intervention; SWI: static web-based intervention; TCI: telecommunication-based intervention.

A total of 24 RCTs were included to evaluate the comparative effectiveness among 5 intervention types—4 DHIs (TCI, IOI, MAI, and SWI) and NDI—across the four analyzable outcomes: (1) condom use at last sexual contact, (2) consistent condom use, (3) overall proportion of condom use, and (4) incidence of STIs (including HIV). The remaining 2 outcomes of self-efficacy were excluded due to insufficient network connectivity. The network structures for each outcome were shown in Figure 3, where the thickness of the lines was proportional to the number of comparisons, and the size of the nodes reflected the number of studies involving each intervention. Across outcomes, the treatment network was dominated by comparisons of each DHI category versus NDI, whereas head-to-head trials comparing different DHIs were rare. Several DHI-DHI contrasts and some STI outcomes were informed by only one or two small trials, and self-efficacy outcomes formed disconnected subnetworks. Thus, the network geometry was relatively sparse and heavily anchored on NDI, implying that several treatment rankings rely mainly on indirect evidence. The indirect comparative effectiveness of DHIs was summarized in Multimedia Appendix 5. Forest plots of posterior ORs with 95% CrIs for each intervention versus NDI across all 4 outcomes are provided in Multimedia Appendix 6.

In the complementary frequentist random-effects NMAs, point estimates and 95% CIs for each intervention versus NDI were broadly consistent with the Bayesian results (Multimedia Appendix 7). Across all 4 outcomes, 95% PIs were noticeably wider than the corresponding CI*s* and frequently included the null value, even when the average effects suggested benefit. For example, for condom use at last sexual contact and for consistent condom use, TCI, IOI, and MAI tended to favor improved condom use versus NDI, but their PIs indicated that future trials conducted in different settings could plausibly observe smaller benefits or no clear difference from NDI. Similar patterns were observed for the proportion of condom-protected acts and for STI incidence, highlighting that between-study heterogeneity and contextual differences may lead to substantial variability in the effects realized in new populations.

#### Condom Use Rate in the Last Sexual Contact

**Figure 4.**
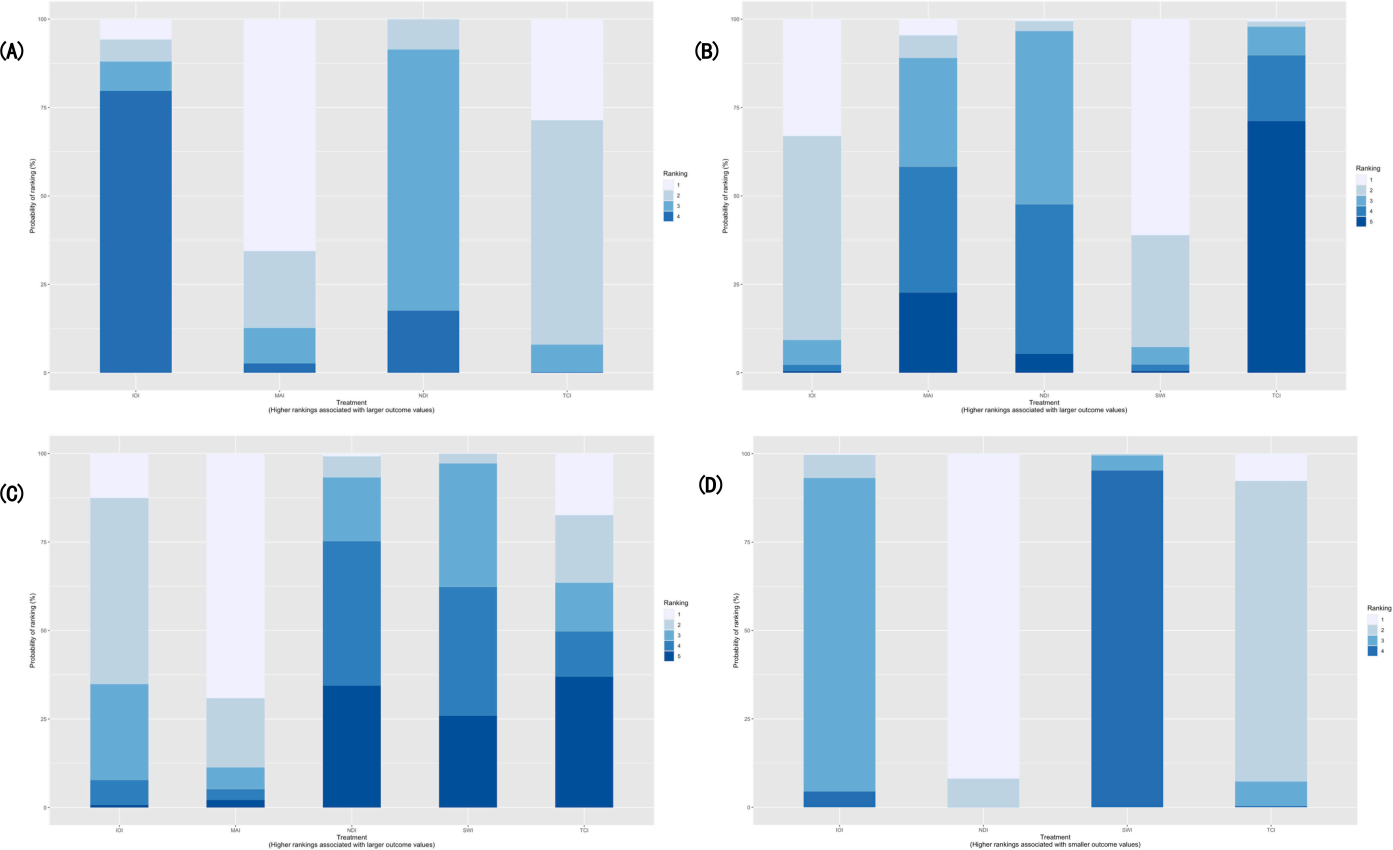
Rank of probabilities of digital health intervention categories in the network meta-analysis of randomized controlled trials assessing sexual health outcomes among youth: (**A**) Condom use rate in the last sexual contact; (**B**) Consistent condom use rate; (**C**) Proportion of condom use; (**D**) The incidence rate of sexually transmitted infections (including HIV). Stacked bars show the probability that each digital health intervention category occupies each possible rank (from best to worst).

Seven studies involving 4 DHIs with a total of 10,285 participants were included in the analysis of condom use at last sexual contact. The random-effects consistency model was selected based on model fit, as it showed comparable DIC and residual deviance values to the inconsistency model, indicating no substantial inconsistency. Among the interventions, only TCI showed a statistically significant improvement compared with NDI (OR 1.13, 95% CrI 1.02‐1.26). Although MAI had the highest SUCRA value (83.44%) and was most likely to rank first (65.61%), its effect was not statistically significant. The rank probabilities for all interventions were summarized in Table 3A and illustrated in Figures4  5, showing the descending order of MAI, TCI, NDI, and IOI. As shown in Multimedia Appendix 6, TCI was the only intervention with its 95% CrI entirely to the right of the line of no effect, suggesting a modest but relatively certain increase in condom use at last sex compared with NDI. IOI and MAI showed point estimates on either side of 1 with wide CrIs, indicating no clear difference from NDI.

**Table 3.** Rank probabilities and surface under the cumulative ranking curve (SUCRA) values for digital health intervention categories in the network meta-analysis of randomized controlled trials assessing sexual health outcomes among youth.

Rank	IOI	MAI	NDI	SWI	TCI
(A) rank probability of condom use rate in the last sexual contact					
1	5.76	65.61	0.05	—^a^	28.58
2	12.02	87.34	8.58	—	92.05
3	20.34	97.38	82.46	—	99.82
4	100	100	100	—	100
SUCRA	12.71	83.44	30.36	—	73.48
(B) Rank probability of consistent condom use rate					
1	33.07	4.59	0.54	61.09	0.7
2	90.77	10.95	3.35	92.76	2.16
3	97.78	41.8	52.43	97.71	10.27
4	99.59	77.38	94.65	99.5	28.87
5	100	99.99	99.99	100	100
SUCRA	80.3	33.68	37.74	87.77	10.5
(C) Rank probability of proportion of condom use					
1	12.6	69.18	0.8	0.04	17.39
2	65.17	88.68	6.82	2.82	36.53
3	92.3	94.87	24.8	37.75	50.31
4	99.3	97.89	65.6	74.13	63.11
5	100	100	100	100	100
SUCRA	67.34	87.66	24.5	28.68	41.84
(D) Rank probability of the incidence rate of STIs (including HIV)					
1	0.38	—	91.78	0.04	7.81
2	7	—	99.95	0.43	92.63
3	95.79	—	100	4.5	99.72
4	100	—	100	100	100
SUCRA	34.39	—	97.25	1.66	66.72

a Not available.

**Figure 5.**
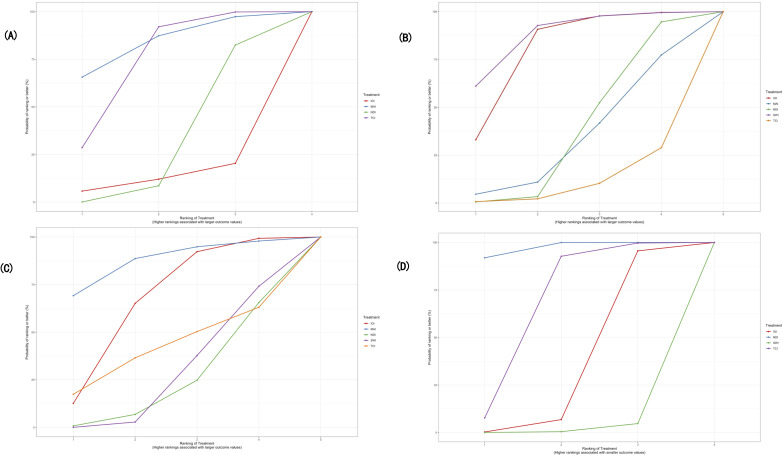
Cumulative rank plot for digital health intervention categories in the network meta-analysis of randomized controlled trials assessing sexual health outcomes among youth: (**A**) Condom use rate in the last sexual contact; (**B**) Consistent condom use rate; (**C**) Proportion of condom use; (**D**) The incidence rate of sexually transmitted infections (including HIV). Lines represent the cumulative probability of each digital health intervention category being ranked at or above each position.

#### Consistent Condom Use Rate

Eleven studies involving all 5 DHIs with a total of 4881 participants were included in the analysis of consistent condom use. A random-effects consistency model was selected based on a slightly better model fit (DIC=39.49 vs 41.17) and no substantial evidence of inconsistency. Both SWI and IOI were significantly more effective than TCI (SWI vs TCI: OR 1.77, 95% CrI 1.03‐3.06; IOI vs TCI: OR 1.68, 95% CrI 1.02‐2.76). SWI had the highest probability of being the most effective intervention (SUCRA=87.77%), followed by IOI (80.3%). TCI ranked the lowest (10.5%), while MAI and NDI showed moderate effectiveness. The ranking probabilities were summarized in Table 3B and visualized in Figures3B  4B. Multimedia Appendix 6 shows that IOI and SWI had posterior ORs above 1, implying a tendency toward improved consistent condom use, whereas MAI and TCI showed ORs close to or below 1. However, all CrIs crossed 1, suggesting that these differences were uncertain.

#### Proportion of Condom Use

Six studies involving 5 DHIs with a total of 2048 participants were included in the analysis of the proportion of condom use. Given the slightly lower DIC (22.45 vs 22.83) and similar model complexity and fit, the consistency model was deemed preferable. Only IOI showed a statistically significant improvement compared with SWI (OR 1.34, 95% CrI 1.01‐1.80). Regarding the ranking probabilities (Table 3C), MAI had the highest probability of being the most effective intervention (SUCRA=87.66%), followed by IOI (SUCRA=67.34%). In contrast, NDI (SUCRA=24.5%) and SWI (SUCRA=28.68%) ranked relatively low. The distribution of rank probabilities was presented in Table 3C and visualized in Figures3C  4C. In Appendix 4C, all interventions showed ORs >1 relative to NDI, with MAI and IOI having the largest point estimates. Nevertheless, the CrIs were wide and crossed 1, indicating that although the direction of effect generally favored digital interventions, the precision of the estimates was limited.

#### The Incidence Rate of STIs (Including HIV)

Seven studies involving 4 DHIs and a total of 14,966 participants were included in the analysis of STIs incidence. NDI was significantly more effective than IOI (OR 0.78, 95% CrI 0.65‐0.93) and SWI (OR 0.61, 95% CrI 0.46‐0.82), while TCI also showed a significant advantage over SWI (OR 0.67, 95% CrI 0.49‐0.92). Based on rank probabilities and SUCRA values, NDI had the highest likelihood of being the most effective intervention (91.78% probability of ranking first; SUCRA=97.25%), followed by TCI (7.81%; SUCRA=66.72%). IOI and SWI had considerably lower rankings, with SUCRA values of 34.39% and 1.66%, respectively. Table 3D summarizes the intervention rankings, and Figures3D  4D illustrate the rank probability and cumulative ranking plots. Multimedia Appendix 6 displays ORs >1 for IOI, SWI, and TCI compared with NDI, and all CrIs lie entirely to the right of 1. This pattern suggests that these digital interventions were associated with equal or higher STI incidence, with SWI showing the highest point estimate.

#### Consistency and Visualization

For all 4 outcomes, consistency between direct and indirect comparisons was assessed by comparing the DIC values between the consistency and inconsistency models. In all cases, the DIC differences were less than 5, indicating no evidence of global inconsistency (Multimedia Appendix 8). In complementary frequentist random-effects NMAs conducted with the netmeta package in R, we estimated ORs with 95% CIs and 95% PIs for each intervention versus NDI for all 4 outcomes (Multimedia Appendix 7). Across outcomes, PIs were wider than the corresponding CIs and frequently crossed the null, indicating substantial uncertainty in the effects that might be observed in future implementation settings despite the direction of the average effects.

### Strength of Evidence

All of these enrolled studies were RCTs, and the quality of evidence was evaluated by the Cochrane Handbook and graded each potential source of bias as low, high, or some concerns; the details were displayed in Figure 1. We assessed confidence in the results of the NMA using the CINeMA framework. Of the 4 outcomes analyzed, only “consistent condom use” met the criteria for CINeMA assessment. The remaining outcomes were excluded due to insufficient numbers of studies or disconnected network structures. For consistent condom use, certainty of evidence was mainly downgraded for within-study bias and imprecision, resulting in overall ratings of “low” to “very low” confidence. Among the 10 comparisons, 1 (10%) was rated as “very low” and 9 (90%) as “low” certainty. For condom use at last sex, the proportion of condom-protected acts, and STI incidence, the certainty of evidence is less well characterized, but given the sparse data, risk of bias, and wide intervals, these estimates should similarly be interpreted as low certainty. A detailed summary of risk-of-bias judgements, CINeMA assessments, and the corresponding GRADE “Summary of Findings” information for each primary outcome is provided in Figure 1 and Table 4, with full GRADE “Summary of Findings” tables formatted according to the GRADE Working Group template available in Multimedia Appendix 3 to aid interpretation of the magnitude and certainty of the main comparisons.

**Table 4.** Summary of confidence in the evidence for consistent condom use, assessed using Confidence in Network Meta-Analysis and GRADE.

Comparison	Number of studies	Within-study bias	Reporting bias	Indirectness	Imprecision	Heterogeneity	Incoherence	Overall confidence rating (GRADE)	Reasons for downgrading
IOI:NDI^a^^b^	3	Major concerns	Low risk	No concerns	Major concerns	No concerns	No concerns	Very low	Within‐study bias and imprecision
IOI:SWI^c^	1	No concerns	Low risk	No concerns	Major concerns	No concerns	No concerns	Low	Imprecision only
MAI:NDI^d^	2	Some concerns	Low risk	No concerns	Major concerns	No concerns	No concerns	Low	Within‐study bias and imprecision
NDI:SWI	2	Some concerns	Low risk	No concerns	Major concerns	No concerns	No concerns	Low	Within‐study bias and imprecision
NDI:TCI^e^	3	Some concerns	Low risk	No concerns	Major concerns	No concerns	No concerns	Low	Within‐study bias and imprecision

a IOI: interactive online-based intervention.

b NDI: nondigital intervention.

c SWI: static web-based intervention.

d MAI: mobile app-based intervention.

e TCI: telecommunication-based intervention.

## Discussion

### Principal Findings

To the best of our knowledge, this is the first NMA to systematically evaluate the comparative effectiveness of different modalities of DHIs on promoting safer sexual behaviors among youth. By simultaneously examining 4 distinct digital modalities and 3 behavioral outcomes, and by incorporating STI incidence (including HIV) as a biological endpoint, this review expands the current evidence base and clarifies which intervention types are better suited for immediate versus sustained behavior change, and highlights the gap between improvements in self-reported safer behaviors and reductions in biological HIV/STI infection. Drawing upon data from 24 RCTs across diverse contexts, our findings offer comprehensive insights to inform future development, optimization, and implementation of DHIs in HIV/STIs prevention, underscore the need for designing multimodal, context-aware digital interventions that integrate behavioral support with access to testing and care services, and provide practical considerations for policymakers, program designers, and digital platform developers who seek to tailor DHIs to youth populations.

Across outcomes, between-study heterogeneity and statistical inconsistency were generally low to moderate, and the network satisfied the assumptions of transitivity and global consistency. However, most comparisons were informed by a small number of trials, many of which had some concerns or a high risk of bias in at least one RoB 2 domain. The complementary frequentist NMAs showed that 95% PIs were typically wide and often crossed the null, even when average effects appeared beneficial. Consistent condom use was the only outcome that met CINeMA requirements, and all network comparisons for this outcome were rated as having low or very low certainty. Together, these features suggest that our estimates reflect uncertain average effects rather than precise predictions for specific programs or settings. These patterns of risk of bias, particularly deviations from intended interventions and missing outcome data, may have led to overestimation of some intervention effects or increased uncertainty in the network estimates and contributed to downgrading the certainty of evidence in our GRADE/CINeMA assessment.

In assessing condom use at the last sexual encounter, TCI emerged as the only approach showing statistically significant improvement compared with NDI. This finding aligns with prior studies reporting absolute increases in condom use among participants receiving SMS or phone-based reminders [80]. The relatively stronger performance of TCI may be attributable to its simplicity and immediacy, directly prompting protective behaviors without requiring advanced digital literacy or prolonged engagement [81]. Previous evidence has also highlighted TCI as one of the more acceptable and widely used forms of digital intervention among young people [30]. Thus, TCIs may offer an immediate behavioral benefit, especially for outcomes tied to the most recent sexual event. Interestingly, while MAI ranked highest in SUCRA probability, its effect did not reach significance, suggesting inconsistency between ranking and statistical evidence. This discrepancy could stem from limited trial numbers, variability in app engagement, or short intervention duration, which may have constrained the power to detect statistical changes. However, this apparent benefit of TCI is based on a few trials with some concerns or high risk of bias, and the wide PIs suggest that similar effects may not be consistently achievable in all implementation settings.

In this study, consistent condom use is improved more effectively by SWI and IOI than by TCI, with SWI ranking the highest. This finding aligns with prior evidence indicating that web-based and online interventions contributed to a substantial proportion of effective digital interventions [21,30]. First, that SWI outperformed IOI may seem counterintuitive, given the absence of interactive features. However, SWI could deliver standardized, theory-based content in a less distracting, user-driven format, allowing youth to process key prevention messages at their own pace. Moreover, while the IOIs included in this review are mostly delivered via computers or tablets, SWIs—though static—are often accessible on smartphones or distributed through popular platforms such as Facebook or WeChat with text, images, or videos [82]. This accessibility and portability may explain why SWI demonstrates stronger effects on consistent condom use. Second, TCI was less effective than both SWI and IOI for improving consistent condom use. Previous studies have suggested that TCI, especially SMS-based reminders, remain controversial in terms of their long-term effectiveness for HIV prevention behaviors [83]. Therefore, although TCI can effectively prompt immediate behaviors, its brief and repetitive messages may lack the depth and reinforcement required to sustain consistent condom use over time. Nevertheless, most trials contributing to this outcome had some concerns or high risk of bias, and CINeMA rated the certainty of these network estimates as low to very low, so the apparent superiority of SWI and IOI should be considered tentative.

When examining the proportion of condom use, IOI demonstrated a significant advantage over SWI, indicating the added value of interactive engagement. This aligns with prior evidence showing that increases in condom use were significantly associated with the use of tailored strategies [21], feedback provision, and guided navigation in digital interventions. Unlike static websites, IOIs integrated tailored feedback, quizzes, or real-time support, which represent core behavior change techniques such as personalized feedback, problem-solving, and self-regulation strategies [84]. These core behavioral change techniques are particularly effective in influencing situational decisions and negotiations during sexual encounters, thereby enhancing the overall proportion of condom use [85,86]. In other words, consistent condom use reflects the internalization of long-term protective norms, and it could be reinforced by standardized and less distracting formats like SWI, while the proportion of condom use is more sensitive to moment-to-moment decision-making. This divergence in findings—SWI being more effective for consistent use, while IOI excels in overall proportion—highlights that different behavioral outcomes may respond to distinct mechanisms of action. Besides, the evolution of technology-based intervention modes has gradually expanded from web-based formats to SMS and social media [87-89]. This trend further supports the notion that while static formats may effectively reinforce long-term norms, interactive platforms provide additional advantages for immediate behavioral decisions during sexual encounters. Yet the CrIs and PIs for this outcome were wide and frequently included the null, indicating considerable heterogeneity and imprecision and implying that any average benefit in the proportion of condom-protected acts may not translate into clear improvements in every context.

Unlike the behavioral outcomes, the effectiveness of DHIs on reducing STI infection reveals a different pattern: NDI and TCI performed more favorably, while IOI and SWI ranked lowest. This finding contrasts with prior studies showing that digital interventions can improve HIV/STI care engagement, such as testing uptake or service use [90-92], highlighting that improvements in care engagement do not necessarily translate into reductions in biological outcomes like STI incidence. Several factors may explain this inconsistency. First, STI incidence represents a distal biological endpoint that may require longer follow-up to capture meaningful reductions, and improvements in self-reported behaviors, such as condom use, may not directly translate into biological protection due to reporting bias or inconsistent application in high-risk contexts. Second, reductions in STI incidence depend not only on safer behaviors but also on timely testing, treatment, and linkage to care. However, evidence shows that stigma related to gender identity, socioeconomic status, race, and ethnicity can delay care-seeking and discourage individuals from accessing necessary services [93]. Nondigital approaches, such as community outreach or peer education programs, often combine behavioral education with direct access to services such as STI testing, treatment linkage, and ongoing support from trained staff or peers, which can directly impact biological outcomes. In contrast, many DHIs focus primarily on education and motivation, without providing structured access to testing or clinical care. Additionally, NDIs may facilitate stronger trust and engagement through in-person interactions, which can overcome barriers related to stigma, confidentiality concerns, or digital literacy limitations. Therefore, while DHIs can effectively change self-reported behaviors, the integrated, multi-component structure of NDIs may explain their relative advantage in reducing actual STI incidence (including HIV). Besides, in this study, TCI is the only digital intervention showing a relatively favorable effect on reducing STI incidence, second only to NDI. Prior studies have demonstrated that TCI can facilitate access to HIV prevention services for youth and achieve high patient and provider satisfaction [93,94]. By providing a comfortable, judgment-free platform, telemedicine may be particularly preferred by marginalized populations, especially transgender youth [95,96]. Given the small and heterogeneous evidence base, important risks of bias in several trials, and wide PIs, these findings on STI incidence should be regarded as exploratory and hypothesis-generating rather than definitive.

Our use of PIs further illustrates the extent to which the observed benefits of DHIs may vary across settings. For most comparisons, the 95% PIs were wide and often crossed the null value, even where the corresponding credible or CIs suggested modest advantages over NDI. This pattern indicates that, although certain DHI modalities tend to improve condom use on average, implementation in new populations or health systems may yield smaller effects or no clear benefit, underscoring the need for careful adaptation, monitoring, and evaluation when scaling up digital prevention programs.

### Implications

These findings have several implications. First, the differential effectiveness of DHIs suggests tailoring intervention types to targeted outcomes. TCI showed immediate benefits for condom use at last sexual contact and a relative advantage for STI incidence, indicating that simple, low-burden interventions may prompt rapid behavior change and reach marginalized youth. In contrast, SWI and IOI were more effective for consistent and habitual condom use, highlighting the value of self-paced content and behavior change techniques that enhance motivation and self-regulation. Multi-modal approaches combining these strengths may maximize overall effectiveness. Second, DHIs alone may be insufficient to reduce STI incidence. Biological outcomes require longer follow-up, and self-reported behavior change does not always translate to infection reduction. Integrating DHIs with offline services, such as condom distribution, PrEP promotion, routine testing, and clinical linkage, is likely necessary to achieve meaningful improvements. Third, these results have implications for intervention design and digital health policy. When targeting youth populations, accessibility, acceptability, and engagement should be prioritized. For example, interventions delivered via mobile platforms or telecommunication may overcome barriers related to stigma or limited digital literacy, while interactive online content can leverage BCTs to support skill acquisition and habitual behavior change. Intervention planners should also consider the balance between immediacy and sustainability of effects: brief, repeated prompts may drive immediate behavior, whereas structured, self-paced content may reinforce long-term habits.

### Limitations

Several limitations of this study should be noted. First, substantial clinical and methodological heterogeneity across trials—including differences in intervention intensity and duration, digital platforms, follow-up periods, and outcome definitions—may have contributed to between-study heterogeneity and could challenge the transitivity assumption underpinning some indirect comparisons in the NMA. Second, we restricted inclusion to randomized, prevention-focused DHIs among HIV-negative or status-unknown youth and excluded nonrandomized studies and trials targeting HIV-positive adolescents; the findings may therefore not generalize to these subgroups or to broader digital programs. Third, all behavioral outcomes relied on self-report, and secondary outcomes such as self-efficacy and number of sexual partners were too sparsely and inconsistently reported to be synthesized, limiting our ability to evaluate the broader psychosocial impact of DHIs beyond condom use. Fourth, the number of trials assessing certain interventions, particularly MAI, was limited, which may reduce statistical power and the precision of effect estimates. Fifth, we were unable to formally assess small-study or publication bias, and selective nonpublication or outcome reporting cannot be ruled out. Finally, most network comparisons were rated as low or very low certainty because of within-study bias and imprecision, and PIs, estimated using standard random-effects methods in netmeta, were wide; the true comparative effects may therefore differ meaningfully from our estimates, underscoring the need for rigorous, adequately powered RCTs with standardized outcomes and longer follow-up.

### Conclusions

In conclusion, this NMA provides the most comprehensive synthesis to date on the comparative effectiveness of DHIs in promoting safer sexual behaviors among youth. This NMA highlights that the effectiveness of DHIs for HIV prevention among youth depends on both intervention modality and targeted outcomes. While DHIs can enhance knowledge and protective behaviors, their impact on biological endpoints remains limited without integration with offline services and broader structural support. Tailoring interventions to behavioral targets, engagement strategies, and contextual factors is essential to maximize their potential in promoting youth sexual health. We hope that these results will inform the design of youth-centered digital prevention programs, guide clinicians and educators in selecting appropriate modalities, and support policymakers and guideline developers in integrating digital strategies into national HIV prevention frameworks. Future studies should focus on the specific characteristics of patients to provide personalized estimates of comparative effectiveness and individualized predictions regarding the probability of response to treatment and of side effects.

## Supplementary material

10.2196/87071 Multimedia Appendix 1 Search String.

10.2196/87071 Multimedia Appendix 2 Reference list of excluded studies.

10.2196/87071 Multimedia Appendix 3 Risk of bias, CINeMA assessments, and GRADE Summary of Findings tables.

10.2196/87071 Multimedia Appendix 4 Reference list of included studies.

10.2196/87071 Multimedia Appendix 5 Indirect comparative effectiveness DHIs.

10.2196/87071 Multimedia Appendix 6 Forest plots of posterior ORs with 95.

10.2196/87071 Multimedia Appendix 7 Random‐effects network meta‐analysis of digital health interventions versus non‐digital interventions.

10.2196/87071 Multimedia Appendix 8 Model fit diagnostics for consistency and inconsistency models in the network meta.

10.2196/87071 Checklist 1 PRISMA-NMA checklist.

## Abbreviations

CINeMA: Confidence in Network Meta-Analysis
CrI: credible interval
DHI: digital health intervention
DIC: deviance information criterion
IOI: interactive online-based intervention
MAI: mobile app-based intervention
NDI: nondigital intervention
NMA: network meta-analysis
OR: odds ratio
PI: prediction interval
PRISMA: Preferred Reporting Items for Systematic Reviews and Meta-Analyzes
PRISMA-NMA: PRISMA extension for network meta-analyzes
RCT: randomized controlled trial
RoB 2: Cochrane Risk of Bias 2 tool
SR: systematic review
STI: sexually transmitted infection
SUCRA: surface under the cumulative ranking curve
SWI: static web-based intervention
TCI: telecommunication-based intervention
