# Supplementary material for: Effects of Digital Health Interventions to Promote Safer Sex Behaviors Among Youth: Systematic Review and Bayesian Network Meta-Analysis
Source: J Med Internet Res. 2026 Feb 4;28:e87071. doi: 10.2196/87071 (PMC12871581; doi:10.2196/87071)
Supplement: Multimedia Appendix 1 [file jmir-v28-e87071-s001.docx]

**Search String**

(a) PubMed

| **Concept** | **Search number** | **Search terms** |
| --- | --- | --- |
| Population | #1 | ("Adolescent"[MeSH Terms] OR "Young Adult"[MeSH Terms]) OR ("adolesc*"[Title/Abstract] OR adolescence[Title/Abstract] OR adolescent[Title/Abstract] OR adolescents[Title/Abstract] OR "AYA"[Title/Abstract] OR "city college"[Title/Abstract] OR college[Title/Abstract] OR "college student"[Title/Abstract] OR "young adult"[Title/Abstract] OR "young adulthood"[Title/Abstract] OR "young people"[Title/Abstract] OR "young person"[Title/Abstract] OR “young women”[Title/Abstract] OR “young men”[Title/Abstract] OR "school*"[Title/Abstract] OR "boy"[Title/Abstract] OR "boys"[Title/Abstract] OR "girl*"[Title/Abstract] OR "youth*"[Title/Abstract] OR "young*"[Title/Abstract] OR "student*"[Title/Abstract] OR "college students"[Title/Abstract] OR "community college*"[Title/Abstract] OR "university"[Title/Abstract] OR "eighth grade*"[Title/Abstract] OR "eleventh grade*"[Title/Abstract] OR freshman[Title/Abstract] OR freshmen[Title/Abstract] OR "grade 10"[Title/Abstract] OR "grade 11"[Title/Abstract] OR "grade 12"[Title/Abstract] OR "grade 7"[Title/Abstract] OR "grade 8"[Title/Abstract] OR "grade 9"[Title/Abstract] OR "grade eight"[Title/Abstract] OR "grade eleven"[Title/Abstract] OR "grade nine"[Title/Abstract] OR "grade seven"[Title/Abstract] OR "grade ten"[Title/Abstract] OR "grade twelve"[Title/Abstract] OR "high school"[Title/Abstract] OR "high-school"[Title/Abstract] OR "high school students"[Title/Abstract] OR "junior college"[Title/Abstract] OR "junior high"[Title/Abstract] OR “junior*”[Title/Abstract] OR "middle school"[Title/Abstract] OR "ninth grade*"[Title/Abstract] OR "senior high school”[Title/Abstract] OR “senior*”[Title/Abstract] OR ”seventh grade*"[Title/Abstract] OR sophomores[Title/Abstract] OR "technical college"[Title/Abstract] OR “teen*”[Title/Abstract] OR "tenth grade*"[Title/Abstract] OR "two-year college"[Title/Abstract] OR "twelfth grade*"[Title/Abstract]) |
| Intervention | #2 | "mhealth"[Title/Abstract] OR "m-health"[Title/Abstract] OR "ehealth"[Title/Abstract] OR "e-health"[Title/Abstract] OR "electronic health*"[Title/Abstract] OR "e-Portal"[Title/Abstract] OR "ePortal"[Title/Abstract] OR "e-consultation"[Title/Abstract] OR "Remote Consultation"[Title/Abstract] OR "e-care"[Title/Abstract] OR "etherap*"[Title/Abstract] OR "e therap*"[Title/Abstract] OR "eHealth literacy"[Title/Abstract] OR "e-literacy"[Title/Abstract] |
|  | #3 | "Internet"[MeSH Terms] OR "Internet-Based Intervention"[MeSH Terms] OR "web-based"[Title/Abstract] OR "internet-based"[Title/Abstract] OR "internet"[Title/Abstract] OR "web"[Title/Abstract] OR "webs"[Title/Abstract] OR "website*"[Title/Abstract] OR "online"[Title/Abstract] OR "on-line communication"[Title/Abstract] OR "online communication"[Title/Abstract] OR "social network*"[Title/Abstract] |
|  | #4 | "Video Games"[MeSH Terms] OR "games, experimental"[MeSH Terms] OR "games, recreational"[MeSH Terms] OR "exergaming"[MeSH Terms] OR "gamification"[MeSH Terms] OR "game*"[Title/Abstract] OR "gaming"[Title/Abstract] OR "interactive video*"[Title/Abstract] OR "virtual reality"[Title/Abstract] OR "board games"[Title/Abstract] OR "tabletop games"[Title/Abstract] OR "go game"[Title/Abstract] OR "videogame*"[Title/Abstract] OR "video game*"[Title/Abstract] OR "electronic game*"[Title/Abstract] OR "computer game*"[Title/Abstract] OR "online game*"[Title/Abstract] OR "internet game*"[Title/Abstract] OR "online gaming"[Title/Abstract] OR "exergame*"[Title/Abstract] OR "exergaming"[Title/Abstract] OR "serious game*"[Title/Abstract] OR "serious gaming"[Title/Abstract] OR "gamification"[Title/Abstract] |
|  | #5 | "mass media"[MeSH Terms] OR "multimedia"[MeSH Terms] OR "forum*"[Title/Abstract] OR "multi-media"[Title/Abstract] OR "multimedia"[Title/Abstract] OR "mass media"[Title/Abstract] |
|  | #6 | "Text Messaging"[MeSH Terms] OR "text messag*"[Title/Abstract] OR "short message service"[Title/Abstract] OR "SMS"[Title/Abstract] OR "instant messag*"[Title/Abstract] OR "mms"[Title/Abstract] OR "text"[Title/Abstract] OR "messag*"[Title/Abstract] |
|  | #7 | "Electronic Mail"[MeSH Terms] OR "email*"[Title/Abstract] OR "e mail*"[Title/Abstract] OR "electronic mail*"[Title/Abstract] |
|  | #8 | "Computers"[MeSH Terms] OR "computers, handheld"[MeSH Terms] OR "Computer-Assisted Instruction"[MeSH Terms] OR "computer system*"[MeSH Terms] OR "microcomputers"[MeSH Terms] OR "cd rom"[MeSH Terms] OR "computer*"[Title/Abstract] OR "tablet*"[Title/Abstract] OR "handheld"[Title/Abstract] OR "technology"[Title/Abstract] OR "electronic tablet*"[Title/Abstract] |
|  | #9 | "telemedicine"[MeSH Terms] OR "Telerehabilitation"[MeSH Terms] OR "Cell Phone"[MeSH Terms] OR "Telephone"[MeSH Terms] OR "Smartphone"[MeSH Terms] OR "mobile phone"[Title/Abstract] OR "mobile telephone*"[Title/Abstract] OR "smartphone*"[Title/Abstract] OR "Telephone"[Title/Abstract] OR "phone"[Title/Abstract] OR "cell*"[Title/Abstract] OR "mobile device*"[Title/Abstract] OR"telemedicine"[Title/Abstract] OR "tele-medicine"[Title/Abstract] OR "telecare"[Title/Abstract] OR "tele-care"[Title/Abstract] OR "telehomecare"[Title/Abstract] OR "tele-homecare"[Title/Abstract] OR "telemonitoring"[Title/Abstract] OR "tele-monitoring"[Title/Abstract] OR "telehealth*"[Title/Abstract] OR "tele-health*"[Title/Abstract] OR "Telecommunications"[Title/Abstract] OR "teletherap*"[Title/Abstract] OR "tele-rehabilitation"[Title/Abstract] OR "Telerehabilitation"[Title/Abstract] OR "technological skill*"[Title/Abstract] OR "technology competenc*"[Title/Abstract] OR "technology skill*"[Title/Abstract] OR "telehealth competenc*"[Title/Abstract] OR "telemedicine skill*"[Title/Abstract] OR "telemedicine competenc*"[Title/Abstract] OR "android*"[Title/Abstract] OR "ipad*"[Title/Abstract] OR "iphone*"[Title/Abstract] OR "ipod*"[Title/Abstract] OR "ios"[Title/Abstract] |
|  | #10 | "mobile applications"[MeSH Terms] OR "communications media"[MeSH Terms] OR "mobile application*"[Title/Abstract] OR "mobile"[Title/Abstract] OR "wireless technology"[MeSH Terms] OR "internet"[MeSH Terms] OR "social media"[MeSH Terms] OR "mobile health application"[Title/Abstract] OR "mobile applications"[MeSH Terms] OR "app"[Title/Abstract] OR "apps"[Title/Abstract] OR "social media"[Title/Abstract] OR "media competenc*"[Title/Abstract] OR "media skill*"[Title/Abstract] OR "media literacy*"[Title/Abstract] OR "whatsapp"[Title/Abstract] OR "facebook"[Title/Abstract] OR "grindr"[Title/Abstract] OR "telegram"[Title/Abstract] OR "tiktok"[Title/Abstract] OR "geosocial"[Title/Abstract] OR "mxit"[Title/Abstract] OR "twitter"[Title/Abstract] OR "tweet"[Title/Abstract] OR "program*"[Title/Abstract] OR "Jack'd"[Title/Abstract] OR "WeChat"[Title/Abstract] OR "Blued"[Title/Abstract] OR "chat room*"[Title/Abstract] OR "chatroom*"[Title/Abstract] OR "pda"[Title/Abstract] |
|  | #11 | "digital health"[MeSH Terms] OR "digital technology"[MeSH Terms] OR "artificial intelligence"[MeSH Terms] OR "robotics"[MeSH Terms] OR "machine learning"[MeSH Terms] OR "virtual reality"[MeSH Terms] OR "digital health"[Title/Abstract] OR "digital technology"[Title/Abstract] OR "digital intervention"[Title/Abstract] OR "digital media"[Title/Abstract] OR "digital assistant*"[Title/Abstract] OR "Digital Health Literacy"[Title/Abstract] OR "digital literac*"[Title/Abstract] OR "digital competenc*"[Title/Abstract] OR "digital skill*"[Title/Abstract] OR "artificial intelligence"[Title/Abstract] OR "robotics"[Title/Abstract] OR "machine learning"[Title/Abstract] OR "virtual reality"[Title/Abstract] |
|  | #12 | #2 OR #3 OR #4 OR #5 OR #6 OR #7 OR #8 OR #9 OR #10 OR #11 |
| Outcome | #13 | "condoms"[MeSH Terms] OR "condoms"[MeSH Major Topic] OR "safe sex"[MeSH Terms] OR "Unsafe Sex"[MeSH Terms] OR "Coitus"[MeSH Terms] OR "Sexual Partners"[MeSH Terms] OR "Sexual Behavior"[MeSH Terms] OR "Risk-Taking"[MeSH Terms] OR "condom*"[Text word] OR "condom use self-efficacy" [Title/Abstract] OR "condomless sex"[Title/Abstract] OR "condom use"[Title/Abstract] OR "condom compliance" [Title/Abstract] OR "condom behavior" [Title/Abstract] OR ("sex"[Title/Abstract] AND ("risk*"[Title/Abstract] OR "work*"[Title/Abstract] OR "behavior*"[Title/Abstract])) OR "risk behavior*"[Title/Abstract] OR "risky behavior*"[Title/Abstract] OR "Risk-Taking"[Title/Abstract] OR "safe* sex*"[Title/Abstract] OR "unsafe* sex*"[Title/Abstract] OR "protected sex*"[Title/Abstract] OR "protected intercourse"[Title/Abstract] OR "unprotected sex*"[Title/Abstract] OR "unprotected intercourse"[Title/Abstract] OR "Coitus"[Title/Abstract] OR "sexual partner*"[Title/Abstract] OR "sex partner*"[Title/Abstract] OR "multiple sexual partner*"[Title/Abstract] OR "multiple sex partner*"[Title/Abstract] OR "multiple partner*"[Title/Abstract] OR "sexually transmitted"[Title/Abstract] OR "STIs"[Title/Abstract] OR "STI"[Title/Abstract] OR "STDs"[Title/Abstract] OR "STD"[Title/Abstract] OR "chlamydia"[Title/Abstract] OR "gonorrhea"[Title/Abstract] OR "syphilis"[Title/Abstract] OR "human immunodeficiency virus*"[Title/Abstract] OR "human immune deficiency virus*"[Title/Abstract] OR "HIV"[Title/Abstract] OR "AIDS"[Title/Abstract] OR "acquired immunodeficiency syndrome"[Title/Abstract] OR "acquired immune deficiency syndrome"[Title/Abstract] |
| Study design | #14 | "Clinical Trials as Topic"[MeSH Terms] OR "randomized controlled trial"[Publication Type] OR "controlled clinical trial"[Publication Type] OR "randomized"[Title/Abstract] OR "placebo"[Title/Abstract] OR "randomly"[Title/Abstract] OR "trial"[Title] |
|  | #15 | "Animals"[MeSH Terms] NOT "Humans"[MeSH Terms] |
|  | #16 | #14 NOT #15 |
| Combined | #17 | #1 AND #12 AND #13 AND #16 |

(b) EMBASE

| **Concept** | **Search number** | **Search terms** |
| --- | --- | --- |
| Population | #1 | 'adolescent'/exp OR 'adolescent' OR 'young adult'/exp OR 'young adult' OR 'adolesc*':ti,ab,kw OR adolescence:ti,ab,kw OR adolescent:ti,ab,kw OR adolescents:ti,ab,kw OR 'aya':ti,ab,kw OR 'city college':ti,ab,kw OR college:ti,ab,kw OR 'college student':ti,ab,kw OR 'young adult':ti,ab,kw OR 'young adulthood':ti,ab,kw OR 'young people':ti,ab,kw OR 'young person':ti,ab,kw OR 'young women':ti,ab,kw OR 'young men':ti,ab,kw OR 'school*':ti,ab,kw OR 'boy':ti,ab,kw OR 'boys':ti,ab,kw OR 'girl*':ti,ab,kw OR 'youth*':ti,ab,kw OR 'young*':ti,ab,kw OR 'student*':ti,ab,kw OR 'college students':ti,ab,kw OR 'community college*':ti,ab,kw OR 'university':ti,ab,kw OR 'eighth grade*':ti,ab,kw OR 'eleventh grade*':ti,ab,kw OR freshman:ti,ab,kw OR freshmen:ti,ab,kw OR 'grade 10':ti,ab,kw OR 'grade 11':ti,ab,kw OR 'grade 12':ti,ab,kw OR 'grade 7':ti,ab,kw OR 'grade 8':ti,ab,kw OR 'grade 9':ti,ab,kw OR 'grade eight':ti,ab,kw OR 'grade eleven':ti,ab,kw OR 'grade nine':ti,ab,kw OR 'grade seven':ti,ab,kw OR 'grade ten':ti,ab,kw OR 'grade twelve':ti,ab,kw OR 'high school':ti,ab,kw OR 'high-school':ti,ab,kw OR 'high school students':ti,ab,kw OR 'junior college':ti,ab,kw OR 'junior high':ti,ab,kw OR 'junior*':ti,ab,kw OR 'middle school':ti,ab,kw OR 'ninth grade*':ti,ab,kw OR 'senior high school':ti,ab,kw OR 'senior*':ti,ab,kw OR 'seventh grade*':ti,ab,kw OR sophomores:ti,ab,kw OR 'technical college':ti,ab,kw OR 'teen*':ti,ab,kw OR 'tenth grade*':ti,ab,kw OR 'two-year college':ti,ab,kw OR 'twelfth grade*':ti,ab,kw |
| Intervention | #2 | 'mhealth':ti,ab,kw OR 'm-health':ti,ab,kw OR 'ehealth':ti,ab,kw OR 'e-health':ti,ab,kw OR 'electronic health*':ti,ab,kw OR 'e-portal':ti,ab,kw OR 'eportal':ti,ab,kw OR 'e-consultation':ti,ab,kw OR 'remote consultation':ti,ab,kw OR 'e-care':ti,ab,kw OR 'etherap*':ti,ab,kw OR 'e therap*':ti,ab,kw OR 'ehealth literacy':ti,ab,kw OR 'e-literacy':ti,ab,kw |
|  | #3 | 'internet'/exp OR 'internet-based intervention'/exp OR 'web-based':ti,ab,kw OR 'internet-based':ti,ab,kw OR 'internet':ti,ab,kw OR 'web':ti,ab,kw OR 'webs':ti,ab,kw OR 'website*':ti,ab,kw OR 'online':ti,ab,kw OR 'on-line communication':ti,ab,kw OR 'online communication':ti,ab,kw OR 'social network*':ti,ab,kw |
|  | #4 | 'video games'/exp OR 'games, experimental'/exp OR 'games, recreational'/exp OR 'exergaming'/exp OR 'gamification'/exp OR 'game*':ti,ab,kw OR 'gaming':ti,ab,kw OR 'interactive video*':ti,ab,kw OR 'virtual reality':ti,ab,kw OR 'board games':ti,ab,kw OR 'tabletop games':ti,ab,kw OR 'go game':ti,ab,kw OR 'videogame*':ti,ab,kw OR 'video game*':ti,ab,kw OR 'electronic game*':ti,ab,kw OR 'computer game*':ti,ab,kw OR 'online game*':ti,ab,kw OR 'internet game*':ti,ab,kw OR 'online gaming':ti,ab,kw OR 'exergame*':ti,ab,kw OR 'exergaming':ti,ab,kw OR 'serious game*':ti,ab,kw OR 'serious gaming':ti,ab,kw OR 'gamification':ti,ab,kw |
|  | #5 | 'mass media'/exp OR 'multimedia'/exp OR 'forum*':ti,ab,kw OR 'multi-media':ti,ab,kw OR 'multimedia':ti,ab,kw OR 'mass media':ti,ab,kw |
|  | #6 | 'text messaging'/exp OR 'text messag*':ti,ab,kw OR 'short message service':ti,ab,kw OR 'sms':ti,ab,kw OR 'instant messag*':ti,ab,kw OR 'mms':ti,ab,kw OR 'text':ti,ab,kw OR 'messag*':ti,ab,kw |
|  | #7 | 'electronic mail'/exp OR 'email*':ti,ab,kw OR 'e mail*':ti,ab,kw OR 'electronic mail*':ti,ab,kw |
|  | #8 | 'computers'/exp OR 'computers, handheld'/exp OR 'computer-assisted instruction'/exp OR 'computer system*'/exp OR 'microcomputers'/exp OR 'cd rom'/exp OR 'computer*':ti,ab,kw OR 'tablet*':ti,ab,kw OR 'handheld':ti,ab,kw OR 'technology':ti,ab,kw OR 'electronic tablet*':ti,ab,kw |
|  | #9 | 'telemedicine'/exp OR 'telerehabilitation'/exp OR 'cell phone'/exp OR 'telephone'/exp OR 'smartphone'/exp OR 'mobile phone':ti,ab,kw OR 'mobile telephone*':ti,ab,kw OR 'smartphone*':ti,ab,kw OR 'telephone':ti,ab,kw OR 'phone':ti,ab,kw OR 'cell*':ti,ab,kw OR 'mobile device*':ti,ab,kw OR 'telemedicine':ti,ab,kw OR 'tele-medicine':ti,ab,kw OR 'telecare':ti,ab,kw OR 'tele-care':ti,ab,kw OR 'telehomecare':ti,ab,kw OR 'tele-homecare':ti,ab,kw OR 'telemonitoring':ti,ab,kw OR 'tele-monitoring':ti,ab,kw OR 'telehealth*':ti,ab,kw OR 'tele-health*':ti,ab,kw OR 'telecommunications':ti,ab,kw OR 'teletherap*':ti,ab,kw OR 'tele-rehabilitation':ti,ab,kw OR 'telerehabilitation':ti,ab,kw OR 'technological skill*':ti,ab,kw OR 'technology competenc*':ti,ab,kw OR 'technology skill*':ti,ab,kw OR 'telehealth competenc*':ti,ab,kw OR 'telemedicine skill*':ti,ab,kw OR 'telemedicine competenc*':ti,ab,kw OR 'android*':ti,ab,kw OR 'ipad*':ti,ab,kw OR 'iphone*':ti,ab,kw OR 'ipod*':ti,ab,kw OR 'ios':ti,ab,kw |
|  | #10 | 'communications media'/exp OR 'mobile application*':ti,ab,kw OR 'mobile':ti,ab,kw OR 'wireless technology'/exp OR 'internet'/exp OR 'social media'/exp OR 'mobile health application':ti,ab,kw OR 'mobile applications'/exp OR 'app':ti,ab,kw OR 'apps':ti,ab,kw OR 'social media':ti,ab,kw OR 'media competenc*':ti,ab,kw OR 'media skill*':ti,ab,kw OR 'media literacy*':ti,ab,kw OR 'whatsapp':ti,ab,kw OR 'facebook':ti,ab,kw OR 'grindr':ti,ab,kw OR 'telegram':ti,ab,kw OR 'tiktok':ti,ab,kw OR 'geosocial':ti,ab,kw OR 'mxit':ti,ab,kw OR 'twitter':ti,ab,kw OR 'tweet':ti,ab,kw OR 'program*':ti,ab,kw OR 'jack d':ti,ab,kw OR 'wechat':ti,ab,kw OR 'blued':ti,ab,kw OR 'chat room*':ti,ab,kw OR 'chatroom*':ti,ab,kw OR 'pda':ti,ab,kw |
|  | #11 | 'digital health'/exp OR 'digital technology'/exp OR 'artificial intelligence'/exp OR 'robotics'/exp OR 'machine learning'/exp OR 'virtual reality'/exp OR 'digital health':ti,ab,kw OR 'digital technology':ti,ab,kw OR 'digital intervention':ti,ab,kw OR 'digital media':ti,ab,kw OR 'digital assistant*':ti,ab,kw OR 'digital health literacy':ti,ab,kw OR 'digital literac*':ti,ab,kw OR 'digital competenc*':ti,ab,kw OR 'digital skill*':ti,ab,kw OR 'artificial intelligence':ti,ab,kw OR 'robotics':ti,ab,kw OR 'machine learning':ti,ab,kw OR 'virtual reality':ti,ab,kw |
|  | #12 | #2 OR #3 OR #4 OR #5 OR #6 OR #7 OR #8 OR #9 OR #10 OR #11 |
| Outcome | #13 | 'condoms'/exp OR 'safe sex'/exp OR 'unsafe sex'/exp OR 'coitus'/exp OR 'sexual partners'/exp OR 'sexual behavior'/exp OR 'risk-taking'/exp OR 'condom*':ti,ab,kw OR 'condom use self-efficacy':ti,ab,kw OR 'condomless sex':ti,ab,kw OR 'condom use':ti,ab,kw OR 'condom compliance':ti,ab,kw OR 'condom behavior':ti,ab,kw OR ('sex':ti,ab,kw AND ('risk*':ti,ab,kw OR 'work*':ti,ab,kw OR 'behavior*':ti,ab,kw)) OR 'risk behavior*':ti,ab,kw OR 'risky behavior*':ti,ab,kw OR 'risk-taking':ti,ab,kw OR 'safe* sex*':ti,ab,kw OR 'unsafe* sex*':ti,ab,kw OR 'protected sex*':ti,ab,kw OR 'protected intercourse':ti,ab,kw OR 'unprotected sex*':ti,ab,kw OR 'unprotected intercourse':ti,ab,kw OR 'coitus':ti,ab,kw OR 'sexual partner*':ti,ab,kw OR 'sex partner*':ti,ab,kw OR 'multiple sexual partner*':ti,ab,kw OR 'multiple sex partner*':ti,ab,kw OR 'multiple partner*':ti,ab,kw OR 'sexually transmitted':ti,ab,kw OR 'stis':ti,ab,kw OR 'sti':ti,ab,kw OR 'stds':ti,ab,kw OR 'std':ti,ab,kw OR 'chlamydia':ti,ab,kw OR 'gonorrhea':ti,ab,kw OR 'syphilis':ti,ab,kw OR 'human immunodeficiency virus*':ti,ab,kw OR 'human immune deficiency virus*':ti,ab,kw OR 'hiv':ti,ab,kw OR 'aids':ti,ab,kw OR 'acquired immunodeficiency syndrome':ti,ab,kw OR 'acquired immune deficiency syndrome':ti,ab,kw |
| Study design | #14 | 'randomized controlled trial'/exp OR 'controlled trial, randomized' OR 'randomised controlled study' OR 'randomised controlled trial' OR 'randomized controlled study' OR 'randomized controlled trial' OR 'trial, randomized controlled' OR 'controlled clinical trial (topic)'/exp OR 'controlled clinical trial (topic)' OR 'controlled clinical trials' OR 'controlled clinical trials as topic' OR 'non randomized controlled trials as topic' OR 'non-randomized controlled trials as topic' OR 'experimental design'/exp OR 'doe approach' OR 'design of experiment' OR 'experimental design' |
| Combined | #15 | #1 AND #12 AND #13 AND #14 |

(c) Web of Science

| **Concept** | **Search number** | **Search terms** |
| --- | --- | --- |
| Population | #1 | TS=("Adolescent" OR "Young Adult") OR TI=("adolesc*" OR adolescence OR adolescent OR adolescents OR "AYA" OR "city college" OR college OR "college student" OR "young adult" OR "young adulthood" OR "young people" OR "young person" OR “young women” OR “young men” OR "school*" OR "boy" OR "boys" OR "girl*" OR "youth*" OR "young*" OR "student*" OR "college students" OR "community college*" OR "university" OR "eighth grade*" OR "eleventh grade*" OR freshman OR freshmen OR "grade 10" OR "grade 11" OR "grade 12" OR "grade 7" OR "grade 8" OR "grade 9" OR "grade eight" OR "grade eleven" OR "grade nine" OR "grade seven" OR "grade ten" OR "grade twelve" OR "high school" OR "high-school" OR "high school students" OR "junior college" OR "junior high" OR “junior*” OR "middle school" OR "ninth grade*" OR "senior high school” OR “senior*” OR ”seventh grade*" OR sophomores OR "technical college" OR “teen*” OR "tenth grade*" OR "two-year college" OR "twelfth grade*") OR AB=("adolesc*" OR adolescence OR adolescent OR adolescents OR "AYA" OR "city college" OR college OR "college student" OR "young adult" OR "young adulthood" OR "young people" OR "young person" OR “young women” OR “young men” OR "school*" OR "boy" OR "boys" OR "girl*" OR "youth*" OR "young*" OR "student*" OR "college students" OR "community college*" OR "university" OR "eighth grade*" OR "eleventh grade*" OR freshman OR freshmen OR "grade 10" OR "grade 11" OR "grade 12" OR "grade 7" OR "grade 8" OR "grade 9" OR "grade eight" OR "grade eleven" OR "grade nine" OR "grade seven" OR "grade ten" OR "grade twelve" OR "high school" OR "high-school" OR "high school students" OR "junior college" OR "junior high" OR “junior*” OR "middle school" OR "ninth grade*" OR "senior high school” OR “senior*” OR ”seventh grade*" OR sophomores OR "technical college" OR “teen*” OR "tenth grade*" OR "two-year college" OR "twelfth grade*") |
| Intervention | #2 | TI=("mhealth" OR "m-health" OR "ehealth" OR "e-health" OR "electronic health*" OR "e-Portal" OR "ePortal" OR "e-consultation" OR "Remote Consultation" OR "e-care" OR "etherap*" OR "e therap*" OR "eHealth literacy" OR "e-literacy") OR AB=("mhealth" OR "m-health" OR "ehealth" OR "e-health" OR "electronic health*" OR "e-Portal" OR "ePortal" OR "e-consultation" OR "Remote Consultation" OR "e-care" OR "etherap*" OR "e therap*" OR "eHealth literacy" OR "e-literacy") |
|  | #3 | TS=("Internet" OR "Internet-Based Intervention") OR TI=("Internet-Based Intervention" OR "web-based" OR "internet-based" OR "internet" OR "web" OR "webs" OR "website*" OR "online" OR "on-line communication" OR "online communication" OR "social network*") OR AB=("Internet-Based Intervention" OR "web-based" OR "internet-based" OR "internet" OR "web" OR "webs" OR "website*" OR "online" OR "on-line communication" OR "online communication" OR "social network*") |
|  | #4 | TS=("Video Games" OR "games, experimental" OR "games, recreational" OR "exergaming" OR "gamification") OR TI=("game*" OR "gaming" OR "interactive video*" OR "virtual reality" OR "board games" OR "tabletop games" OR "go game" OR "videogame*" OR "video game*" OR "electronic game*" OR "computer game*" OR "online game*" OR "internet game*" OR "online gaming" OR "exergame*" OR "exergaming" OR "serious game*" OR "serious gaming" OR "gamification") OR AB=("game*" OR "gaming" OR "interactive video*" OR "virtual reality" OR "board games" OR "tabletop games" OR "go game" OR "videogame*" OR "video game*" OR "electronic game*" OR "computer game*" OR "online game*" OR "internet game*" OR "online gaming" OR "exergame*" OR "exergaming" OR "serious game*" OR "serious gaming" OR "gamification") |
|  | #5 | TS=("mass media" OR "multimedia") OR TI=("forum*" OR "multi-media" OR "multimedia" OR "mass media") OR AB=("forum*" OR "multi-media" OR "multimedia" OR "mass media") |
|  | #6 | TS=("Text Messaging") OR TI=("text messag*" OR "short message service" OR "SMS" OR "instant messag*" OR "mms" OR "text" OR "messag*") OR AB=("text messag*" OR "short message service" OR "SMS" OR "instant messag*" OR "mms" OR "text" OR "messag*") |
|  | #7 | TS=("Electronic Mail") OR TI=("email*" OR "e mail*" OR "electronic mail*") OR AB=("email*" OR "e mail*" OR "electronic mail*") |
|  | #8 | TS=("Computers" OR "computers, handheld" OR "Computer-Assisted Instruction" OR "computer system*" OR "microcomputers" OR "cd rom") OR TI=("computer*" OR "tablet*" OR "handheld" OR "technology" OR "electronic tablet*") OR AB=("computer*" OR "tablet*" OR "handheld" OR "technology" OR "electronic tablet*") |
|  | #9 | TS=("telemedicine" OR "Telerehabilitation" OR "Cell Phone" OR "Telephone" OR "Smartphone") OR TI=("mobile phone" OR "mobile telephone*" OR "smartphone*" OR "Telephone" OR "phone" OR "cell*" OR "mobile device*" OR"telemedicine" OR "tele-medicine" OR "telecare" OR "tele-care" OR "telehomecare" OR "tele-homecare" OR "telemonitoring" OR "tele-monitoring" OR "telehealth*" OR "tele-health*" OR "Telecommunications" OR "teletherap*" OR "tele-rehabilitation" OR "Telerehabilitation" OR "technological skill*" OR "technology competenc*" OR "technology skill*" OR "telehealth competenc*" OR "telemedicine skill*" OR "telemedicine competenc*" OR "android*" OR "ipad*" OR "iphone*" OR "ipod*" OR "ios") OR AB=("mobile phone" OR "mobile telephone*" OR "smartphone*" OR "Telephone" OR "phone" OR "cell*" OR "mobile device*" OR"telemedicine" OR "tele-medicine" OR "telecare" OR "tele-care" OR "telehomecare" OR "tele-homecare" OR "telemonitoring" OR "tele-monitoring" OR "telehealth*" OR "tele-health*" OR "Telecommunications" OR "teletherap*" OR "tele-rehabilitation" OR "Telerehabilitation" OR "technological skill*" OR "technology competenc*" OR "technology skill*" OR "telehealth competenc*" OR "telemedicine skill*" OR "telemedicine competenc*" OR "android*" OR "ipad*" OR "iphone*" OR "ipod*" OR "ios") |
|  | #10 | TS=("mobile applications" OR "communications media" OR "mobile application*" OR "mobile" OR "wireless technology" OR "internet" OR "social media" OR "mobile health application" OR "mobile applications") OR TI=("app" OR "apps" OR "social media" OR "media competenc*" OR "media skill*" OR "media literacy*" OR "whatsapp" OR "facebook" OR "grindr" OR "telegram" OR "tiktok" OR "geosocial" OR "mxit" OR "twitter" OR "tweet" OR "program*" OR "Jack'd" OR "WeChat" OR "Blued" OR "chat room*" OR "chatroom*" OR "pda") OR AB=("app" OR "apps" OR "social media" OR "media competenc*" OR "media skill*" OR "media literacy*" OR "whatsapp" OR "facebook" OR "grindr" OR "telegram" OR "tiktok" OR "geosocial" OR "mxit" OR "twitter" OR "tweet" OR "program*" OR "Jack'd" OR "WeChat" OR "Blued" OR "chat room*" OR "chatroom*" OR "pda" |
|  | #11 | TS=("digital health" OR "digital technology" OR "artificial intelligence" OR "robotics" OR "machine learning" OR "virtual reality") OR TI=("digital health" OR "digital technology" OR "digital intervention" OR "digital media" OR "digital assistant*" OR "Digital Health Literacy" OR "digital literac*" OR "digital competenc*" OR "digital skill*" OR "artificial intelligence" OR "robotics" OR "machine learning" OR "virtual reality") OR AB=("digital health" OR "digital technology" OR "digital intervention" OR "digital media" OR "digital assistant*" OR "Digital Health Literacy" OR "digital literac*" OR "digital competenc*" OR "digital skill*" OR "artificial intelligence" OR "robotics" OR "machine learning" OR "virtual reality") |
|  | #12 | #2 OR #3 OR #4 OR #5 OR #6 OR #7 OR #8 OR #9 OR #10 OR #11 |
| Outcome | #13 | TS=("condoms" OR "safe sex" OR "Unsafe Sex" OR "Coitus" OR "Sexual Partners" OR "Sexual Behavior" OR "Risk-Taking" OR "condom*") OR TI=("condom use self-efficacy" OR "condomless sex" OR "condom use" OR "condom compliance" OR "condom behavior" OR ("sex" AND ("risk*" OR "work*" OR "behavior*")) OR "risk behavior*" OR "risky behavior*" OR "Risk-Taking" OR "safe* sex*" OR "unsafe* sex*" OR "protected sex*" OR "protected intercourse" OR "unprotected sex*" OR "unprotected intercourse" OR "Coitus" OR "sexual partner*" OR "sex partner*" OR "multiple sexual partner*" OR "multiple sex partner*" OR "multiple partner*" OR "sexually transmitted" OR "STIs" OR "STI" OR "STDs" OR "STD" OR "chlamydia" OR "gonorrhea" OR "syphilis" OR "human immunodeficiency virus*" OR "human immune deficiency virus*" OR "HIV" OR "AIDS" OR "acquired immunodeficiency syndrome" OR "acquired immune deficiency syndrome") OR AB=("condom use self-efficacy" OR "condomless sex" OR "condom use" OR "condom compliance" OR "condom behavior" OR ("sex" AND ("risk*" OR "work*" OR "behavior*")) OR "risk behavior*" OR "risky behavior*" OR "Risk-Taking" OR "safe* sex*" OR "unsafe* sex*" OR "protected sex*" OR "protected intercourse" OR "unprotected sex*" OR "unprotected intercourse" OR "Coitus" OR "sexual partner*" OR "sex partner*" OR "multiple sexual partner*" OR "multiple sex partner*" OR "multiple partner*" OR "sexually transmitted" OR "STIs" OR "STI" OR "STDs" OR "STD" OR "chlamydia" OR "gonorrhea" OR "syphilis" OR "human immunodeficiency virus*" OR "human immune deficiency virus*" OR "HIV" OR "AIDS" OR "acquired immunodeficiency syndrome" OR "acquired immune deficiency syndrome") |
| Study design | #14 | (TS=("Clinical Trials as Topic") OR SO=("randomized controlled trial" OR "controlled clinical trial") OR AB=("randomized" OR "placebo" OR "randomly") OR TI=("randomized" OR "placebo" OR "randomly" OR "trial")) NOT ((TS=("Animals")) NOT TS=("Humans")) |
| Combined | #15 | #1 AND #12 AND #13 AND #14 |

(d) Cochrane Central Register of Controlled Trials (CENTRAL)

| **Concept** | **Search number** | **Search terms** |
| --- | --- | --- |
| Population | #1 | MeSH descriptor:("Adolescent" OR "Young Adult") OR'young adult' OR 'adolesc*':ti,ab,kw OR adolescence:ti,ab,kw OR adolescent:ti,ab,kw OR adolescents:ti,ab,kw OR 'aya':ti,ab,kw OR 'city college':ti,ab,kw OR college:ti,ab,kw OR 'college student':ti,ab,kw OR 'young adult':ti,ab,kw OR 'young adulthood':ti,ab,kw OR 'young people':ti,ab,kw OR 'young person':ti,ab,kw OR 'young women':ti,ab,kw OR 'young men':ti,ab,kw OR 'school*':ti,ab,kw OR 'boy':ti,ab,kw OR 'boys':ti,ab,kw OR 'girl*':ti,ab,kw OR 'youth*':ti,ab,kw OR 'young*':ti,ab,kw OR 'student*':ti,ab,kw OR 'college students':ti,ab,kw OR 'community college*':ti,ab,kw OR 'university':ti,ab,kw OR 'eighth grade*':ti,ab,kw OR 'eleventh grade*':ti,ab,kw OR freshman:ti,ab,kw OR freshmen:ti,ab,kw OR 'grade 10':ti,ab,kw OR 'grade 11':ti,ab,kw OR 'grade 12':ti,ab,kw OR 'grade 7':ti,ab,kw OR 'grade 8':ti,ab,kw OR 'grade 9':ti,ab,kw OR 'grade eight':ti,ab,kw OR 'grade eleven':ti,ab,kw OR 'grade nine':ti,ab,kw OR 'grade seven':ti,ab,kw OR 'grade ten':ti,ab,kw OR 'grade twelve':ti,ab,kw OR 'high school':ti,ab,kw OR 'high-school':ti,ab,kw OR 'high school students':ti,ab,kw OR 'junior college':ti,ab,kw OR 'junior high':ti,ab,kw OR 'junior*':ti,ab,kw OR 'middle school':ti,ab,kw OR 'ninth grade*':ti,ab,kw OR 'senior high school':ti,ab,kw OR 'senior*':ti,ab,kw OR 'seventh grade*':ti,ab,kw OR sophomores:ti,ab,kw OR 'technical college':ti,ab,kw OR 'teen*':ti,ab,kw OR 'tenth grade*':ti,ab,kw OR 'two-year college':ti,ab,kw OR 'twelfth grade*':ti,ab,kw |
| Intervention | #2 | mhealth':ti,ab,kw OR 'm-health':ti,ab,kw OR 'ehealth':ti,ab,kw OR 'e-health':ti,ab,kw OR 'electronic health*':ti,ab,kw OR 'e-portal':ti,ab,kw OR 'eportal':ti,ab,kw OR 'e-consultation':ti,ab,kw OR 'remote consultation':ti,ab,kw OR 'e-care':ti,ab,kw OR 'etherap*':ti,ab,kw OR 'e therap*':ti,ab,kw OR 'ehealth literacy':ti,ab,kw OR 'e-literacy':ti,ab,kw |
|  | #3 | MeSH descriptor:(internet' OR 'internet-based intervention') OR 'web-based':ti,ab,kw OR 'internet-based':ti,ab,kw OR 'internet':ti,ab,kw OR 'web':ti,ab,kw OR 'webs':ti,ab,kw OR 'website*':ti,ab,kw OR 'online':ti,ab,kw OR 'on-line communication':ti,ab,kw OR 'online communication':ti,ab,kw OR 'social network*':ti,ab,kw |
|  | #4 | MeSH descriptor:('video games' OR 'games, experimental' OR 'games, recreational' OR 'exergaming' OR 'gamification') OR 'game*':ti,ab,kw OR 'gaming':ti,ab,kw OR 'interactive video*':ti,ab,kw OR 'virtual reality':ti,ab,kw OR 'board games':ti,ab,kw OR 'tabletop games':ti,ab,kw OR 'go game':ti,ab,kw OR 'videogame*':ti,ab,kw OR 'video game*':ti,ab,kw OR 'electronic game*':ti,ab,kw OR 'computer game*':ti,ab,kw OR 'online game*':ti,ab,kw OR 'internet game*':ti,ab,kw OR 'online gaming':ti,ab,kw OR 'exergame*':ti,ab,kw OR 'exergaming':ti,ab,kw OR 'serious game*':ti,ab,kw OR 'serious gaming':ti,ab,kw OR 'gamification':ti,ab,kw |
|  | #5 | MeSH descriptor:(mass media' OR 'multimedia') OR 'forum*':ti,ab,kw OR 'multi-media':ti,ab,kw OR 'multimedia':ti,ab,kw OR 'mass media':ti,ab,kw |
|  | #6 | MeSH descriptor:(text messaging) OR 'text messag*':ti,ab,kw OR 'short message service':ti,ab,kw OR 'sms':ti,ab,kw OR 'instant messag*':ti,ab,kw OR 'mms':ti,ab,kw OR 'text':ti,ab,kw OR 'messag*':ti,ab,kw |
|  | #7 | MeSH descriptor:(electronic mail) OR 'email*':ti,ab,kw OR 'e mail*':ti,ab,kw OR 'electronic mail*':ti,ab,kw |
|  | #8 | MeSH descriptor:('computers' OR 'computers, handheld' OR 'computer-assisted instruction' OR 'computer system*' OR 'microcomputers' OR 'cd rom') OR 'computer*':ti,ab,kw OR 'tablet*':ti,ab,kw OR 'handheld':ti,ab,kw OR 'technology':ti,ab,kw OR 'electronic tablet*':ti,ab,kw |
|  | #9 | MeSH descriptor:(telemedicine' OR 'telerehabilitation' OR 'cell phone' OR 'telephone' OR 'smartphone') OR 'mobile phone':ti,ab,kw OR 'mobile telephone*':ti,ab,kw OR 'smartphone*':ti,ab,kw OR 'telephone':ti,ab,kw OR 'phone':ti,ab,kw OR 'cell*':ti,ab,kw OR 'mobile device*':ti,ab,kw OR 'telemedicine':ti,ab,kw OR 'tele-medicine':ti,ab,kw OR 'telecare':ti,ab,kw OR 'tele-care':ti,ab,kw OR 'telehomecare':ti,ab,kw OR 'tele-homecare':ti,ab,kw OR 'telemonitoring':ti,ab,kw OR 'tele-monitoring':ti,ab,kw OR 'telehealth*':ti,ab,kw OR 'tele-health*':ti,ab,kw OR 'telecommunications':ti,ab,kw OR 'teletherap*':ti,ab,kw OR 'tele-rehabilitation':ti,ab,kw OR 'telerehabilitation':ti,ab,kw OR 'technological skill*':ti,ab,kw OR 'technology competenc*':ti,ab,kw OR 'technology skill*':ti,ab,kw OR 'telehealth competenc*':ti,ab,kw OR 'telemedicine skill*':ti,ab,kw OR 'telemedicine competenc*':ti,ab,kw OR 'android*':ti,ab,kw OR 'ipad*':ti,ab,kw OR 'iphone*':ti,ab,kw OR 'ipod*':ti,ab,kw OR 'ios':ti,ab,kw |
|  | #10 | MeSH descriptor:('communications media' OR 'wireless technology' OR 'internet' OR 'social media' OR 'mobile applications') OR 'mobile application*':ti,ab,kw OR 'mobile':ti,ab,kw OR 'mobile health application':ti,ab,kw OR 'app':ti,ab,kw OR 'apps':ti,ab,kw OR 'social media':ti,ab,kw OR 'media competenc*':ti,ab,kw OR 'media skill*':ti,ab,kw OR 'media literacy*':ti,ab,kw OR 'whatsapp':ti,ab,kw OR 'facebook':ti,ab,kw OR 'grindr':ti,ab,kw OR 'telegram':ti,ab,kw OR 'tiktok':ti,ab,kw OR 'geosocial':ti,ab,kw OR 'mxit':ti,ab,kw OR 'twitter':ti,ab,kw OR 'tweet':ti,ab,kw OR 'program*':ti,ab,kw OR 'jack'd':ti,ab,kw OR 'wechat':ti,ab,kw OR 'blued':ti,ab,kw OR 'chat room*':ti,ab,kw OR 'chatroom*':ti,ab,kw OR 'pda':ti,ab,kw |
|  | #11 | MeSH descriptor:(digital health' OR 'digital technology' OR 'artificial intelligence' OR 'robotics' OR 'machine learning' OR 'virtual reality') OR 'digital health':ti,ab,kw OR 'digital technology':ti,ab,kw OR 'digital intervention':ti,ab,kw OR 'digital media':ti,ab,kw OR 'digital assistant*':ti,ab,kw OR 'digital health literacy':ti,ab,kw OR 'digital literac*':ti,ab,kw OR 'digital competenc*':ti,ab,kw OR 'digital skill*':ti,ab,kw OR 'artificial intelligence':ti,ab,kw OR 'robotics':ti,ab,kw OR 'machine learning':ti,ab,kw OR 'virtual reality':ti,ab,kw |
|  | #12 | #2 OR #3 OR #4 OR #5 OR #6 OR #7 OR #8 OR #9 OR #10 OR #11 |
| Outcome | #13 | MeSH descriptor:('condoms' OR 'safe sex' OR 'unsafe sex' OR 'coitus' OR 'sexual partners' OR 'sexual behavior' OR 'risk-taking') OR 'condom*':ti,ab,kw OR 'condom use self-efficacy':ti,ab,kw OR 'condomless sex':ti,ab,kw OR 'condom use':ti,ab,kw OR 'condom compliance':ti,ab,kw OR 'condom behavior':ti,ab,kw OR ('sex':ti,ab,kw AND ('risk*':ti,ab,kw OR 'work*':ti,ab,kw OR 'behavior*':ti,ab,kw)) OR 'risk behavior*':ti,ab,kw OR 'risky behavior*':ti,ab,kw OR 'risk-taking':ti,ab,kw OR 'safe* sex*':ti,ab,kw OR 'unsafe* sex*':ti,ab,kw OR 'protected sex*':ti,ab,kw OR 'protected intercourse':ti,ab,kw OR 'unprotected sex*':ti,ab,kw OR 'unprotected intercourse':ti,ab,kw OR 'coitus':ti,ab,kw OR 'sexual partner*':ti,ab,kw OR 'sex partner*':ti,ab,kw OR 'multiple sexual partner*':ti,ab,kw OR 'multiple sex partner*':ti,ab,kw OR 'multiple partner*':ti,ab,kw OR 'sexually transmitted':ti,ab,kw OR 'stis':ti,ab,kw OR 'sti':ti,ab,kw OR 'stds':ti,ab,kw OR 'std':ti,ab,kw OR 'chlamydia':ti,ab,kw OR 'gonorrhea':ti,ab,kw OR 'syphilis':ti,ab,kw OR 'human immunodeficiency virus*':ti,ab,kw OR 'human immune deficiency virus*':ti,ab,kw OR 'hiv':ti,ab,kw OR 'aids':ti,ab,kw OR 'acquired immunodeficiency syndrome':ti,ab,kw OR 'acquired immune deficiency syndrome':ti,ab,kw |
| Study design | #14 | MeSH descriptor:("Clinical Trials as Topic") OR ("randomized controlled trial" OR "controlled clinical trial" OR "randomized" OR "placebo" OR "randomly" OR "trial"):ti,ab,kw |
| Combined | #15 | #1 AND #12 AND #13 AND #14 in Trials |
