# Supplementary material for: Effects of Digital Health Interventions to Promote Safer Sex Behaviors Among Youth: Systematic Review and Bayesian Network Meta-Analysis
Source: J Med Internet Res. 2026 Feb 4;28:e87071. doi: 10.2196/87071 (PMC12871581; doi:10.2196/87071)
Supplement: Multimedia Appendix 2 [file jmir-v28-e87071-s002.docx]

Multimedia Appendix 2. Reference list of excluded studies

This appendix lists excluded studies and the primary reason for exclusion. References are numbered within this appendix starting at 1.

| **No.** | **Primary reason for exclusion** | **Abbreviated reference** |
| --- | --- | --- |
| 1 | Intervention | J C Manderscheid. Health education and AIDS. A randomized controlled trial. 1992. |
| 2 | Intervention | Angela D. Bryan. Increasing Condom Use: Evaluation of a Theory-Based Intervention to Prevent Sexually Transmitted Diseases in Young Women. 1996. |
| 3 | Study design | John Noell. Development and Evaluation of a Sexual Decision-Making and Social Skills Program: "The Choice Is Yours-Preventing HIV/STDs". 1997. |
| 4 | Population | M.T.Mbizvo. Effects of a randomized health education intervention on aspects of reproductive health knowledge and reported behaviour among adolescents in Zimbabwe. 1997. |
| 5 | Other | Mary L. Kamb. Efficacy of Risk-Reduction Counseling to Prevent Human Immunodeficiency Virus and Sexually Transmitted Diseases a Randomized Controlled Trial. 1998. |
| 6 | Population | Seth C. Kalichman. Effectiveness of a Video-Based Motivational Skills-Building HIV Risk-Reduction Intervention for Inner-City African American Men. 1999. |
| 7 | Intervention | BONITA F. STANTON. Parental underestimates of adolescent risk behavior: A randomized, controlled trial of a parental monitoring intervention. 2000. |
| 8 | Population | Michael P. Carey. Using Information, Motivational Enhancement, and Skills Training to Reduce the Risk of HIV Infection for Low-Income UrbanWomen: A Second Randomized Clinical Trial. 2000. |
| 9 | Intervention | Karin Coyle. Safer Choices: Reducing Teen Pregnancy, HIV, and STDs. 2001. |
| 10 | Intervention | Lydia A. Shrier. Randomized Controlled Trial of a Safer Sex Intervention for High-Risk Adolescent Girls. 2001. |
| 11 | Population | Regina P. Lederman. The Parent-Adolescent Relationship Education (PARE) Program: A Curriculum for Prevention of STDs and Pregnancy in Middle School Youth. 2003. |
| 12 | Population; Intervention | Karin K. Coyle. Draw the Line/Respect the Line: A Randomized Trial of a Middle School Intervention to Reduce Sexual Risk Behaviors. 2004. |
| 13 | Study design | F. Caron. Evaluation of a theoretically based AIDS/STD peer education program on postponing sexual intercourse and on condom use among adolescents attending high school. 2004. |
| 14 | Intervention | OMOTAYO O. BOLU. Is HIV/Sexually Transmitted Disease Prevention Counseling Effective Among Vulnerable Populations? A Subset Analysis of Data Collected for a Randomized, Controlled Trial Evaluating Counseling Efficacy (Project RESPECT). 2004. |
| 15 | Population; Intervention | DOUGLAS B. KIRBY. The “Safer Choices” Intervention: Its Impact on the Sexual Behaviors of Different Subgroups of High School Students. 2004. |
| 16 | Intervention | Shamagonam James. The impact of an HIV and AIDS life skills program on secondary school students in Kwazulu-Natal, South Africa. 2006. |
| 17 | Intervention | Geoffrey T. Fong. Remembering the Message: The Use of a Reminder Cue to Increase Condom Use Following a Safer Sex Intervention. 2006. |
| 18 | Study design | Anthony J. Roberto. The effects of a computer-based pregnancy, STD, and HIV prevention intervention: A nine-school trial. 2007. |
| 19 | Population | Joseph F. Picciano. Lowering Obstacles to HIV Prevention Services: Effects of a Brief, Telephone-Based Intervention Using Motivational Enhancement Therapy. 2007. |
| 20 | Study design | Alicia M. Helion. Influence of communicator's race on efficacy of an HIV/STD prevention intervention among African American and caucasian college students: Populations at risk across the lifespan: Program evaluations. 2008. |
| 21 | Intervention | Natasha Slesnick. The impact of an integrated treatment on HIV risk behavior among homeless youth: A randomized controlled trial. 2008. |
| 22 | Population; Intervention | Sibusiso Sifunda. The Effectiveness of a Peer-Led HIV/AIDS and STI Health Education Intervention for Prison Inmates in South Africa. 2008. |
| 23 | Population; Intervention | Leo Wilton. Efficacy of an HIV/STI Prevention Intervention for Black Men Who Have Sex with Men: Findings from the Many Men, Many Voices (3MV) Project. 2009. |
| 24 | Intervention | Elaine A. Borawski. Taking Be Proud! Be Responsible! to the Suburbs: A Replication Study. 2009. |
| 25 | Intervention | Jessica M. Sales. Differences Between Dual-Method and Non–Dual-Method Protection Use in a Sample of Young African American Women Residing in the Southeastern United States. 2010. |
| 26 | Population; Intervention | X Chen. Effects on condom use of an HIV prevention programme 36 months postintervention: a cluster randomized controlled trial among Bahamian youth. 2010. |
| 27 | Intervention | Jessica M. Sales. Efficacy of an HIV Prevention Program Among African American Female Adolescents Reporting High Depressive Symptomatology. 2010. |
| 28 | Intervention | M. Margaret Dolcini. Project ORE: A Friendship-Based Intervention to Prevent HIV/STI in Urban African American Adolescent Females. 2010. |
| 29 | Intervention | B. R. Simon ROSSER. Reducing HIV risk behavior of men who have sex with men through persuasive computing: results of the Men's INTernet Study-II. 2010. |
| 30 | Population | Kelly M. Carpenter. Efficacy of a Web-Based Intervention to Reduce Sexual Risk in Men Who Have Sex with Men. 2010. |
| 31 | Population | John B. Jemmott III. Efficacy of a Theory-Based Abstinence-Only Intervention over 24 Months: A Randomized Controlled Trial with Young Adolescents. 2010. |
| 32 | Population | E. James Essien. Effectiveness of a video-based motivational skills-building HIV risk-reduction intervention for female military personnel. 2011. |
| 33 | Population | J. Gold. A randomised controlled trial using mobile advertising to promote safer sex and sun safety to young people. 2011. |
| 34 | Population | Seth M. Noar. Using computer technology for HIV prevention among African-Americans: development of a tailored information program for safer sex (TIPSS). 2011. |
| 35 | Intervention | Angela A. Robertson. The Healthy Teen Girls Project: Comparison of Health Education and STD Risk Reduction Intervention for Incarcerated Adolescent Females. 2011. |
| 36 | Intervention | Xiaoming Li. Effect of social cognitive theory-based HIV education prevention program among high school students in Nanjing, China. 2011. |
| 37 | Intervention | Gina M. Wingood. Efficacy of a Health Educator–Delivered HIV Prevention Intervention for Latina Women: A Randomized Controlled Trial. 2011. |
| 38 | Intervention | Mary Jane Rotheram-Borus. Reductions in sexually transmitted infections associated with popular opinion leaders in China in a randomised controlled trial. 2011. |
| 39 | Population | C. Mathews. Effects of the SATZ teacher-led school HIV prevention programmes on adolescent sexual behaviour: cluster randomised controlled trials in three sub-Saharan African sites. 2012. |
| 40 | Intervention | Katharine A. Atwood. Reducing sexual risk-taking behaviors among adolescents who engage in transactional sex in post-conflict Liberia. 2012. |
| 41 | Population | Christine M. Markham. Sexual Risk Avoidance and Sexual Risk Reduction Interventions For Middle School Youth: A Randomized Controlled Trial. 2012. |
| 42 | Population | Sabina Hirshfield. An Online Randomized Controlled Trial Evaluating HIV Prevention Digital Media Interventions for Men Who Have Sex with Men. 2012. |
| 43 | Population | Chun Hao. A Randomized Controlled Trial to Evaluate the Relative Efficacy of Enhanced Versus Standard Voluntary Counseling and Testing on Promoting Condom Use among Men Who Have Sex with Men in China. 2012. |
| 44 | Population | Charles H. Klein. WiLLOW: Reaching HIV-Positive African-American Women Through a Computer-Delivered Intervention. 2013. |
| 45 | Intervention | Stephen B. Kennedy. Effectiveness of a Brief Condom Promotion Program in Reducing Risky Sexual Behaviors among African American Males. 2013. |
| 46 | Population | Laura Widman. Do safer sex self-efficacy, attitudes toward condoms, and HIV transmission risk beliefs differ among men who have sex with men, heterosexual men, and women living with HIV?. 2013. |
| 47 | Study design | Arik V. Marcell. Effectiveness of a Brief Curriculum to Promote Condom and Health Care Use Among Out-of-School Young Adult Males. 2013. |
| 48 | Study design | Michael Hennessy. Safer Sex Media Messages and Adolescent Sexual Behavior: 3-Year Follow-Up Results from Project iMPPACS. 2013. |
| 49 | Intervention | Jesús Sánchez. Project Salud: Efficacy of a community-based HIV prevention intervention for Hispanic migrant workers in south Florida. 2013. |
| 50 | Intervention | Ralph J. DiClemente. Efficacy of a Telephone-Delivered Sexually Transmitted Infection/Human Immunodeficiency Virus Prevention Maintenance Intervention for Adolescents a Randomized Clinical Trial. 2014. |
| 51 | Population; Outcome | Thomas A. ODENY. Effect of Text Messaging to Deter Early Resumption of Sexual Activity after Male Circumcision for HIV Prevention: A Randomized Controlled Trial. 2014. |
| 52 | Intervention | Xiaoming Li. Efficacy of theory-based hiv behavioral prevention among rural-to-urban migrants in china: A randomized controlled trial. 2014. |
| 53 | Intervention | Alexandra M. Minnis. Yo Puedo - a conditional cash transfer and life skills intervention to promote adolescent sexual health: results of a randomized feasibility study in San Francisco. 2014. |
| 54 | Outcome | Marvin E. Belzer. The Use of Cell Phone Support for Non-adherent HIV-Infected Youth and Young Adults: An Initial Randomized and Controlled Intervention Trial. 2014. |
| 55 | Outcome | Megan E. Patrick. Web-based intervention to change perceived norms of college student alcohol use and sexual behavior on Spring Break. 2014. |
| 56 | Outcome | Melissa A. Lewis. Randomized Controlled Trial of a Web-Delivered Personalized Normative Feedback Intervention to Reduce Alcohol-Related Risky Sexual Behavior among College Students. 2014. |
| 57 | Intervention | Jennifer A Wagman. Effectiveness of an integrated intimate partner violence and HIV prevention intervention in Rakai, Uganda: analysis of an intervention in an existing cluster randomised cohort. 2015. |
| 58 | Study design | Corina Lelutiu-Weinberger. Feasibility, Acceptability, and Preliminary Efficacy of a Live-Chat Social Media Intervention to Reduce HIV Risk Among Young Men Who Have Sex with Men. 2015. |
| 59 | Population | Douglas W. Billings. A Randomized Trial to Evaluate the Efficacy of a Web-Based HIV Behavioral Intervention for High-Risk African American Women. 2015. |
| 60 | Population | Vivian F. Go. Efficacy of a Multi-level Intervention to Reduce Injecting and Sexual Risk Behaviors among HIV-Infected People Who Inject Drugs in Vietnam: A Four-Arm Randomized Controlled Trial. 2015. |
| 61 | Population | Sean D. Young. The HOPE Social Media Intervention for Global HIV Prevention: A Cluster Randomized Controlled Trial in Peru. 2015. |
| 62 | Intervention | Michael Mason. Peer Network Counseling with Urban Adolescents: A Randomized Controlled Trial with Moderate Substance Users. 2015. |
| 63 | Intervention | Colleen A. Redding. Randomized trial outcomes of a TTM-tailored condom use and smoking intervention in urban adolescent females. 2015. |
| 64 | Outcome | Michele L. Ybarra. A Randomized Controlled Trial to Increase HIV Preventive Information, Motivation, and Behavioral Skills in Ugandan Adolescents. 2015. |
| 65 | Intervention | Sarah Walsh. Effects of a Sexual Risk Reduction Program for African-American Adolescents on Social Cognitive Antecedents of Behavior Change. 2015. |
| 66 | Population | Brian Goesling. Impacts of an Enhanced Family Health and Sexuality Module of the HealthTeacher Middle School Curriculum: A Cluster Randomized Trial. 2016. |
| 67 | Population | Joel Milam. Randomized Controlled Trial of an Internet Application to Reduce HIV Transmission Behavior Among HIV Infected Men Who have Sex with Men. 2016. |
| 68 | Population | Z. Harry Piotrowski,. Evaluation of the Be the Exception Sixth-Grade Program in Rural Communities to Delay the Onset of Sexual Behavior. 2016. |
| 69 | Population | Susan C. Potter. It’s Your Game. . .Keep It Real in South Carolina: A Group Randomized Trial Evaluating the Replication of an Evidence-Based Adolescent Pregnancy and Sexually Transmitted Infection Prevention Program. 2016. |
| 70 | Population | Nina B. Baltierra. More than just tracking time: complex measures of user engagement with an internet-based health promotion intervention. 2016. |
| 71 | Population | David S. Festinger. Examining the efficacy of a computer facilitated HIV prevention tool in drug court. 2016. |
| 72 | Population | M. Isabel Fernandez. A Randomized Controlled Trial of POWER: An Internet-Based HIV Prevention Intervention for Black Bisexual Men. 2016. |
| 73 | Intervention | Meredith Kelsey. Replicating Reducing the Risk: 12-Month Impacts of a Cluster Randomized Controlled Trial. 2016. |
| 74 | Population | Lynn E Fiellin. The design and implementation of a randomized controlled trial of a risk reduction and human immunodeficiency virus prevention videogame intervention in minority adolescents: PlayForward: Elm City Stories. 2016. |
| 75 | Intervention | Amy J. Starosta. Safer sex in a digital world: A web-based motivational enhancement intervention to increase condom use among college women. 2016. |
| 76 | Intervention | Jennifer Thurheimer. Efficacy of the READY-Girls Program on General RiskTaking Behaviors, Condom Use, and Sexually Transmitted Infections Among Young Adolescent Females with Type 1 Diabetes. 2016. |
| 77 | Intervention | Catherine Mathews. Effects of PREPARE, a Multi-component, School-Based HIV and Intimate Partner Violence (IPV) Prevention Programme on Adolescent Sexual Risk Behaviour and IPV: Cluster Randomised Controlled Trial. 2016. |
| 78 | Intervention | Tamara Vehige. Healthy Futures Program and Adolescent Sexual Behaviors in 3 Massachusetts Cities: A Randomized Controlled Trial. 2016. |
| 79 | Intervention | Eric Jenner. Impact of an Intervention Designed to Reduce Sexual Health Risk Behaviors of African American Adolescents: Results of a Randomized Controlled Trial. 2016. |
| 80 | Intervention | MA Gold. A Randomized Controlled Trial Comparing Computer-Assisted Motivational Intervention to Didactic Educational Counseling to Reduce Unprotected Sex in Female Adolescents. 2016. |
| 81 | Intervention | Meredith Kelsey. Replicating ¡Cuídate!: 6-Month Impact Findings of a Randomized Controlled Trial. 2016. |
| 82 | No data available | Pamela J. Murray. Evaluation of Video Intervention on Sex-Related Psychosocial and Behavioral Outcomes in a Randomized Controlled Trial of Female Adolescents. 2016. |
| 83 | No data available | Ronald G Thompson. Effects of a smartphone application plus BMI in reducing substance and sexual risk among homeless young adults. 2017. |
| 84 | Study design | Benjamin B. Strauss. Exploring Patterns of Awareness and Use of HIV Pre-Exposure Prophylaxis Among Young Men Who Have Sex with Men. 2017. |
| 85 | Population | Elizabeth King. Mobile Text Messaging to Improve Medication Adherence and Viral Load in a Vulnerable Canadian Population Living with Human Immunodeficiency Virus: A Repeated Measures Study. 2017. |
| 86 | Population | Renee Garett. Ethical Issues in Using Social Media to Deliver an HIV Prevention Intervention: Results from the HOPE Peru Study. 2017. |
| 87 | Intervention | Elena Salmoirago-Blotcher. Phone-delivered mindfulness training to promote medication adherence and reduce sexual risk behavior among persons living with HIV: design and methods. 2017. |
| 88 | Intervention | Mee Lian Wong. Randomized controlled trial of abstinence and safer sex intervention for adolescents in Singapore: 6-month follow-up. 2017. |
| 89 | Intervention | Svetlana V. Doubova. Effects of an internet-based educational intervention to prevent high-risk sexual behavior in Mexican adolescents. 2017. |
| 90 | Population | Tracy Marie Scull. Using media literacy education for adolescent sexual health promotion in middle school: Randomized control trial of Media Aware. 2018. |
| 91 | Population | Hussein Haruna. Improving Sexual Health Education Programs for Adolescent Students through Game-Based Learning and Gamification. 2018. |
| 92 | Population | Eileen V. Pitpitan. Factors associated with program effectiveness in the implementation of a sexual risk reduction intervention for female sex workers across Mexico: Results from a randomized trial. 2018. |
| 93 | Population | Katerina A. Christopoulos. A Randomized Controlled Trial of a Text Messaging Intervention to Promote Virologic Suppression and Retention in Care in an Urban Safety-Net Human Immunodeficiency Virus Clinic: The Connect4Care Trial. 2018. |
| 94 | Population | Lisette Schutte. Effect Evaluation of a Web-Based Coaching Intervention to Support Implementation of Sex Education Among Secondary School Teachers: Randomized Controlled Trial. 2018. |
| 95 | Population | Christopher D. Houck. Sexual Risk Outcomes of an Emotion Regulation Intervention for At-Risk Early Adolescents. 2018. |
| 96 | Intervention | Robert Garofalo. Efficacy of an Empowerment-Based, Group-Delivered HIV Prevention Intervention for Young Transgender Women. 2018. |
| 97 | Intervention | Stephen B. Kennedy. Preliminary Impacts of an HIV Prevention Program Targeting Out-of School Youth in Postconflict Liberia. 2018. |
| 98 | Intervention | G. Anita Heeren. Health-Promotion Intervention Increases Self-Reported Physical Activity in Sub-Saharan African University Students: A Randomized Controlled Pilot Study. 2018. |
| 99 | Intervention | Richard A. Crosby. Efficacy of a Clinic-Based Safer Sex Program for Human Immunodeficiency Virus–Uninfected and Human Immunodeficiency Virus–Infected Young Black Men Who Have Sex with Men: A Randomized Controlled Trial. 2018. |
| 100 | Intervention | Richard A. Crosby. Promoting positive condom use experiences among young black MSM: a randomized controlled trial of a brief, clinic-based intervention. 2018. |
| 101 | Intervention | Laura Widman. Sexual Assertiveness Skills and Sexual Decision-Making in Adolescent Girls: Randomized Controlled Trial of an Online Program. 2018. |
| 102 | Intervention | Julie S. Downs. Video Intervention to Increase Perceived Self-Efficacy for Condom Use in a Randomized Controlled Trial of Female Adolescents. 2018. |
| 103 | Intervention | Julie S. Downs. Video Intervention to Increase Perceived Self-Efficacy for Condom Use in a Randomized Controlled Trial of Female Adolescents. 2018. |
| 104 | Outcome | Ona McCarthy. A randomized controlled trial of an intervention delivered by mobile phone app instant messaging to increase the acceptability of effective contraception among young people in Tajikistan. 2018. |
| 105 | Outcome | Michele L. Ybarra. The Effect of a Text Messaging Based HIV Prevention Program on Sexual Minority Male Youths: A National Evaluation of Information, Motivation and Behavioral Skills in a Randomized Controlled Trial of Guy2Guy. 2018. |
| 106 | Intervention | Monica O. Kuteesa. Feasibility of conducting HIV combination prevention interventions in fishing communities in Uganda: A pilot cluster randomised trial. 2019. |
| 107 | Intervention | Natawan Khumsaen. Feasibility and Acceptability of an HIV/AIDS Self-Management Education Program for HIV-positive Men who have Sex with Men in Thailand. 2019. |
| 108 | Study design | Louise A. Rohrbach. Effectiveness Evaluation of It’s Your Game: Keep It Real, a Middle School HIV/Sexually Transmitted Infection/Pregnancy Prevention Program. 2019. |
| 109 | Population | Kate Winskell. Interactive Narrative in a Mobile Health Behavioral Intervention (Tumaini): Theoretical Grounding and Structure of a Smartphone Game to Prevent HIV Among Young Africans. 2019. |
| 110 | Population | Emily A. Arnold. A Randomized Controlled Trial to Reduce HIV-Related Risk in African American Men who have Sex with Men and Women: The Bruthas Project. 2019. |
| 111 | Population | Melissa F. Peskin. Replication of It’s Your Game...Keep It Real! in Southeast Texas. 2019. |
| 112 | Population | Thomas L. Patterson. Impact of time perspectives on texting intervention to reduce HIV/ STI transmission among female sex workers in Tijuana and Ciudad Juarez, Mexico. 2019. |
| 113 | Population | Cathy J. Reback. Theory-based Text-Messaging to Reduce Methamphetamine Use and HIV Sexual Risk Behaviors among Men who have Sex with Men: Automated Unidirectional Delivery Outperforms Bidirectional Peer Interactive Delivery. 2019. |
| 114 | Population | Weibin Cheng. Online HIV prevention intervention on condomless sex among men who have sex with men: a web-based randomized controlled trial. 2019. |
| 115 | Outcome | Steven Paul Hafner. Sexual Health, STI and HIV Risk, and Risk Perceptions Among American Indian and Alaska Native Emerging Adults. 2019. |
| 116 | Population | Farideh Khalajabadi Farahani. The Effect of Theory-Based HIV/AIDS Educational Program on Preventive Behaviors Among Female Adolescents in Tehran: A Randomized Controlled Trial. 2020. |
| 117 | Population | Tarandeep Anand. Implementation and impact of a technology-based HIV riskreduction intervention among Thai men who have sex with men using ‘Vialogues’: A randomized controlled trial. 2020. |
| 118 | Intervention | Laurel P. Gibson. Mechanisms of action for empirically supported interventions to reduce adolescent sexual risk behavior: A randomized controlled trial. 2020. |
| 119 | Intervention | Fang Yu. Preventing HIV and HSV-2 through knowledge and attitudes: A replication study of a multicomponent community-based intervention in Zimbabwe. 2020. |
| 120 | Population | Parya Saberi. A Mobile Health App (WYZ) for Engagement in Care and Antiretroviral Therapy Adherence Among Youth and Young Adults Living With HIV: Single-Arm Pilot Intervention Study. 2021. |
| 121 | Intervention | Ash Philliber. The IN$clued Program: A Randomized Control Trial of an Effective Sex Education Program for Lesbian, Gay, Bisexual, Transgender, Queer, and Questioning Youths. 2021. |
| 122 | Outcome | Mandy J. Hill. Novel Use of Video Logs to Deliver Educational Interventions to Black Women for Disease Prevention. 2021. |
| 123 | Outcome | Jon Agley. HIV/AIDS and Substance Use Prevention for African American Young Adults: Field Evaluation of “Color it Real”. 2021. |
| 124 | No data available | Miguel Iván Gómez-Flores. Effect of a tele-nursing intervention on condom use in adolescents. 2022. |
| 125 | No data available | Jason Zucker. GET2PrEP3: RCT of PROVIDER MESSAGING to IMPROVE LINKAGE to HIV PREVENTION SERVICES. 2022. |
| 126 | Population; Outcome | Megan A. Lewis. Effectiveness of an Interactive, Highly Tailored “Video Doctor” Intervention to Suppress Viral Load and Retain Patients with HIV in Clinical Care: A Randomized Clinical Trial. 2022. |
| 127 | Population; Outcome | Craig Winston LeCroy. Guy Talk: A Gender-Specific Sexual Education Program to Reduce Sexual Risk Behaviors with High School Males. 2022. |
| 128 | Intervention | Mayra Gómez‐Lugo. Effects of a Sexual Risk‐Reduction Intervention for Teenagers: A Cluster‐Randomized Control Trial. 2022. |
| 129 | Intervention | Faith Ussery. HIV Incidence in Botswana Rural Communities with High Antiretroviral Treatment Coverage: Results from the Botswana Combination Prevention Project, 2013-2017. 2022. |
| 130 | Population; Outcome | Laura Whiteley. The Impact of a Mobile Gaming Intervention to Increase Adherence to Pre-exposure Prophylaxis. 2022. |
| 131 | Outcome | Staci J. Wendt. Impacts of Healthy U: A cluster-randomized control trial of a sexual health education app developed for justice-involved male youth. 2022. |
| 132 | Outcome | José L. Hernández-Torres. Use of Smartphones to Increase Safe Sexual Behavior in Youths at Risk for HIV. 2022. |
| 133 | Outcome | Jian Tang. Evaluation of an AIDS Educational Mobile Game (AIDS Fighter Health Defense) for Young Students to Improve AIDS-Related Knowledge, Stigma, and Attitude Linked to High-Risk Behaviors in China: Randomized Controlled Trial. 2022. |
| 134 | Outcome | David L. Wyrick. itMatters: Optimization of an online intervention to prevent sexually transmitted infections in college students. 2022. |
| 135 | Outcome | Maria Lohan. Effects of gender-transformative relationships and sexuality education to reduce adolescent pregnancy (the JACK trial): a cluster-randomised trial. 2022. |
| 136 | Outcome | Lynn Rew. Promoting Healthy Attitudes and Behaviors in Youth who Experience Homelessness: Results of a Longitudinal Intervention Study. 2022. |
| 137 | Population | Michael E. Newcomb. Biomedical and Behavioral Outcomes of 2GETHER: A Randomized Controlled Trial of a Telehealth HIV Prevention Program for Young Male Couples. 2023. |
| 138 | Population | Beatrice Aladin. The YGetIt? Program: A Mobile Application, PEEP, and Digital Comic Intervention to Improve HIV Care Outcomes for Young Adults. 2023. |
| 139 | Intervention | David M. Huebner. Effects of a Parent‐Focused HIV Prevention Intervention for Young Men Who have Sex with Men: A Pilot Randomized Clinical Trial. 2023. |
| 140 | Intervention | Eric Jenner. Randomized Trial of a Sexual Health Video Intervention for Black and Hispanic Adolescent Females. 2023. |
| 141 | Intervention | Niranjan S Karnik. Findings From the Step Up, Test Up Study of an Electronic Screening and Brief Intervention for Alcohol Misuse in Adolescents and Young Adults Presenting for HIV Testing: Randomized Controlled Efficacy Trial. 2023. |
| 142 | Outcome | Eric R. Pedersen. A Randomized Controlled Trial of an Online Alcohol and Sexual Risk Prevention Program for College Students Studying Abroad. 2023. |
| 143 | Population | M. C. Versloot-Swildens. Effectiveness of a Comprehensive School-Based Sex Education Program for Young Adolescents in the Netherlands. 2024. |
| 144 | Intervention | Arthur H. Owora. Promoting Healthy Adolescent Romantic Relationships: Results of a Multisite, Two-group Parallel Randomized Clinical Trial. 2024. |
| 145 | Intervention | Pamela J. Drake. Evaluating a Future-Oriented Positive Youth Development Intervention to Reduce Sexual Risk Among Highly Mobile Youth: Results and Challenges. 2024. |
| 146 | Outcome | Jad Sinno. Social Media and Online Dating Safety Practices by Adolescent Sexual and Gender Diverse Men: Mixed-Methods Findings from the SMART Study. 2024. |
| 147 | Outcome | Jason Zucker. Get2PrEP2—A Provider Messaging Strategy to Improve PrEP Uptake: A Randomized Controlled Quality Improvement Project. 2024. |
| 148 | Outcome | Oluwatosin Wuraola Akande. The effectiveness of an m-Health intervention on the sexual and reproductive health of in-school adolescents: a cluster randomized controlled trial in Nigeria. 2024. |
| 149 | Outcome | Tyra Pendergrass Boomer, M.E.M.. A Serious Video Game Targeting HIV Testing and Counseling: A Randomized Controlled Trial. 2024. |
| 150 | Outcome | Cathy J. Reback. Technology‐Based Interventions, with a Stepped Care Approach, for Reducing Sexual Risk Behaviors and Increasing PrEP Initiation Among Transgender and Gender Expansive Youth and Young Adults. 2024. |
| 151 | Outcome | Cathy J. Reback. Technology‐Based Interventions, with a Stepped Care Approach, for Reducing Sexual Risk Behaviors and Increasing PrEP Initiation Among Transgender and Gender Expansive Youth and Young Adults. 2024. |
| 152 | Study design | Katie M. Edwards. “It changed me for the better”: Feasibility, acceptability, and safety of an online program for sexual and gender minority youth to prevent alcohol use and teen dating violence. 2024. |
| 153 | Outcome | Birgit Wagner. Internet-Based Prevention of Re-Victimization for Youth with Care Experience (EMPOWER-YOUTH): Results of a Randomized Controlled Trial. 2024. |
| 154 | Intervention | Germany. In the Know: A Cluster Randomized Trial of an In-person Sexual Health Education Program Integrating Digital Technologies for Adolescents. 2024. |
| 155 | Intervention | Jordyn McCrimmon. Evaluation of a Brief Online Sexual Health Program for Adolescents: A Randomized Controlled Trial. 2024. |
| 156 | Intervention | Sheana S. Bull. Social Media–Delivered Sexual Health Intervention: A Cluster Randomized Controlled Trial. 2012. |
| 157 | Intervention | Sheana S. Bull. Social Media–Delivered Sexual Health Intervention: A Cluster Randomized Controlled Trial. 2012. |
| 158 | Duplicate record | Marı ́a Dolores Gil-Llario. Effectiveness of HIV prevention for women: what is working?. 2014. |
| 159 | Duplicate record | Marı ́a Dolores Gil-Llario. Effectiveness of HIV prevention for women: what is working?. 2014. |
| 160 | Duplicate record | Marı ́a Dolores Gil-Llario. Effectiveness of HIV prevention for women: what is working?. 2014. |
| 161 | Duplicate record | Marı ́a Dolores Gil-Llario. Effectiveness of HIV prevention for women: what is working?. 2014. |
| 162 | Outcome | Svetlana V. Doubova. Effects of an internet-based educational intervention to prevent high-risk sexual behavior in Mexican adolescents. 2017. |
| 163 | Outcome | Tyra Pendergrass. Videogame intervention to encourage HIV testing and counseling among adolescents. 2020. |
| 164 | Intervention | Tyrel J. Starks. Optimizing Individual HIV Testing and Counseling for Emerging Adult Sexual Minority Men (Aged 18 to 24) in Relationships: A Pilot Randomized Controlled Trial of Adjunct Communication Components. 2024. |
| 165 | Study design | Keshet Ronen. Facilitated WhatsApp Support Groups for Youth Living with HIV in Nairobi, Kenya: Single-Arm Pilot Intervention Study. 2023. |
| 166 | Population | Cosio David. Telephone-Administered Motivational Interviewing to Reduce Risky Sexual Behavior in HIV-Infected Rural Persons: A Pilot Randomized Clinical Trial. 2010. |
| 167 | Outcome | Joseph T. F. Lau. A pilot clustered randomized control trial evaluating the efficacy of a network-based HIV peer-education intervention targeting men who have sex with men in Hong Kong, China. 2013. |
| 168 | Intervention | Megan S. Dunbar. The SHAZ! Project: Results from a Pilot Randomized Trial of a Structural Intervention to Prevent HIV among Adolescent Women in Zimbabwe. 2014. |
| 169 | Study design | Angela Chia Chen. A Pilot, Web-Based HIV/STI Prevention Intervention Targeting At-Risk Mexican American Adolescents: Feasibility, Acceptability, and Lessons Learned. 2017. |
| 170 | Population | Matthew J. Mimiaga. A Pilot Randomized Controlled Trial of an Integrated In-person and Mobile Phone Delivered Counseling and Text Messaging Intervention to Reduce HIV Transmission Risk among Male Sex Workers in Chennai, India. 2017. |
| 171 | Population | Viraj V Patel. An Internet-Based, Peer-Delivered Messaging Intervention for HIV Testing and Condom Use Among Men Who Have Sex With Men in India (CHALO!): Pilot Randomized Comparative Trial. 2020. |
| 172 | Population | Kate Winskell. A Smartphone Game-Based Intervention (Tumaini) to Prevent HIV Among Young Africans: Pilot Randomized Controlled Trial. 2018. |
| 173 | Population | Wray, T. B.. Pilot Randomized Controlled Trial of Game Plan for PrEP: A Brief, Web and Text Message Intervention to Help Sexual Minority Men Adhere to PrEP and Reduce Their Alcohol Use. 2024. |
| 174 | Outcome | Wernette, G. T.. A Pilot Randomized Controlled Trial of a Computer-Delivered Brief Intervention for Substance Use and Risky Sex during Pregnancy. 2018. |
| 175 | Duplicate record | Hightow-Weidman, L.. Pilot study of a gamified, social networking app shows improvements in PrEP adherence among YMSM in the US. 2018. |
| 176 | Study design | Bogart, L. M.. A Pilot Test of Game Changers, a Social Network Intervention to Empower People with HIV to be Prevention Advocates in Uganda. 2020. |
| 177 | Outcome | Nelson, K. M.. The Young Men and Media Study: A Pilot Randomized Controlled Trial of a Community-Informed, Online HIV Prevention Intervention for 14-17-Year-Old Sexual Minority Male. 2022. |
| 178 | Study design | Dorcas Adedoja. MyPEEPS Mobile App for HIV Prevention Among Transmasculine Youth: Adaptation Through Community-Based Feedback and Usability Evaluation. 2024. |
| 179 | Intervention | Michele L. Ybarra. A Pilot RCT Evaluating InThistoGether, an mHealth HIV Prevention Program for Ugandan Youth. 2021. |
| 180 | Intervention | A. M. Bowen. Internet based HIV prevention research targeting rural MSM: Feasibility, acceptability, and preliminary efficacy. 2008. |
| 181 | Intervention | Larissa Jennings Mayo-Wilson. Microenterprise Intervention to Reduce Sexual Risk Behaviors and Increase Employment and HIV Preventive Practices Among Economically-Vulnerable African-American Young Adults (EMERGE): A Feasibility Randomized Clinical Trial. 2020. |
| 182 | Intervention | Michele L. Ybarra. Pilot RCT results of an mHealth HIV prevention program for sexual minority male adolescents. 2017. |
| 183 | Duplicate record | Caroline Free. Can text messages increase safer sex behaviours in young people? Intervention development and pilot randomised controlled trial. 2016. |
| 184 | Duplicate record | Caroline Free. Effectiveness of a behavioural intervention delivered by text messages (safetxt) on sexually transmitted reinfections in people aged 16-24 years: randomised controlled trial. 2022. |
| 185 | Study design | Sophie Alexander. Assessing the effectiveness of youth community based sexual and reproductive health interventions in Sierra Leone: Before and after the Ebola virus disease outbreak. 2017. |
| 186 | No data available | Megan C. Barry. “Stay strong! keep ya head up! move on! it gets better!!!!”: resilience processes in the healthMpowerment online intervention of young black gay, bisexual and other men who have sex with men. 2018. |
| 187 | Intervention | Yannine Estrada. eHealth Familias Unidas: Efficacy Trial of an Evidence-Based Intervention Adapted for Use on the Internet with Hispanic Families. 2019. |
| 188 | Intervention | Michael P. Carey. A Brief Clinic‐Based Intervention to Reduce Alcohol Misuse and Sexual Risk Behavior in Young Women: Results from an Exploratory Clinical Trial. 2020. |
| 189 | No data available | Dallas Swendeman. Optimal strategies to improve uptake of and adherence to HIV prevention among young people at risk for HIV acquisition in the USA (ATN 149): a randomised, controlled, factorial trial. 2024. |
| 190 | No data available | Patricia J. Kissinger. Internet Delivered Sexually Transmitted Infection and Teen Pregnancy Prevention Program: A Randomized Trial. 2023. |
| 191 | No data available | Schnall, Jia, Rodriguez. Efficacy of MyPEEPS Mobile, an HIV Prevention Intervention Using Mobile Technology, on Reducing Sexual Risk Among Same-Sex Attracted Adolescent Males A Randomized Clinical Trial. 2022. |
| 192 | No data available | Wipaporn Natalie Songtaweesin. Youth-friendly services and a mobile phone application to promote adherence to pre-exposure prophylaxis among adolescent men who have sex with men and transgender women at-risk for HIV in Thailand: a randomized control trial. 2020. |
| 193 | No data available | Levy Einav. The effects of psychological inoculation on condom use tendencies and barriers; a randomized controlled trial. 2021. |
| 194 | No data available | Rachel Jones. A Human Immunodeficiency Virus Posttest Video to Increase Condom Use Among Adolescent Emergency Department Patients. 2013. |
| 195 | No data available | Rachel JonesRachel Jones. A Randomized Controlled Trial of Soap Opera Videos Streamed to Smartphones to Reduce HIV Sex Risk in Young Urban African American Women. 2013. |
| 196 | No data available | Sharon Sznitman. Using Culturally Sensitive Media Messages to Reduce HIVassociated Sexual Behavior in High-risk African-American Adolescents: Results from a Randomized Trial. 2011. |
| 197 | No data available | Carinne Brody. A Mobile Intervention to Link Young Female Entertainment Workers in Cambodia to Health and Gender-Based Violence Services: Randomized Controlled Trial. 2001. |
| 198 | No data available | Sheana Bull. Effects of an Internet-Based Intervention for HIV Prevention: The Youthnet Trials. 2009. |
| 199 | No data available | Catherine A. Sanderson. Improving Condom Use Intentions and Behavior by Changing Perceived Partner Norms: An Evaluation of Condom Promotion Videos for College Students. 2007. |
| 200 | No data available | Ralph J. DiClemente. Rate of Decay in Proportion of Condom Protected Sex Acts among Adolescents Following Participation in an HIV Risk Reduction Intervention. 2013. |
| 201 | No data available | Carinne Brody. A Mobile Intervention to Link Young Female Entertainment Workers in Cambodia to Health and Gender-Based Violence Services: Randomized Controlled Trial. 2022. |
| 202 | No data available | Lisa B. Hightow‐Weidman. A Randomized Trial of an Online Risk Reduction Intervention for Young Black MSM. 2019. |
| 203 | No data available | German Neubaum. Let’s Blog About Health! Exploring the Persuasiveness of a Personal HIV Blog Compared to an Institutional HIV Website. 2015. |
| 204 | No data available | Catherine A. Sanderson. Role of Relationship Context in Influencing College Students' Responsiveness to HIV PreventionVideos. 1999. |
| 205 | No data available | Wai Han Sun. A Peer-Led, Social Media-Delivered, Safer Sex Intervention for Chinese College Students: Randomized Controlled Trial. 2017. |
| 206 | No data available | Fraukje E.F. Mevissen. Justify your love: Testing an online STI-risk communication intervention designed to promote condom use and STI-testing. 2011. |
| 207 | No data available | Lisa A. Marsch. Comparative Effectiveness of Web-Based vs. Educator-Delivered HIV Prevention for Adolescent Substance Users: A Randomized, Controlled Trial. 2015. |
| 208 | No data available | Susan M. Kiene. A Brief Individualized Computer-Delivered Sexual Risk Reduction Intervention Increases HIV/AIDS Preventive Behavior. 2006. |
| 209 | Outcome | David Cordova. Pilot Study of a Digital Health Intervention to Increase HIV and Sexually Transmitted Infection Testing Uptake and Reduce Condomless Sex and Substance Use Among Adolescents. 2025. |
| 210 | Outcome | Rebecca Hémono. Exposure and Engagement Drive Impact: Results from a Large-Scale Trial of a Digital Family Planning and Reproductive Health Intervention in Rwanda. 2025. |
