# Supplementary material for: Effects of Digital Health Interventions to Promote Safer Sex Behaviors Among Youth: Systematic Review and Bayesian Network Meta-Analysis
Source: J Med Internet Res. 2026 Feb 4;28:e87071. doi: 10.2196/87071 (PMC12871581; doi:10.2196/87071)
Supplement: Multimedia Appendix 3 [file jmir-v28-e87071-s003.docx]

**(1) Risk of bias graph. Review author’s judgement for each risk of bias item presented as percentages of all included studies**

**(2) Network plot of consistent condom use rates in youth following DHIs and NDIs**


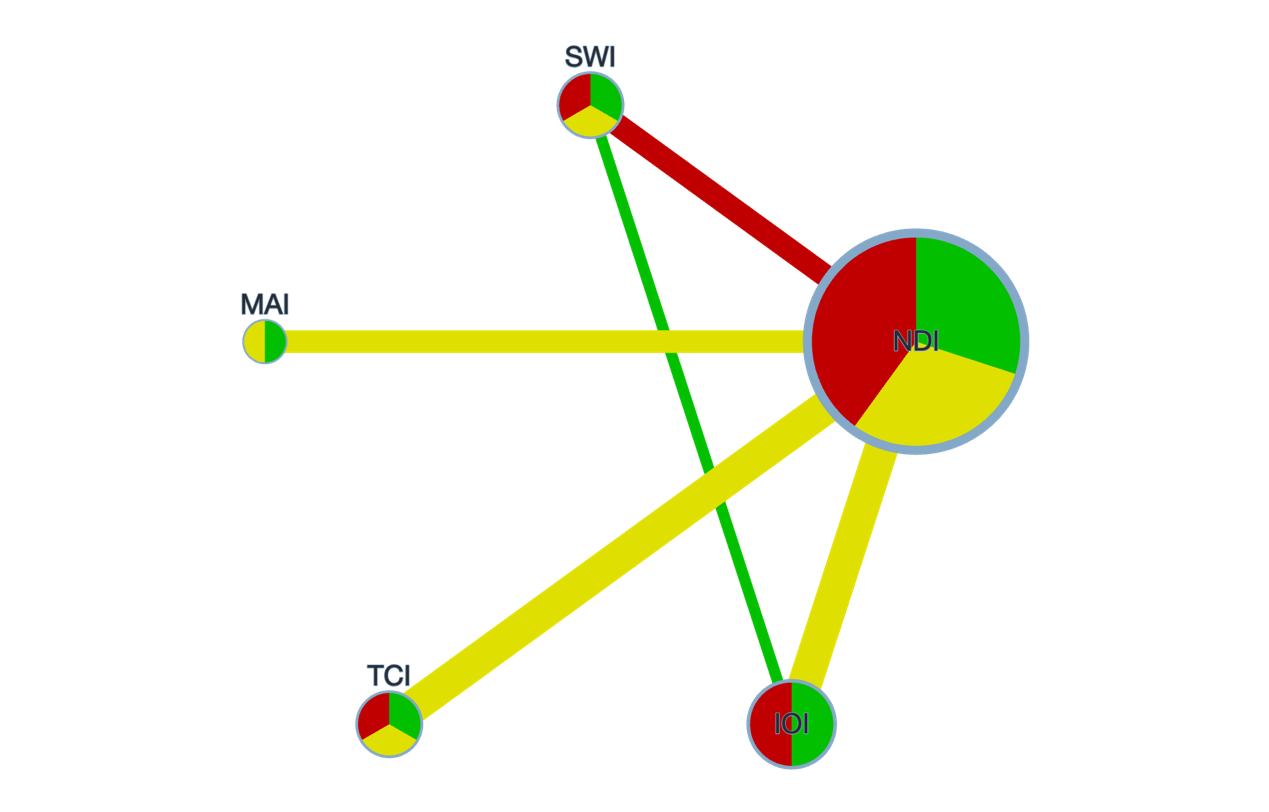


*Node size reflects the number of studies involving each intervention. Node color indicates the overall risk of bias (RoB) for studies involving that intervention. Edge width represents the number of direct comparisons between interventions. Edge color denotes the average RoB of the studies contributing to each comparison*

**(3) Contribution of risk of bias across comparisons of different interventions**


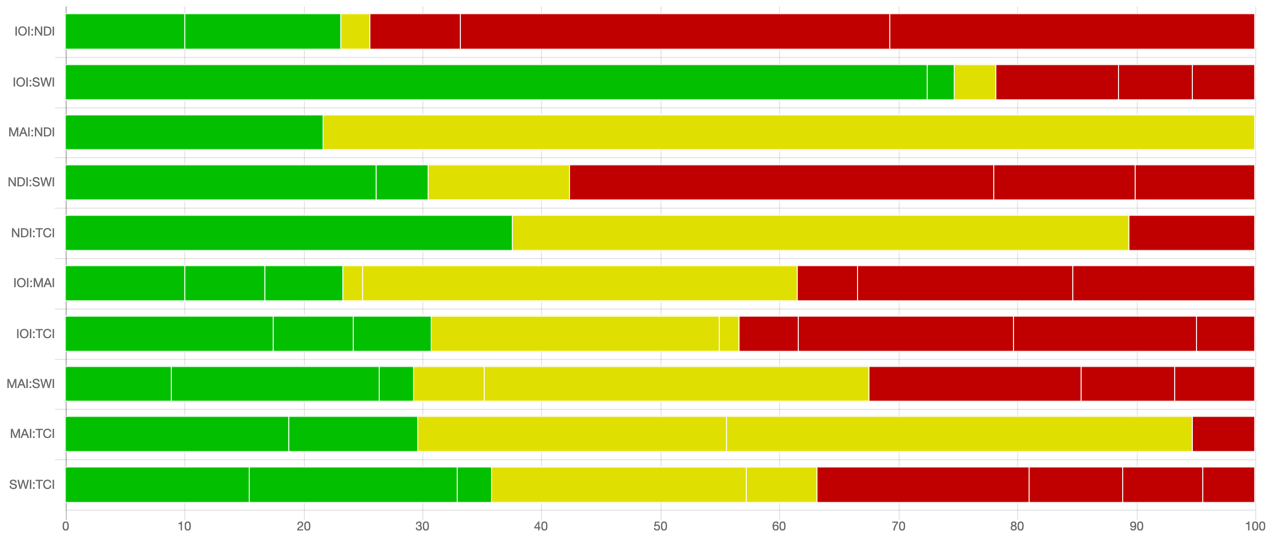


**(4) GRADE Summary of Findings Tables**

**Author(s): Yiran Zhu, Wenwen Peng**

**Condom use rate in the last sexual contact**

**Question:** [MAI] compared to [NDI] for [Condom use rate in the last sexual contact]

| **Certainty assessment** | | | | | | | **№ of patients** | | **Effect** | | **Certainty** | **Importance** |
| --- | --- | --- | --- | --- | --- | --- | --- | --- | --- | --- | --- | --- |
| **№ of studies** | **Study design** | **Risk of bias** | **Inconsistency** | **Indirectness** | **Imprecision** | **Other considerations** | **[MAI]** | **[NDI]** | **Relative (95% CI)** | **Absolute (95% CI)** |  |  |
| 1 | randomised trials | not serious | not serious | not serious | serious^a^ | none | 332/432 (76.9%) | 302/414 (72.9%) | **OR 1.23** (0.90 to 1.68) | **39 more per 1,000** (from 21 fewer to 90 more) | ⨁⨁⨁◯ Moderate^a^ |  |

**CI:** confidence interval; **OR:** odds ratio

#### Explanations

a. Downgraded one level for serious imprecision: the 95% CI (0.90–1.68) crosses the line of no effect and includes both little or no effect and a potentially important benefit, based on a single study with limited sample size.

**Question:** [IOI] compared to [NDI] for [Condom use rate in the last sexual contact]

| **Certainty assessment** | | | | | | | **№ of patients** | | **Effect** | | **Certainty** | **Importance** |
| --- | --- | --- | --- | --- | --- | --- | --- | --- | --- | --- | --- | --- |
| **№ of studies** | **Study design** | **Risk of bias** | **Inconsistency** | **Indirectness** | **Imprecision** | **Other considerations** | **[IOI]** | **[NDI]** | **Relative (95% CI)** | **Absolute (95% CI)** |  |  |
| 3 | randomised trials | not serious | not serious | not serious | very serious^a^ | none | 46/99 (46.5%) | 54/101 (53.5%) | **OR 0.78** (0.45 to 1.37) | **62 fewer per 1,000** (from 194 fewer to 77 more) | ⨁⨁◯◯ Low^a^ |  |

**CI:** confidence interval; **OR:** odds ratio

#### Explanations

a. Downgraded two levels for very serious imprecision: the 95% CI (0.45–1.37) is wide, crosses the line of no effect, and includes both important harm (upper bound >1.25) and large benefit, with a limited total sample size.

**Question:** [TCI] compared to [NDI] for [Condom use rate in the last sexual contact]

| **Certainty assessment** | | | | | | | **№ of patients** | | **Effect** | | **Certainty** | **Importance** |
| --- | --- | --- | --- | --- | --- | --- | --- | --- | --- | --- | --- | --- |
| **№ of studies** | **Study design** | **Risk of bias** | **Inconsistency** | **Indirectness** | **Imprecision** | **Other considerations** | **[TCI]** | **[NDI]** | **Relative (95% CI)** | **Absolute (95% CI)** |  |  |
| 3 | randomised trials | serious^a^ | not serious | not serious | not serious | none | 1085/3205 (33.9%) | 1000/3215 (31.1%) | **OR 1.13** (1.02 to 1.26) | **27 more per 1,000** (from 4 more to 52 more) | ⨁⨁⨁◯ Moderate^a^ |  |

**CI:** confidence interval; **OR:** odds ratio

#### Explanations

a. Deviations from the intended interventions；Missing outcome data；Measurement of the outcome；Selection of the reported result

**Consistent condom use**

**Question:** [IOI] compared to [SWI] for [Consistent condom use]

| **Certainty assessment** | | | | | | | **№ of patients** | | **Effect** | | **Certainty** | **Importance** |
| --- | --- | --- | --- | --- | --- | --- | --- | --- | --- | --- | --- | --- |
| **№ of studies** | **Study design** | **Risk of bias** | **Inconsistency** | **Indirectness** | **Imprecision** | **Other considerations** | **[IOI]** | **[SWI]** | **Relative (95% CI)** | **Absolute (95% CI)** |  |  |
| 1 | randomised trials | not serious | not serious | not serious | serious^a^ | none | 124/272 (45.6%) | 124/270 (45.9%) | **OR 0.99** (0.70 to 1.38) | **2 fewer per 1,000** (from 86 fewer to 80 more) | ⨁⨁⨁◯ Moderate^a^ |  |

**CI:** confidence interval; **OR:** odds ratio

#### Explanations

a. the 95% CI (0.70–1.38) crosses the line of no effect and includes both little or no effect and a potentially important effect in either direction, based on a single study with limited information size.

**Question:** [SWI] compared to [NDI] for [Consistent condom use]

| **Certainty assessment** | | | | | | | **№ of patients** | | **Effect** | | **Certainty** | **Importance** |
| --- | --- | --- | --- | --- | --- | --- | --- | --- | --- | --- | --- | --- |
| **№ of studies** | **Study design** | **Risk of bias** | **Inconsistency** | **Indirectness** | **Imprecision** | **Other considerations** | **[SWI]** | **[NDI]** | **Relative (95% CI)** | **Absolute (95% CI)** |  |  |
| 2 | randomised trials | very serious^a^ | not serious | not serious | very serious^b^ | none | 114/133 (85.7%) | 114/144 (79.2%) | **OR 2.06** (0.58 to 7.28) | **95 more per 1,000** (from 104 fewer to 173 more) | ⨁◯◯◯ Very low^a,b^ |  |

**CI:** confidence interval; **OR:** odds ratio

#### Explanations

a. Deviations from the intended interventions; Missing outcome data; Selection of the reported result

b. Downgraded two levels for very serious imprecision: the 95% CI (0.58–7.28) is extremely wide and includes both important harm and very large benefit, with a small total sample size.

**Question:** [IOI] compared to [NDI] for [Consistent condom use]

| **Certainty assessment** | | | | | | | **№ of patients** | | **Effect** | | **Certainty** | **Importance** |
| --- | --- | --- | --- | --- | --- | --- | --- | --- | --- | --- | --- | --- |
| **№ of studies** | **Study design** | **Risk of bias** | **Inconsistency** | **Indirectness** | **Imprecision** | **Other considerations** | **[IOI]** | **[NDI]** | **Relative (95% CI)** | **Absolute (95% CI)** |  |  |
| 3 | randomised trials | very serious^a^ | not serious | not serious | serious^b^ | none | 229/341 (67.2%) | 211/338 (62.4%) | **OR 1.28** (0.84 to 1.94) | **56 more per 1,000** (from 42 fewer to 139 more) | ⨁◯◯◯ Very low^a,b^ |  |

**CI:** confidence interval; **OR:** odds ratio

#### Explanations

a. Deviations from the intended interventions; Selection of the reported result

b. Downgraded one level for serious imprecision: the 95% CI (0.84–1.94) crosses the line of no effect and includes both little or no effect and a potentially important benefit, with a limited total sample size.

**Question:** [TCI] compared to [NDI] for [Consistent condom use]

| **Certainty assessment** | | | | | | | **№ of patients** | | **Effect** | | **Certainty** | **Importance** |
| --- | --- | --- | --- | --- | --- | --- | --- | --- | --- | --- | --- | --- |
| **№ of studies** | **Study design** | **Risk of bias** | **Inconsistency** | **Indirectness** | **Imprecision** | **Other considerations** | **[TCI]** | **[NDI]** | **Relative (95% CI)** | **Absolute (95% CI)** |  |  |
| 3 | randomised trials | serious^a^ | not serious | not serious | serious^b^ | none | 71/250 (28.4%) | 124/346 (35.8%) | **OR 0.81** (0.56 to 1.18) | **47 fewer per 1,000** (from 120 fewer to 39 more) | ⨁⨁◯◯ Low^a,b^ |  |

**CI:** confidence interval; **OR:** odds ratio

#### Explanations

a. Deviations from the intended interventions

b. CI crosses the invalid line (OR=1), and one end has reached "meaningful benefit" and the other end is close to "meaningful harm".

**Question:** [MAI] compared to [NDI] for [Consistent condom use]

| **Certainty assessment** | | | | | | | **№ of patients** | | **Effect** | | **Certainty** | **Importance** |
| --- | --- | --- | --- | --- | --- | --- | --- | --- | --- | --- | --- | --- |
| **№ of studies** | **Study design** | **Risk of bias** | **Inconsistency** | **Indirectness** | **Imprecision** | **Other considerations** | **[MAI]** | **[NDI]** | **Relative (95% CI)** | **Absolute (95% CI)** |  |  |
| 2 | randomised trials | not serious | serious^a^ | not serious | very serious^b^ | none | 319/379 (84.2%) | 449/529 (84.9%) | **OR 1.16** (0.52 to 2.56) | **18 more per 1,000** (from 104 fewer to 86 more) | ⨁◯◯◯ Very low^a,b^ |  |

**CI:** confidence interval; **OR:** odds ratio

#### Explanations

a. I² =59.8%, there was moderate heterogeneity (I² ≈ 60%) with considerable variation in effect sizes across studies that could not be fully explained by prespecified subgroup or sensitivity analyses.

b. Downgraded two levels for very serious imprecision: the 95% CI (0.52–2.56) is wide, crosses the line of no effect, and includes both important harm and substantial benefit, with a limited total sample size.

**Proportion of condom use**

**Question:** [IOI] compared to [SWI] for [Proportion of condom use]

| **Certainty assessment** | | | | | | | **№ of patients** | | **Effect** | | **Certainty** | **Importance** |
| --- | --- | --- | --- | --- | --- | --- | --- | --- | --- | --- | --- | --- |
| **№ of studies** | **Study design** | **Risk of bias** | **Inconsistency** | **Indirectness** | **Imprecision** | **Other considerations** | **[IOI]** | **[SWI]** | **Relative (95% CI)** | **Absolute (95% CI)** |  |  |
| 1 | randomised trials | not serious | not serious | not serious | serious^a^ | none | 231/366 (63.1%) | 219/391 (56.0%) | **OR 1.34** (1.00 to 1.80) | **70 more per 1,000** (from 0 fewer to 136 more) | ⨁⨁⨁◯ Moderate^a^ |  |

**CI:** confidence interval; **OR:** odds ratio

#### Explanations

a. the 95% CI (1.00–1.80) includes the possibility of no important effect and up to a moderately important benefit, and the evidence is based on a single study with limited sample size.

**Question:** [TCI] compared to [NDI] for [Proportion of condom use]

| **Certainty assessment** | | | | | | | **№ of patients** | | **Effect** | | **Certainty** | **Importance** |
| --- | --- | --- | --- | --- | --- | --- | --- | --- | --- | --- | --- | --- |
| **№ of studies** | **Study design** | **Risk of bias** | **Inconsistency** | **Indirectness** | **Imprecision** | **Other considerations** | **[TCI]** | **[NDI]** | **Relative (95% CI)** | **Absolute (95% CI)** |  |  |
| 1 | randomised trials | not serious | not serious | not serious | very serious^a^ | none | 19/27 (70.4%) | 19/28 (67.9%) | **OR 1.13** (0.36 to 3.53) | **26 more per 1,000** (from 247 fewer to 203 more) | ⨁⨁◯◯ Low^a^ |  |

**CI:** confidence interval; **OR:** odds ratio

#### Explanations

a. the 95% CI (0.36–3.53) is very wide, crosses the line of no effect, and includes both important harm and substantial benefit, based on a single study with limited sample size.

**Question:** [MAI] compared to [SWI] for [Proportion of condom use]

| **Certainty assessment** | | | | | | | **№ of patients** | | **Effect** | | **Certainty** | **Importance** |
| --- | --- | --- | --- | --- | --- | --- | --- | --- | --- | --- | --- | --- |
| **№ of studies** | **Study design** | **Risk of bias** | **Inconsistency** | **Indirectness** | **Imprecision** | **Other considerations** | **[MAI]** | **[SWI]** | **Relative (95% CI)** | **Absolute (95% CI)** |  |  |
| 1 | randomised trials | not serious | not serious | not serious | not serious | none | 70/95 (73.7%) | 15/28 (53.6%) | **OR 2.43** (1.01 to 5.80) | **201 more per 1,000** (from 2 more to 334 more) | ⨁⨁⨁⨁ High |  |

**CI:** confidence interval; **OR:** odds ratio

**Question:** [MAI] compared to [NDI] for [Proportion of condom use]

| **Certainty assessment** | | | | | | | **№ of patients** | | **Effect** | | **Certainty** | **Importance** |
| --- | --- | --- | --- | --- | --- | --- | --- | --- | --- | --- | --- | --- |
| **№ of studies** | **Study design** | **Risk of bias** | **Inconsistency** | **Indirectness** | **Imprecision** | **Other considerations** | **[MAI]** | **[NDI]** | **Relative (95% CI)** | **Absolute (95% CI)** |  |  |
| 1 | randomised trials | not serious | not serious | not serious | very serious^a^ | none | 19/21 (90.5%) | 20/21 (95.2%) | **OR 0.48** (0.04 to 5.68) | **47 fewer per 1,000** (from 508 fewer to 39 more) | ⨁⨁◯◯ Low^a^ |  |

**CI:** confidence interval; **OR:** odds ratio

#### Explanations

a. the 95% CI (0.04–5.68) is extremely wide, crosses the line of no effect, and includes both important harm and very large benefit, based on a single small study.

**Question:** [SWI] compared to [NDI] for [Proportion of condom use]

| **Certainty assessment** | | | | | | | **№ of patients** | | **Effect** | | **Certainty** | **Importance** |
| --- | --- | --- | --- | --- | --- | --- | --- | --- | --- | --- | --- | --- |
| **№ of studies** | **Study design** | **Risk of bias** | **Inconsistency** | **Indirectness** | **Imprecision** | **Other considerations** | **[SWI]** | **[NDI]** | **Relative (95% CI)** | **Absolute (95% CI)** |  |  |
| 2 | randomised trials | serious^a^ | not serious | not serious | very serious^b^ | none | 104/144 (72.2%) | 84/127 (66.1%) | **OR 1.16** (0.69 to 1.98) | **32 more per 1,000** (from 87 fewer to 133 more) | ⨁◯◯◯ Very low^a,b^ |  |

**CI:** confidence interval; **OR:** odds ratio

#### Explanations

a. Deviations from the intended interventions

b. Downgraded two levels for very serious imprecision: 95% CI (0.69–1.98) includes important harm and large benefit; total sample size is limited.

**The incidence rate of STIs (including HIV)**

**Question:** [TCI] compared to [NDI] for [The incidence rate of STIs (including HIV)]

| **Certainty assessment** | | | | | | | **№ of patients** | | **Effect** | | **Certainty** | **Importance** |
| --- | --- | --- | --- | --- | --- | --- | --- | --- | --- | --- | --- | --- |
| **№ of studies** | **Study design** | **Risk of bias** | **Inconsistency** | **Indirectness** | **Imprecision** | **Other considerations** | **[TCI]** | **[NDI]** | **Relative (95% CI)** | **Absolute (95% CI)** |  |  |
| 1 | randomised trials | not serious | not serious | not serious | serious^a^ | none | 693/3123 (22.2%) | 647/3125 (20.7%) | **OR 1.11** (0.98 to 1.25) | **18 more per 1,000** (from 3 fewer to 39 more) | ⨁⨁⨁◯ Moderate^a^ |  |

**CI:** confidence interval; **OR:** odds ratio

#### Explanations

a. the 95% CI (0.98–1.25) narrowly crosses the line of no effect and includes both little or no effect and a potentially important benefit, based on a single study with limited sample size.

**Question:** [IOI] compared to [NDI] for [The incidence rate of STIs (including HIV)]

| **Certainty assessment** | | | | | | | **№ of patients** | | **Effect** | | **Certainty** | **Importance** |
| --- | --- | --- | --- | --- | --- | --- | --- | --- | --- | --- | --- | --- |
| **№ of studies** | **Study design** | **Risk of bias** | **Inconsistency** | **Indirectness** | **Imprecision** | **Other considerations** | **[IOI]** | **[NDI]** | **Relative (95% CI)** | **Absolute (95% CI)** |  |  |
| 2 | randomised trials | very serious^a^ | very serious^b^ | not serious | very serious^c^ | none | 310/1640 (18.9%) | 210/1398 (15.0%) | **OR 0.92** (0.35 to 2.42) | **10 fewer per 1,000** (from 92 fewer to 149 more) | ⨁◯◯◯ Very low^a,b,c^ |  |

**CI:** confidence interval; **OR:** odds ratio

#### Explanations

a. Deviations from the intended interventions；Missing outcome data；Measurement of the outcome；Selection of the reported result

b. Downgraded two levels for very serious inconsistency: there was substantial between-study heterogeneity (I² = 85.3%) with widely varying effect estimates that could not be adequately explained by prespecified subgroup or sensitivity analyses.

c. Downgraded two levels for very serious imprecision: the 95% CI (0.35–2.42) is very wide, crosses the line of no effect, and includes both important harm and substantial benefit, with a limited total sample size.

**Question:** [SWI] compared to [NDI] for [The incidence rate of STIs (including HIV)]

| **Certainty assessment** | | | | | | | **№ of patients** | | **Effect** | | **Certainty** | **Importance** |
| --- | --- | --- | --- | --- | --- | --- | --- | --- | --- | --- | --- | --- |
| **№ of studies** | **Study design** | **Risk of bias** | **Inconsistency** | **Indirectness** | **Imprecision** | **Other considerations** | **[SWI]** | **[NDI]** | **Relative (95% CI)** | **Absolute (95% CI)** |  |  |
| 2 | randomised trials | not serious | not serious | not serious | very serious^a^ | none | 74/631 (11.7%) | 98/644 (15.2%) | **OR 1.34** (0.53 to 3.35) | **42 more per 1,000** (from 65 fewer to 223 more) | ⨁⨁◯◯ Low^a^ |  |

**CI:** confidence interval; **OR:** odds ratio

#### Explanations

a. Downgraded two levels for very serious imprecision: the 95% CI (0.53–3.35) is very wide, crosses the line of no effect, and includes both important harm and substantial benefit, with a limited total sample size.

**Question:** [IOI] compared to [SWI] for [The incidence rate of STIs (including HIV)]

| **Certainty assessment** | | | | | | | **№ of patients** | | **Effect** | | **Certainty** | **Importance** |
| --- | --- | --- | --- | --- | --- | --- | --- | --- | --- | --- | --- | --- |
| **№ of studies** | **Study design** | **Risk of bias** | **Inconsistency** | **Indirectness** | **Imprecision** | **Other considerations** | **[IOI]** | **[SWI]** | **Relative (95% CI)** | **Absolute (95% CI)** |  |  |
| 2 | randomised trials | serious^a^ | not serious | not serious | very serious^b^ | none | 44/1140 (3.9%) | 34/1155 (2.9%) | **OR 0.74** (0.43 to 1.25) | **7 fewer per 1,000** (from 17 fewer to 7 more) | ⨁◯◯◯ Very low^a,b^ |  |

**CI:** confidence interval; **OR:** odds ratio

#### Explanations

a. Deviations from the intended interventions；Measurement of the outcome；Selection of the reported result

b. Downgraded two levels for very serious imprecision: the 95% CI (0.43–1.25) is wide, crosses the line of no effect, and includes both important harm and substantial benefit, with a limited total sample size.
