# Supplementary material for: Effects of Digital Health Interventions to Promote Safer Sex Behaviors Among Youth: Systematic Review and Bayesian Network Meta-Analysis
Source: J Med Internet Res. 2026 Feb 4;28:e87071. doi: 10.2196/87071 (PMC12871581; doi:10.2196/87071)
Supplement: Multimedia Appendix 4 [file jmir-v28-e87071-s004.docx]

**Reference list of included studies**

1. Zhenchao Hu, 2023

- Hu Z, Fu Y, Wang X, et al. Effects of Sexuality Education on Sexual Knowledge, Sexual Attitudes, and Sexual Behaviors of Youths in China: A Cluster-Randomized Controlled Trial. J Adolesc Health. 2023;72(4):607-615. doi:10.1016/j.jadohealth.2022.11.006

2. Rayner Kay Jin Tan, 2022

- Tan RKJ, Koh WL, Le D, et al. Effect of a Popular Web Drama Video Series on HIV and Other Sexually Transmitted Infection Testing Among Gay, Bisexual, and Other Men Who Have Sex With Men in Singapore: Community-Based, Pragmatic, Randomized Controlled Trial. J Med Internet Res. 2022;24(5). doi:10.2196/31401

3. Elly Nuwamanya, 2020

- Nuwamanya E, Nalwanga R, Nuwasiima A, et al. Effectiveness of a mobile phone application to increase access to sexual and reproductive health information, goods, and services among university students in Uganda: a randomized controlled trial. Contracept Reprod Med. 2020;5(1). doi:10.1186/s40834-020-00134-5

4. Emma Wilson, 2017

- Wilson E, Free C, Morris TP, et al. Internet-accessed sexually transmitted infection (e-STI) testing and results service: A randomised, single-blind, controlled trial. PLoS Med. 2017;14(12). doi:10.1371/journal.pmed.1002479

5. Joseph T. F. Lau, 2016

- Lau JTF, Lee AL, Tse WS, et al. A Randomized Control Trial for Evaluating Efficacies of Two Online Cognitive Interventions With and Without Fear-Appeal Imagery Approaches in Preventing Unprotected Anal Sex Among Chinese Men Who Have Sex with Men. AIDS Behav. 2016;20(9):1851-1862. doi:10.1007/s10461-015-1263-z

6. Rienke Bannink, 2014

- Bannink R, Broeren S, Joosten-van Zwanenburg E, van As E, van de Looij-Jansen P, Raat H. Effectiveness of a Web-based tailored intervention (E-health4Uth) and consultation to promote adolescents’ health: randomized controlled trial. J Med Internet Res. 2014;16(5):e143. doi:10.2196/jmir.3163

7. Megan S C Lim, 2011

- Lim MSC, Hocking JS, Aitken CK, et al. Impact of text and email messaging on the sexual health of young people: A randomised controlled trial. J Epidemiol Community Health. 2012;66(1):69-74. doi:10.1136/jech.2009.100396

8. Mary Jane Rotheram-Borus, 2004

- Rotheram-Borus MJ, Swendeman D, Comulada WS, Weiss RE, Lee M, Lightfoot M. Prevention for substance-using HIV-positive young people: telephone and in-person delivery. J Acquir Immune Defic Syndr 1999. 2004;37 Suppl 2:S68-77.

9. Rafael Ballester-Arnal, 2015

- Ballester-Arnal R, Gil-Llario MD, Giménez-García C, Kalichman SC. What Works Well in HIV Prevention Among Spanish Young People? An Analysis of Differential Effectiveness Among Six Intervention Techniques. AIDS Behav. 2015;19(7):1157-1169. doi:10.1007/s10461-014-0863-3

10. Caroline Free, 2022

- Free C, Palmer MJ, McCarthy OL, et al. Effectiveness of a behavioural intervention delivered by text messages (safetxt) on sexually transmitted reinfections in people aged 16-24 years: randomised controlled trial. BMJ. 2022;378:e070351. doi:10.1136/bmj-2022-070351

11. Diane Santa Maria, 2021

- Santa Maria D, Padhye N, Businelle M, et al. Efficacy of a Just-in-Time Adaptive Intervention to Promote HIV Risk Reduction Behaviors Among Young Adults Experiencing Homelessness: Pilot Randomized Controlled Trial. J Med Internet Res. 2021;23(7):e26704. doi:10.2196/26704

12. Laura B. Whiteley, 2018

- Whiteley LB, Brown LK, Curtis V, Ryoo HJ, Beausoleil N. Publicly Available Internet Content as a HIV/STI Prevention Intervention for Urban Youth. J Prim Prev. 2018;39(4):361-370. doi:10.1007/s10935-018-0514-y

13. Brian Mustanski, 2018

- Mustanski B, Parsons JT, Sullivan PS, Madkins K, Rosenberg E, Swann G. Biomedical and Behavioral Outcomes of Keep It Up!: An eHealth HIV Prevention Program RCT. Am J Prev Med. 2018;55(2):151-158. doi:10.1016/j.amepre.2018.04.026

14. Peipert JF, 2008

- Peipert JF, Redding CA, Blume JD, et al. Tailored intervention to increase dual-contraceptive method use: a randomized trial to reduce unintended pregnancies and sexually transmitted infections. Am J Obstet Gynecol. 2008;198(6):630.e1-8. doi:10.1016/j.ajog.2008.01.038

15. Sheana Bull, 2016

- Bull S, Devine S, Schmiege SJ, Pickard L, Campbell J, Shlay JC. Text Messaging, Teen Outreach Program, and Sexual Health Behavior: A Cluster Randomized Trial. Am J Public Health. 2016;106(S1):S117-S124.

16. Michele L. Ybarra, 2013

- Ybarra ML, Bull SS, Prescott TL, Korchmaros JD, Bangsberg DR, Kiwanuka JP. Adolescent Abstinence and Unprotected Sex in CyberSenga, an Internet-Based HIV Prevention Program: Randomized Clinical Trial of Efficacy. PLOS ONE. 2013;8(8). doi:10.1371/journal.pone.0070083

17. José A. Bauermeister, 2019

- Bauermeister JA, Tingler RC, Demers M, et al. Acceptability and Preliminary Efficacy of an Online HIV Prevention Intervention for Single Young Men Who Have Sex with Men Seeking Partners Online: The myDEx Project. AIDS Behav. 2019;23(11):3064-3077. doi:10.1007/s10461-019-02426-7

18. Deborah J Rinehart, 2020

- Rinehart DJ, Leslie S, Durfee MJ, et al. Acceptability and Efficacy of a Sexual Health Texting Intervention Designed to Support Adolescent Females. Acad Pediatr. 2020;20(4):475-484. doi:10.1016/j.acap.2019.09.004

19. Melissa K. Miller, 2021

- Miller MK, Catley D, Adams A, et al. Brief Motivational Intervention to Improve Adolescent Sexual Health Service Uptake: A Pilot Randomized Controlled Trial in the Emergency Department. J Pediatr. 2021;237:250-257.e2. doi:10.1016/j.jpeds.2021.06.007

20. David Cordova, 2020

- Cordova D, Munoz-Velazquez J, Mendoza Lua F, et al. Pilot Study of a Multilevel Mobile Health App for Substance Use, Sexual Risk Behaviors, and Testing for Sexually Transmitted Infections and HIV Among Youth: Randomized Controlled Trial. JMIR MHealth UHealth. 2020;8(3):e16251. doi:10.2196/16251

21. Taraneh Shafii, 2019

- Shafii T, Benson SK, Morrison DM, Hughes JP, Golden MR, Holmes KK. Results from e-KISS: electronic-KIOSK Intervention for Safer Sex: A pilot randomized controlled trial of an interactive computer-based intervention for sexual health in adolescents and young adults. PloS One. 2019;14(1):e0209064. doi:10.1371/journal.pone.0209064

22. Lauren S. Chernick, 2022

- Chernick LS, Santelli J, Stockwell MS, et al. A multi-media digital intervention to improve the sexual and reproductive health of female adolescent emergency department patients. Acad Emerg Med Off J Soc Acad Emerg Med. 2022;29(3):308-316. doi:10.1111/acem.14411

23. Jennifer Yarger, 2024

- Yarger J, Gutmann-Gonzalez A, Borgen N, Romero J, Decker MJ. In the Know: A Cluster Randomized Trial of an In-person Sexual Health Education Program Integrating Digital Technologies for Adolescents. J Adolesc Health Off Publ Soc Adolesc Med. 2024;74(5):1019-1025. doi:10.1016/j.jadohealth.2023.12.012

24. Brian Suffoletto, 2013

- Suffoletto B, Akers A, McGinnis KA, Calabria J, Wiesenfeld HC, Clark DB. A Sex Risk Reduction Text-Message Program for Young Adult Females Discharged From the Emergency Department. J Adolesc Health. 2013;53(3):387-393. doi:10.1016/j.jadohealth.2013.04.006
