# Supplementary material for: Effects of Digital Health Interventions to Promote Safer Sex Behaviors Among Youth: Systematic Review and Bayesian Network Meta-Analysis
Source: J Med Internet Res. 2026 Feb 4;28:e87071. doi: 10.2196/87071 (PMC12871581; doi:10.2196/87071)
Supplement: Multimedia Appendix 5 [file jmir-v28-e87071-s005.docx]

**Indirect comparative effectiveness DHIs**

| **Increase in condom use in the last sexual contact** |  |  |  |
| --- | --- | --- | --- |
| **MAI** |  |  |  |
| 1.08 (0.78, 1.51) | **TCI** |  |  |
| 1.23 (0.90, 1.68) | 1.13 (1.02, 1.26) | **NDI** |  |
| 1.58 (0.82, 2.98) | 1.45 (0.81, 2.59) | 1.28 (0.72, 2.25) | **IOI** |

| **Increase in consistent condom use** | |  |  |  |  |
| --- | --- | --- | --- | --- | --- |
| **SWI** |  |  |  |  |  |
| 1.06 (0.78, 1.44) | **IOI** |  |  |  |  |
| 1.43 (0.97, 2.12) | 1.35 (0.98, 1.88) | **NDI** |  |  |  |
| 1.48 (0.86, 2.54) | 1.40 (0.85, 2.30) | 1.03 (0.72, 1.49) | **MAI** |  |  |
| 1.77 (1.03, 3.06) | 1.68 (1.02, 2.76) | 1.24 (0.85, 1.80) | 1.20 (0.71, 2.03) | **TCI** |  |

| **Increase in** **proportion of protected acts** |  |  |  |  |
| --- | --- | --- | --- | --- |
| **MAI** |  |  |  |  |
| 1.50 (0.61, 3.63) | **IOI** |  |  |  |
| 1.91 (0.41, 8.67) | 1.27 (0.34, 4.78) | **TCI** |  |  |
| 2.01 (0.87, 4.67) | 1.34 (1.01, 1.80) | 1.05 (0.29, 3.86) | **SWI** |  |
| 2.17 (0.84, 5.72) | 1.44 (0.80, 2.66) | 1.13 (0.36, 3.72) | 1.08 (0.64,1.84) | **NDI** |

| **Reduction in the incidence rate of STIs (including HIV)** |  |  |  |
| --- | --- | --- | --- |
| **NDI** |  |  |  |
| 0.92 (0.81, 1.03) | **TCI** |  |  |
| 0.78 (0.65, 0.93) | 0.85 (0.68, 1.06) | **IOI** |  |
| 0.61 (0.46, 0.82) | 0.67 (0.49, 0.92) | 0.79 (0.60, 1.04) | **SWI** |

Results are the mean differences and their related 95% confidence intervals in the column-defining intervention compared with the mean differences in the row-defining intervention. Mean difference lower than zero favours the column-defining intervention. Significant results are in bold.
