# Supplementary material for: Effects of Digital Health Interventions to Promote Safer Sex Behaviors Among Youth: Systematic Review and Bayesian Network Meta-Analysis
Source: J Med Internet Res. 2026 Feb 4;28:e87071. doi: 10.2196/87071 (PMC12871581; doi:10.2196/87071)
Supplement: Multimedia Appendix 6 [file jmir-v28-e87071-s006.docx]

**Appendix 5.** Forest plots of posterior ORs with 95% CrIs for each DHIs compared with NDI.


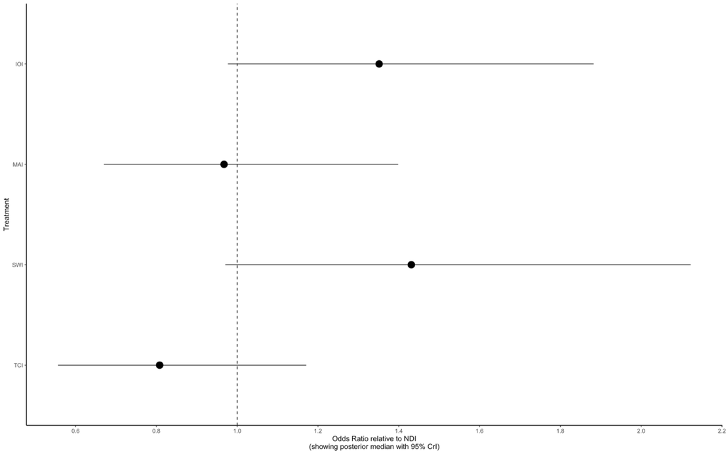

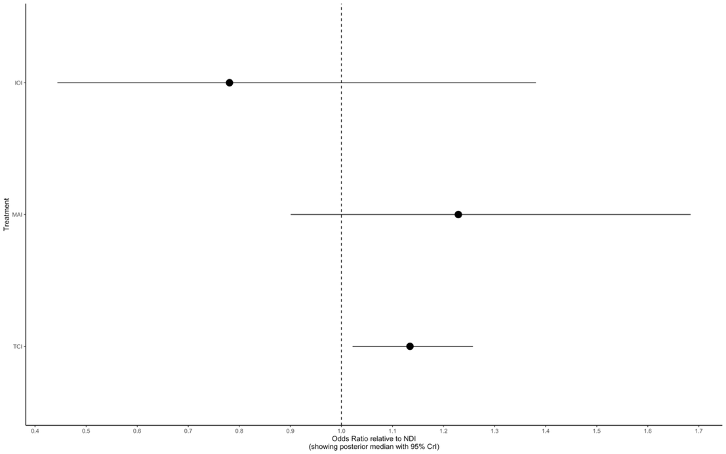


**(B)**

**(A)**

**(D)**

**(C)**


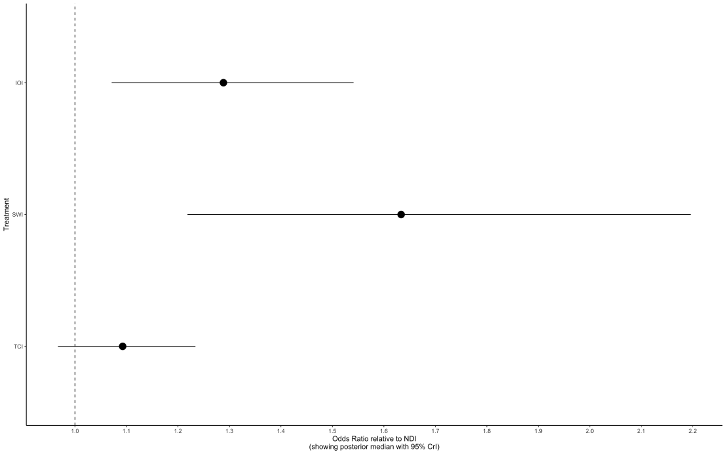

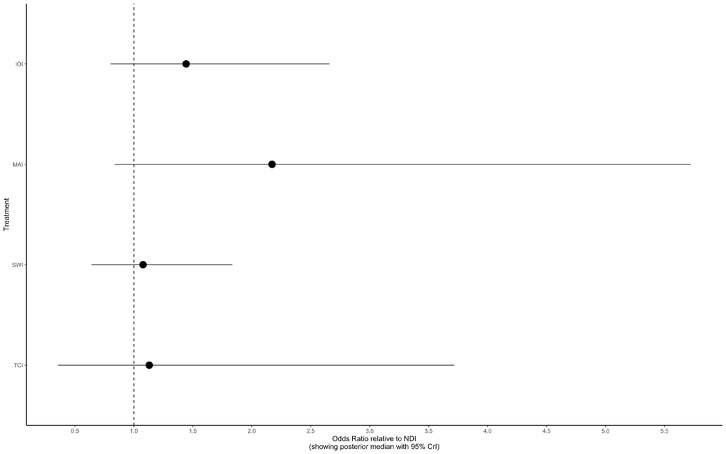


*Note:* Panels show (A) Condom use rate in the last sexual contact; (B) Consistent condom use rate; (C) Proportion of condom use; and (D) The incidence rate of STIs (including HIV). Black dots represent posterior median *ORs*, horizontal lines represent 95% *CrIs*, and the vertical dashed line marks an *OR* of 1 (no difference versus NDI). For condom-use outcomes (A-C), *ORs* > 1 indicate higher condom use compared with NDI, whereas for STI incidence (D), *ORs* > 1 indicate higher infection rates.
