# Supplementary material for: Effects of Digital Health Interventions to Promote Safer Sex Behaviors Among Youth: Systematic Review and Bayesian Network Meta-Analysis
Source: J Med Internet Res. 2026 Feb 4;28:e87071. doi: 10.2196/87071 (PMC12871581; doi:10.2196/87071)
Supplement: Multimedia Appendix 7 [file jmir-v28-e87071-s007.docx]

**Random‐effects network meta‐analysis of DHIs vs NDIs: *ORs*, 95% *CIs*, and 95% *PIs* by outcome**

| **Outcome** | **Treatment** | ***OR*** | ***CI*_low** | ***CI*_high** | ***PI*_low** | ***PI*_high** |
| --- | --- | --- | --- | --- | --- | --- |
| **Condom use rate in the last sexual contact** | IOI | 0.78 | 0.45 | 1.37 | 0.35 | 1.74 |
|  | MAI | 1.23 | 0.9 | 1.68 | 0.79 | 1.91 |
|  | TCI | 1.13 | 1.02 | 1.26 | 0.98 | 1.32 |
| **Consistent condom use rate** | IOI | 1.35 | 0.92 | 1.96 | 0.74 | 2.46 |
|  | MAI | 1.01 | 0.64 | 1.6 | 0.51 | 2 |
|  | SWI | 1.45 | 0.91 | 2.32 | 0.73 | 2.9 |
|  | TCI | 0.8 | 0.52 | 1.24 | 0.42 | 1.55 |
| **Proportion of condom use** | IOI | 1.46 | 0.8 | 2.65 | 0.39 | 5.4 |
|  | MAI | 2.18 | 0.85 | 5.61 | 0.28 | 17.32 |
|  | SWI | 1.09 | 0.65 | 1.83 | 0.35 | 3.4 |
|  | TCI | 1.13 | 0.36 | 3.54 | 0.09 | 13.9 |
| **The incidence rate of STIs (including HIV)** | IOI | 0.99 | 0.57 | 1.7 | 0.25 | 3.86 |
|  | SWI | 1.34 | 0.75 | 2.39 | 0.33 | 5.41 |
|  | TCI | 1.09 | 0.49 | 2.44 | 0.22 | 5.43 |

*Note:* All estimates are from random-effects network meta-analysis with NDI as the reference group. *PIs* reflect the range of treatment effects expected in future studies given the observed between-study heterogeneity.
