# Supplementary material for: Effects of Digital Health Interventions to Promote Safer Sex Behaviors Among Youth: Systematic Review and Bayesian Network Meta-Analysis
Source: J Med Internet Res. 2026 Feb 4;28:e87071. doi: 10.2196/87071 (PMC12871581; doi:10.2196/87071)
Supplement: Multimedia Appendix 8 [file jmir-v28-e87071-s008.docx]

**Model fit diagnostics for consistency and inconsistency models in the network meta-analysis**

**(A)**

**(B)**


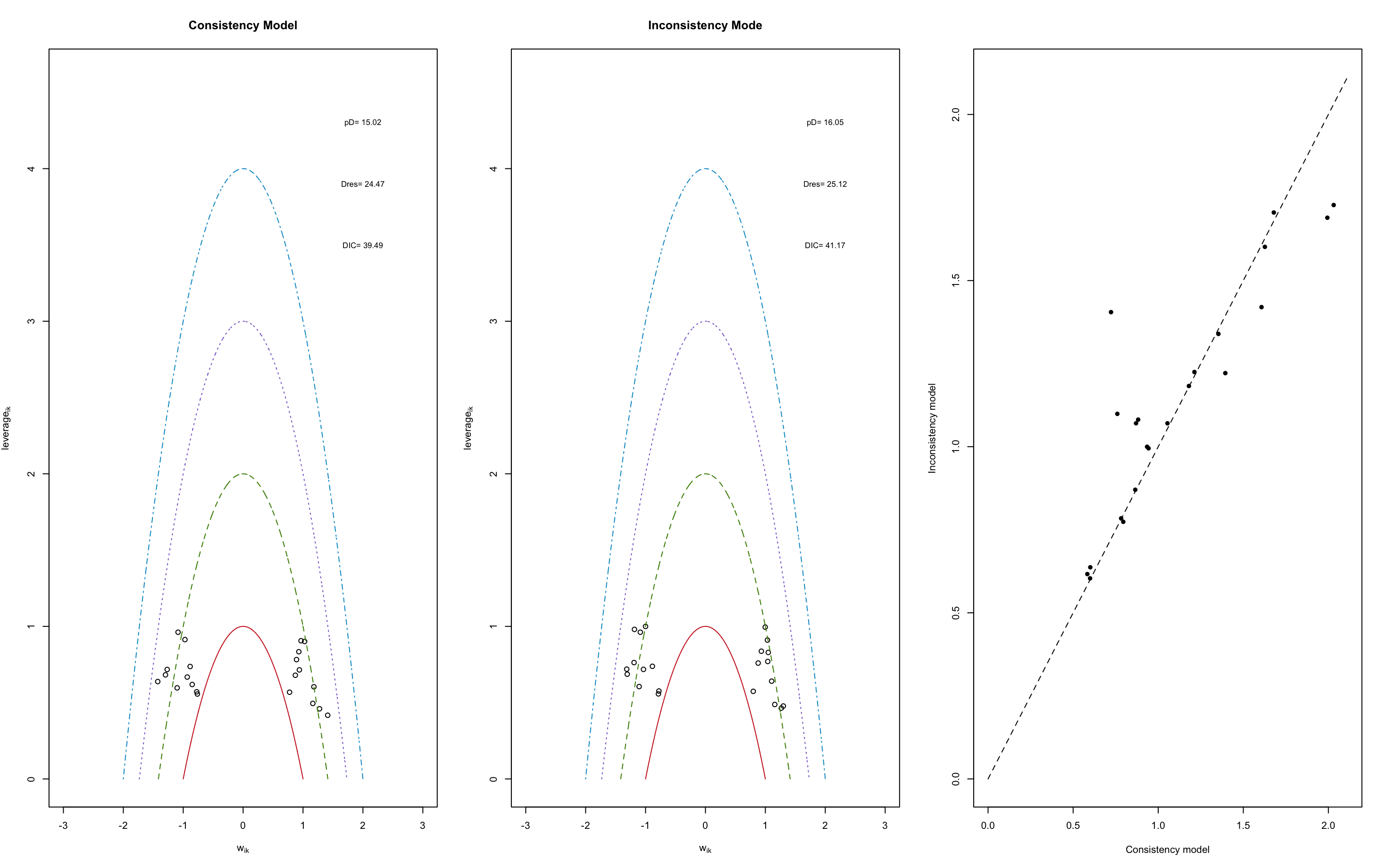

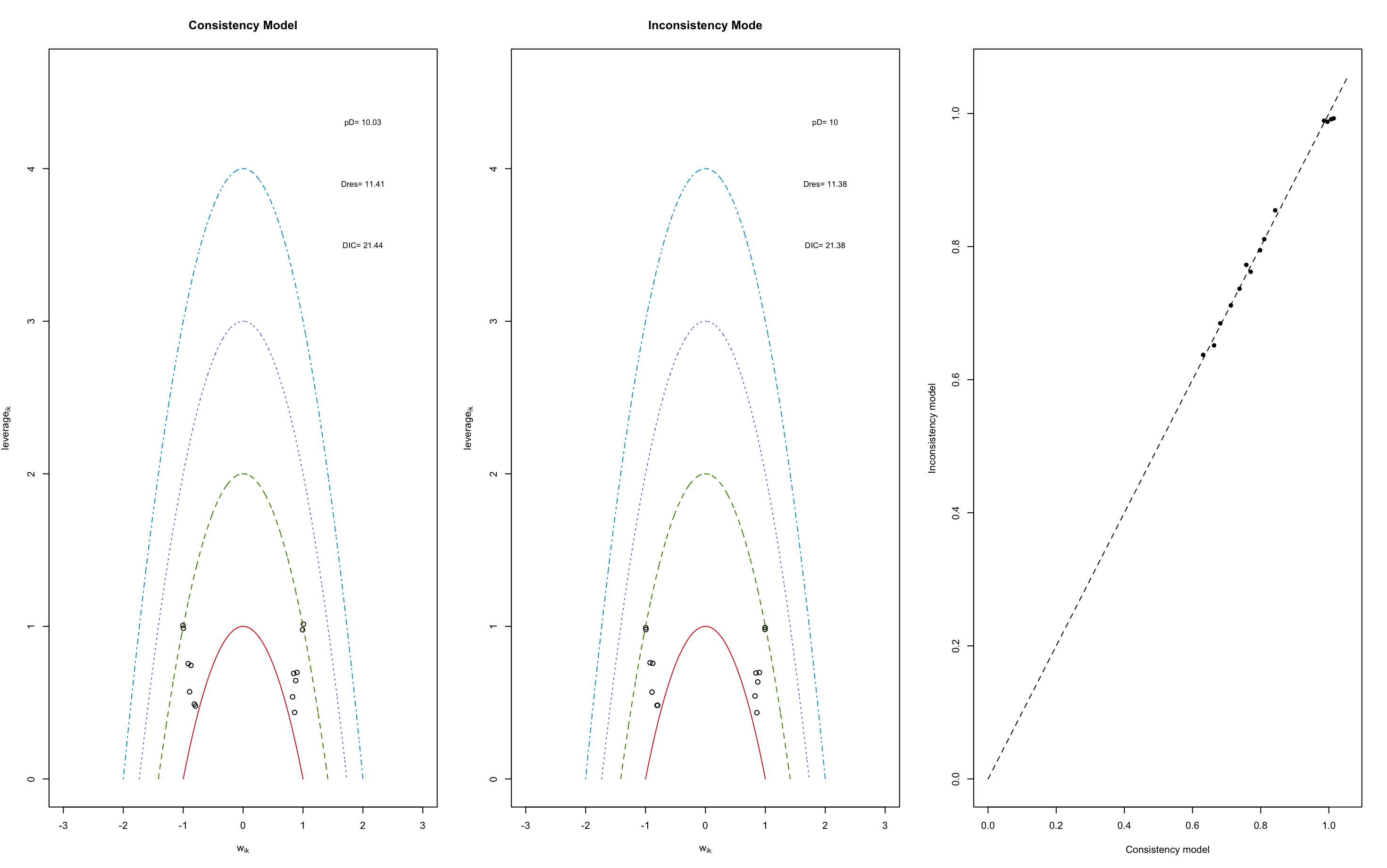


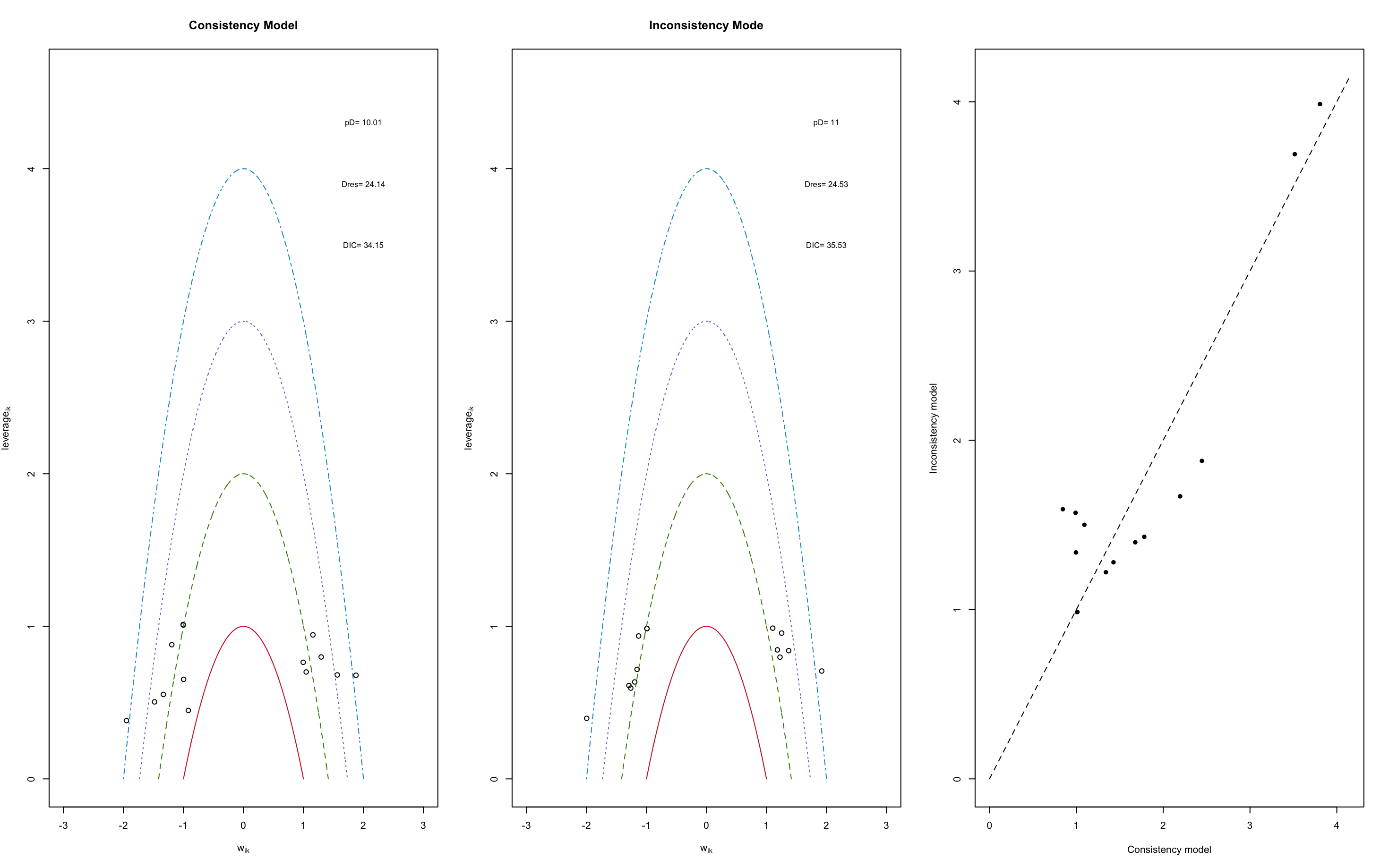

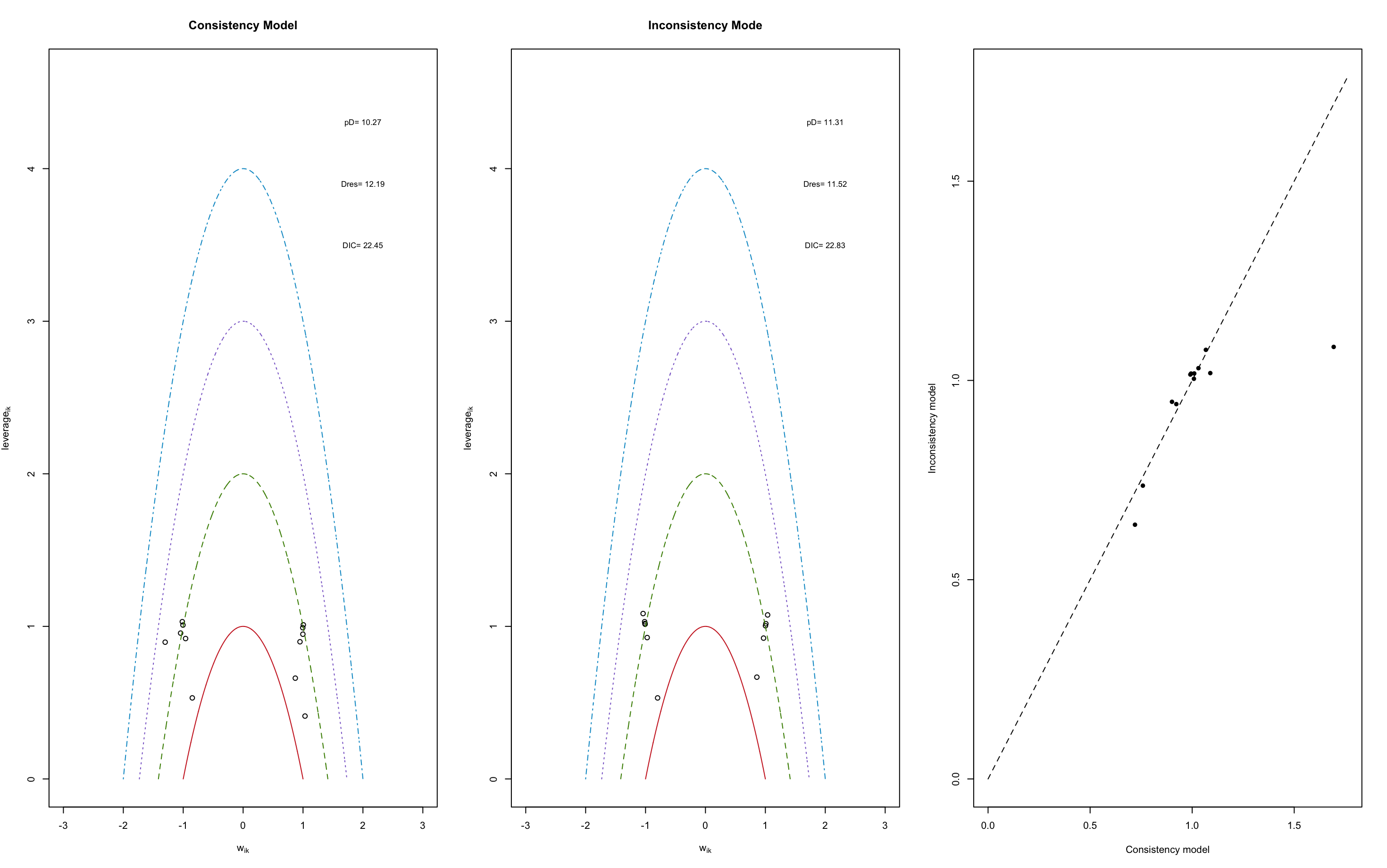


**(D)**

**(C)**

*Note:* Consistency between direct and indirect evidence was evaluated by comparing deviance information criterion (DIC) values from the consistency and inconsistency models; for all four outcomes, DIC differences were <5, indicating no evidence of global inconsistency in the network.
